# Nanotube‐like processes facilitate material transfer between photoreceptors

**DOI:** 10.15252/embr.202153732

**Published:** 2021-09-08

**Authors:** Aikaterini A Kalargyrou, Mark Basche, Aura Hare, Emma L West, Alexander J Smith, Robin R Ali, Rachael A Pearson

**Affiliations:** ^1^ University College London Institute of Ophthalmology London UK; ^2^ Centre for Cell and Gene Therapy King’s College London Guy’s Hospital London UK; ^3^ Kellogg Eye Center University of Michigan Ann Arbor MI USA

**Keywords:** extracellular vesicle, intercellular communication, material transfer, photoreceptor transplantation, tunnelling nanotube, Cell Adhesion, Polarity & Cytoskeleton, Membranes & Trafficking, Neuroscience

## Abstract

Neuronal communication is typically mediated via synapses and gap junctions. New forms of intercellular communication, including nanotubes (NTs) and extracellular vesicles (EVs), have been described for non‐neuronal cells, but their role in neuronal communication is not known. Recently, transfer of cytoplasmic material between donor and host neurons (“material transfer”) was shown to occur after photoreceptor transplantation. The cellular mechanism(s) underlying this surprising finding are unknown. Here, using transplantation, primary neuronal cultures and the generation of chimeric retinae, we show for the first time that mammalian photoreceptor neurons can form open‐end NT‐like processes. These processes permit the transfer of cytoplasmic and membrane‐bound molecules in culture and after transplantation and can mediate gain‐of‐function in the acceptor cells. Rarely, organelles were also observed to transfer. Strikingly, use of chimeric retinae revealed that material transfer can occur between photoreceptors in the intact adult retina. Conversely, while photoreceptors are capable of releasing EVs, at least in culture, these are taken up by glia and not by retinal neurons. Our findings provide the first evidence of functional NT‐like processes forming between sensory neurons in culture and *in vivo*.

## Graphical abstract

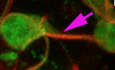

Mammalian photoreceptor neurons form open‐end nanotubes that mediate the exchange of cytoplasmic and membrane‐bound molecules, or material transfer, which can result in gain of function in the acceptor cells.

## Introduction

Intercellular communication is an essential process for the development and maintenance of all tissues, including the nervous system. Typically, cells employ two ways of communication, either via contacting directly (e.g. synaptic transmission) or by releasing molecular information in the extracellular fluid. Recently, new mechanisms of molecular exchange between cells have been described and include, respectively, the formation of membranous tubes between cells, called nanotubes (NTs), and the release and uptake of extracellular vesicles (EVs) (Rajendran *et al*, 2014; Cordero Cervantes & Zurzolo, 2021; Ljubojevic *et al*, 2021).

Previously regarded as part of the cell’s ‘garbage disposal system’, EVs can carry cytosolic and membrane proteins and potentially even genetic material, which have been reported to alter acceptor cell function in culture and *in vivo* (Kowal *et al*, 2016; Pastuzyn *et al*, 2018; van Niel *et al*, 2018). EVs are released by almost every cell type (van Niel *et al*, 2018), including neurons and glia (Faure *et al*, 2006; Kramer‐Albers *et al*, 2007; Chivet *et al*, 2013; Ibanez *et al*, 2019), and may originate from the endosomal pathway or by simply budding off the plasma membrane (Kowal *et al*, 2016; Verweij *et al*, 2018). Similarly, a variety of membranous processes have been described in diverse organisms, including echinoids (where they have been termed as specialized filopodia) (Gustafson & Wolpert, 1967), flies (cytonemes) (Ramirez‐Weber & Kornberg, 1999), birds (cytoplasmic bridges) (Teddy & Kulesa, 2004; George *et al*, 2016) and mammals (nanotubes) (Rustom *et al*, 2004; Chinnery *et al*, 2008). These processes can facilitate the exchange of molecules in culture and *in vivo* during early embryo development (for reviews, see (Korenkova *et al*, 2020; Ljubojevic *et al*, 2021)). Existing either as actin‐enriched open‐end tubes or closed‐tip filopodia‐like protrusions, these processes have been reported to transfer Ca^2+^ (Alarcon‐Martinez *et al*, 2020), morphogens (Chen *et al*, 2017), fluorescent reporters (Kulesa *et al*, 2010; McKinney & Kulesa, 2011), vesicles (Gradilla *et al*, 2014), mRNA (Haimovich *et al*, 2017) and even organelles (Rustom *et al*, 2004; Alarcon‐Martinez *et al*, 2020) between cells.

Investigations into NT function in mammals have largely been limited to *in vitro* studies, mostly due to the technical challenges associated with their visualization. These have led to postulated roles in many pathological conditions, including viral infection, cancer, neuropathies and prion‐associated disease (Gerdes & Carvalho, 2008; Gousset *et al*, 2009; Gerdes *et al*, 2013; Peralta *et al*, 2013; Tardivel *et al*, 2016). However, to our knowledge, there is only one, very recent, study reporting the presence of mammalian NT‐like processes *in vivo*, which were shown to form between retinal pericytes and mediate the exchange of Ca^2+^ signals and coordinate vascular contraction (Alarcon‐Martinez *et al*, 2020). While there are *in vitro* reports of NT‐mediated coupling from astrocytes to neurons (Wang *et al*, 2012b) and within mammalian neuronal cell lines (Sun *et al*, 2012; Tardivel *et al*, 2016), it is still not known whether similar structures can form between neurons *in vivo*.

Neuronal replacement by transplantation is proposed as a treatment for several neurodegenerative disorders. Previous studies, by us and others, have demonstrated the rescue of visual function following the transplantation of healthy photoreceptors into animal models of retinal disease (MacLaren *et al*, 2006; Lamba *et al*, 2009; Pearson *et al*, 2012; Barber *et al*, 2013; Zhu *et al*, 2017; Mahato *et al*, 2020). In end‐stage retinal disease, this rescue is achieved by donor cells forming new synaptic connections with host inner retinal neurons (Ribeiro *et al*, 2021). However, in partial degeneration, where some/all host photoreceptor cells remain, the transplantation of healthy donor photoreceptors into recipient eyes results in the specific and surprisingly efficient transfer of a wide array of both endogenous and transgenic molecules from donor to recipient photoreceptors in both normal and diseased retina, a phenomenon that has been termed ‘material transfer’ (Pearson *et al*, 2016; Santos‐Ferreira *et al*, 2016; Singh *et al*, 2016; Ortin‐Martinez *et al*, 2017). This extraordinary finding prompted us to determine the cellular mechanism(s) underlying this exchange and to explore whether this represents a novel mechanism of non‐synaptic intercellular communication between neurons, both within the normal and the diseased (treated) nervous system. We explored two leading but currently untested hypotheses (Pearson *et al*, 2016; Santos‐Ferreira *et al*, 2016; Singh *et al*, 2016; Ortin‐Martinez *et al*, 2017; Nickerson *et al*, 2018; Gasparini *et al*, 2019): (i) targeted release and uptake of extracellular vesicle (EVs) by photoreceptors, and (ii) physical connections between individual photoreceptor pairs, in the form of cytoplasmic bridges. Here, we used a combination of primary cell cultures, photoreceptor transplantation and the generation of chimeric retinae to elucidate the cellular mechanisms underlying material transfer between photoreceptors in culture and *in vivo*.

## Results

### EVs do not mediate material transfer between photoreceptors

**Figure 1:**
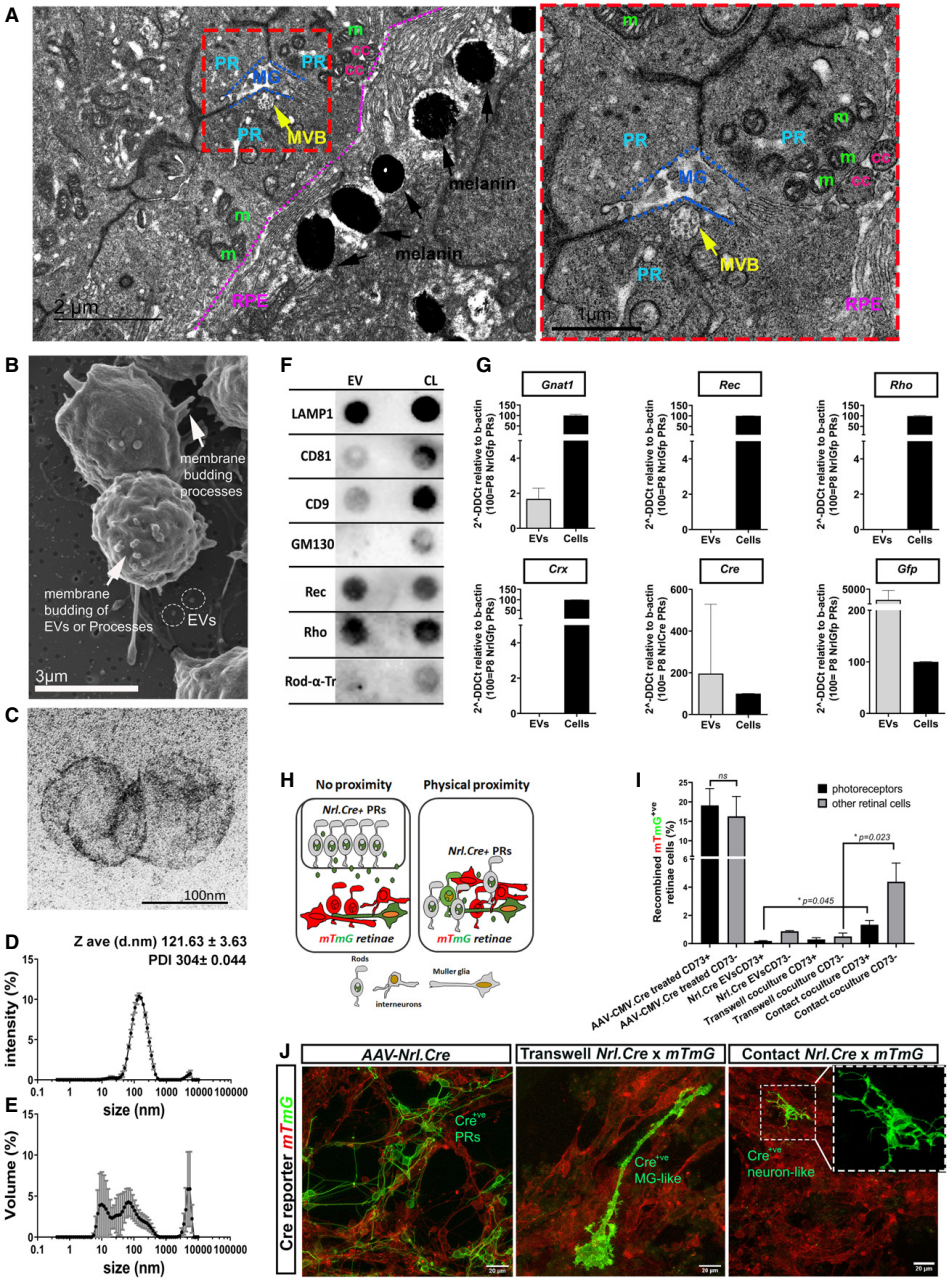
Primary photoreceptors release EVs that exert their function in Müller glial cells but not photoreceptors ATEM analysis of P7 wt retinae (*N* = 4 eyes) showing a (photoreceptor‐ Multivesicular Body) PR‐MVB in close proximity to (Muller glia) MG (red dashed box). PR‐photoreceptor cell, MG (blue dashed lines) ‐muller glia cell, MVB (yellow and yellow arrow)‐ multivesicular body, m (green)‐mitochondria, cc (pink)‐connecting cilium, RPE (purple and purple dashed line) ‐retina pigment epithelium, RPE melanin‐black arrows. Scale bar = 2 µm left, 1 µm right.BRepresentative SEM microphotograph of cultured P8 *Nrl.Gfp^+/+^* photoreceptors; arrows indicate membrane budding of processes or EVs; dashed circles denote EVs; Scale bar = 3 µm.CRepresentative TEM microphotograph of 100 K EV pellet derived from *Nrl.Gfp^+/+^* photoreceptor cultures (*N* = 10 independent preparations); Scale bar = 100 nm.D, ERepresentative dynamic light scatter (DLS) plot of 100 K EV sample (*N* = 13 samples), showing (D) average intensity and (E) volume against diameter (13 DLS measurements per sample).FRepresentative Dot blot of 100 K EV pellet (each dot represents three pooled EV isolations, derived from 60*10^6^ cells; *N* = 8 experiments) vs cell lysate (CL) from P8 *Nrl.Gfp^+/+^* photoreceptors. EV markers = LAMP1, CD8, CD9; Golgi marker = GM130; phototransduction markers = Recoverin, Rhodopsin, Rod α‐Transducin.GRT–qPCR analysis of 100 K P8 photoreceptor pellets for *Gnat1, Rec, Rho, Crx, Cre and Gfp,* relative to β‐actin, comparing EVs (*N* = 8) against the appropriate *(Nrl.Gfp^+/+^* or *Nrl.Cre^+/−^*) photoreceptor cell lysate (*N* = 3). *Gnat1*, *Cre* and *Gfp* mRNA were present in all relevant samples.HSchematic representation of *Cre‐LoxP* system to assess EV function in co‐culture of *Nrl.Cre^+/−^* photoreceptors (PRs) with *mTmG^floxed^* reporter retinal cells in non‐proximity (trans‐well) versus physical proximity (contact).IRepresentative flow cytometry analysis of *Nrl.Cre^+/−^ and mTmG^floxed^* co‐cultures. Samples were analysed for expression of myrGFP (recombination) and CD73 (photoreceptor identity) versus CD73^−ve^ fraction (other retinal cells). *N* > 3 independent cultures for each condition with technical triplicate for each sample; one‐way ANOVA, non‐parametric, Kruskal–Wallis with Dunns’ multiple comparisons test.JRepresentative maximum intensity projection (MIP) confocal images of *mTmG^floxed^* reporter retinal cells, (*left*) treated with *AAV‐Nrl.Cre* virus (positive control), (*middle*) co‐culture with *Nrl.Cre^+/−^* photoreceptors separated by trans‐well (non‐proximity) or (*right*) in contact; Scale bar = 20 µm; *Red* = *myrRFP‐*expressing *mTmG^floxed^* reporter, no recombination; *green* = *myrGFP‐*expressing *mTmG^floxed^* reporter, cells recombined upon acquiring Cre; *blue* = nuclei. *N* = 7 cultures. Data information: Graphs show mean ± SD.

We first explored the possibility that material transfer might be mediated by the release and uptake of EVs. We examined the ultrastructure of early postnatal wildtype retinae and found that the photoreceptors often presented with multivesicular bodies (MVBs; ˜ 500 nm diameter; 1–2 MVBs/photoreceptor at postnatal day 7) containing intraluminal vesicles (ILVs) (Fig 1A), which can be released as EVs. To study EV release and other photoreceptor–photoreceptor interactions in detail, we next established a primary culture system (see Methods and Kalargyrou *et al*, bioRxiv) that could support purified postnatal rod photoreceptors for many days, allowing them to extend processes and release vesicles into the surrounding media (Fig 1B). EVs were enriched from the cell culture media using the standard methodology of differential ultracentrifugation (Kowal *et al*, 2016; Thery *et al*, 2018). The 100 K pellet contained vesicles of ˜ 120 nm diameter, as determined by electron microscopy (EM) (Fig 1C) and dynamic light scattering (DLS) (Fig 1D and E) analysis, and presented with markers of the endocytic machinery, including LAMP1 (Fig 1F). The classical EV tetraspanins CD81, and CD9 were also present (Fig 1F), albeit not enriched, consistent with recent reassessments of EV composition (Jeppesen *et al*, 2019). Conversely, the Golgi marker GM130 was absent from the 100 K sample, confirming the lack of contamination from other organelles (Fig 1F).

**Figure EV1:**
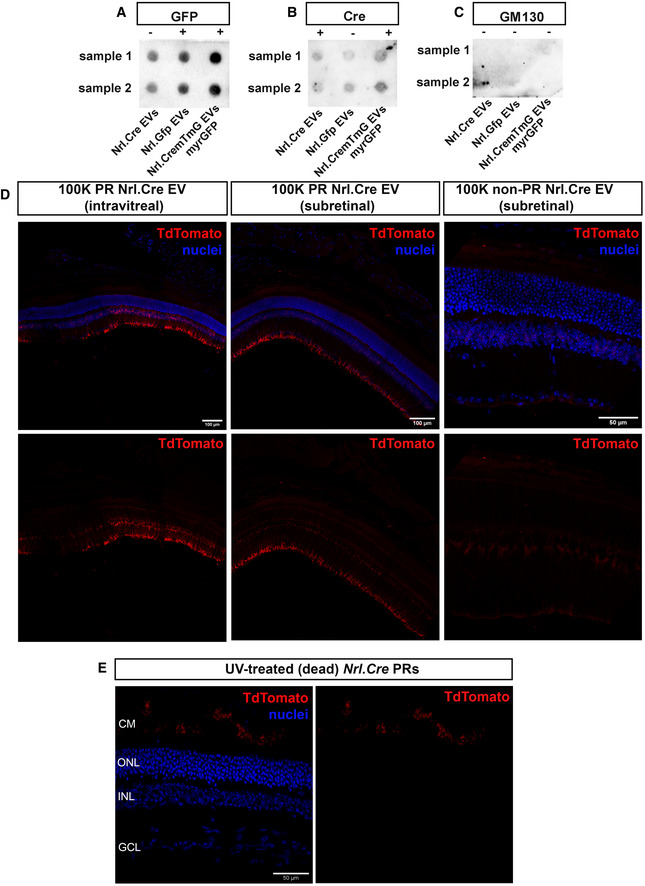
Photoreceptor‐derived EV subpopulations contain Cre and GFP protein A–CRepresentative dot blots of GFP (A), Cre (B) and GM130 (C) expression in P8 *Nrl.Cre^+/−^*, *Nrl.Gfp^+/+^*, *Nrl.Cre^+/−^* × *mTmG* ^+/+^ (*myrGFP*) photoreceptor‐derived 100 K EV pellets, as appropriate (*N* = 8 experiments, each dot represents a pool from three independent EV isolations, derived from 60*10^6^ cells from two samples). The lack of GM130 staining confirms the absence of contamination from Golgi within the EV preparations. Positive GM130 staining from whole cell lysate is shown in Fig 1.DRepresentative tile‐scan images following (*lef*t) subretinal and (*middle*) intravitreal injection of EVs (100 K fraction) derived from P8 *Nrl.Cre^+/−^* photoreceptors, compared with (*right*) subretinal injection of EVs (100 K fraction) derived from non‐photoreceptors (*Nrl.Cre^+/−^*); *red* = *TdTomato* recombined cells; *blue* = nuclei; Scale bar = 100 µm (*lef*t & middle) and 50 µm (right).

We next examined whether photoreceptor‐derived EVs contained cell‐type specific, functionally relevant cargo, alongside reporter molecules that would permit tracing of their transfer *in vitro* and *in vivo*. Specifically, we assessed molecules previously shown to be exchanged during material transfer following photoreceptor transplantation, including the fluorescent reporter GFP, Cre and Rod α‐transducin (Pearson *et al*, 2016; Waldron *et al*, 2018). Cell cultures were established from three transgenic mouse lines: *Nrl.Gfp^+/−^, Nrl.Cre^+/−^* and *Nrl.Cre^+/−^ x mTmG^floxed^*, which express cytoplasmic cGFP, Cre and myristoylated (myr)GFP, respectively. Use of the *Nrl* promoter ensures that expression is specific restricted to post‐mitotic rod photoreceptors in the retina (Akimoto *et al*, 2006). Dot blot analysis demonstrated the presence of the phototransduction‐related proteins Recoverin and Rhodopsin, but not Rod α‐transducin (Fig 1F). Cytoplasmic GFP, myrGFP and Cre protein were detected in the 100 K pellets derived from *Nrl.Gfp^+/+^*, *Nrl.Cre^+/−^ x mTmG* (membrane‐tagged GFP; Appendix Fig S1A and B) and *Nrl.Cre^+/−^* photoreceptors, respectively, but varied between samples (Fig EV1A–C). Conversely, *Gnat1* (rod α‐transducin), *Gfp* and *Cre* mRNA was readily detectable by qRT–PCR, while *Rhodopsin, Recoverin* and the photoreceptor‐specific transcription factor, *Crx,* were not detected (Fig 1G). Together, these data demonstrate that photoreceptor‐derived EVs contain cargo that differs from the composition of the cytoplasm of these cells.

We next examined the potential for EV‐mediated transfer by employing a *Nrl.Cre^+/−^* and *mTmG^floxed^* reporter retinal co‐culture system (Fig 1H–J) followed by confocal imaging and flow cytometry analysis. Here, rod photoreceptors were enriched from *Nrl.Cre^+/−^* mice using the cell surface marker, CD73 (Eberle *et al*, 2011; Lakowski *et al*, 2015) and co‐cultured with mixed retinal cells from *mTmG^floxed^* mice a floxed Cre reporter line that ubiquitously expresses myristoylated RFP (myrRFP), but switches expression to myrGFP (“red‐to‐green”) upon acquisition of Cre and undergoing Cre‐mediated recombination (Appendix Fig S1). No spontaneous recombination was observed in untreated *mTmG^floxed^*‐only cultures, while *mTmG^floxed^* cultures treated with *AAV‐CMV.Cre* virus exhibited widespread recombination (Fig 1I and J). When *Nrl.Cre^+/−^* photoreceptors were co‐cultured above *mTmG^floxed^* cells and physical proximity between the two is prevented by use of a trans‐well (see schematic, Fig 1H, *left;* and (Zomer *et al*, 2016)), rare examples of recombination were seen after 21 days in culture (DIC) (Fig 1I and J). The majority of recombined cells were CD73^‐ve^ (Fig 1I), indicating a non‐photoreceptor identity (Eberle *et al*, 2011; Lakowski *et al*, 2011) and exhibited glial‐like morphologies (Fig 1J). Others have reported that EVs may get trapped within trans‐well pores (Thayanithy *et al*, 2017), but direct application of *Nrl.Cre^+/−^* EVs to *mTmG^floxed^* retinal cultures also yielded very low levels of recombination, again predominantly in CD73^−ve^ cells (Fig 1I). However, when cells were grown in physical contact with one another (see schematic, Fig 1H, right), significantly higher levels of recombination were observed. Some of these cells presented neuronal‐like morphologies (Fig 1J, right) and included CD73^+ve^ photoreceptors (Fig 1I). Together, these data indicate that cultured photoreceptors release bioactive EVs that are taken up predominantly by non‐photoreceptor populations, at least *in vitro*.

**Figure 2:**
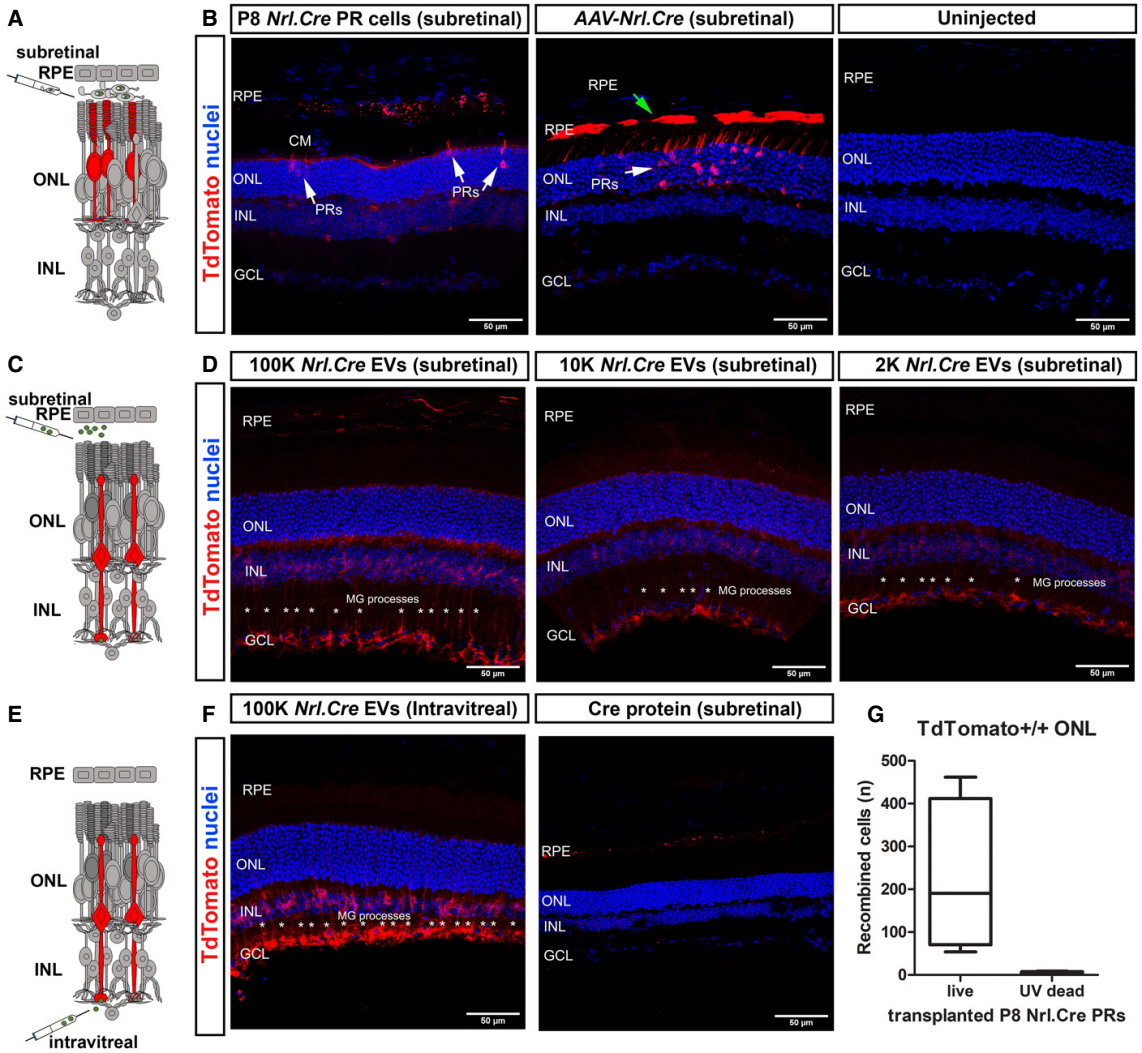
*Nrl.Cre^+/−^* photoreceptor‐derived EVs are taken up by Müller glia cells when injected *in vivo* ASchematic representation of photoreceptor transplantation, shown in (B).B(*left*) P8 *Nrl.Cre^+/−^* photoreceptors transplanted into subretinal space of *TdTomato^floxed^* reporter mice (*N* = 5 eyes), compared to (*middle*) subretinal injection of *AAV‐Nrl.Cre* subretinal injection (10^12^ vp/ml), (*N* = 10 eyes) and (*right*) contralateral uninjected control (*N* = 10 eyes). Arrows indicate recombined host photoreceptors (white arrows) or RPE (green arrows). Note clearly recombined RPE cells upon AAV transduction.CSchematic representation of subretinal EV injection, shown in (D). Red indicates host cells undergoing Cre‐mediated recombination and expressing TdTomato (*red*).DP8 *Nrl.Cre^+/−^* PR ‐derived (*lef*t) 100 K, (*middle*) 10 K and (*right*) 2 K EV pellets injected into the subretinal space of *TdTomato^floxed^* reporter mice (*N* = 4 eyes per EV preparation). Recombination was restricted to Müller Glia (asterisks).ESchematic representation of intravitreal EV injection, shown in (F).F(*left*) intravitreal injection of P8 *Nrl.Cre^+/−^* photoreceptor‐derived 100 K EV pellets (*N* = 2 eyes). Recombination was restricted to Müller Glia (asterisks) with little or no recombination in either photoreceptors or the RPE; (*right*) subretinal injection of recombinant Cre protein (control) into *TdTomato^floxed^* reporter mice. *Blue* = nuclei, *red* = TdTomato^+ve^ recombined cells. *N* = 4 retinae per group. Representative MIP confocal images.GQuantification of no. of TdTomato^+ve^ host photoreceptor cells seen following transplantation of live (224 ± 181) versus UV‐treated (dead) (3 ± 4) *Nrl.Cre^+/−^* P8 photoreceptors (*N* = 4 eyes per condition). Graph shows mean ± SD. Data information: Scale bars = 50 µm; PR—photoreceptor; MG—Müller Glia; RPE—retinal pigment epithelium; ONL—outer nuclear layer; INL—inner nuclear layer; GCL—Ganglion cell layer.

**Figure EV2:**
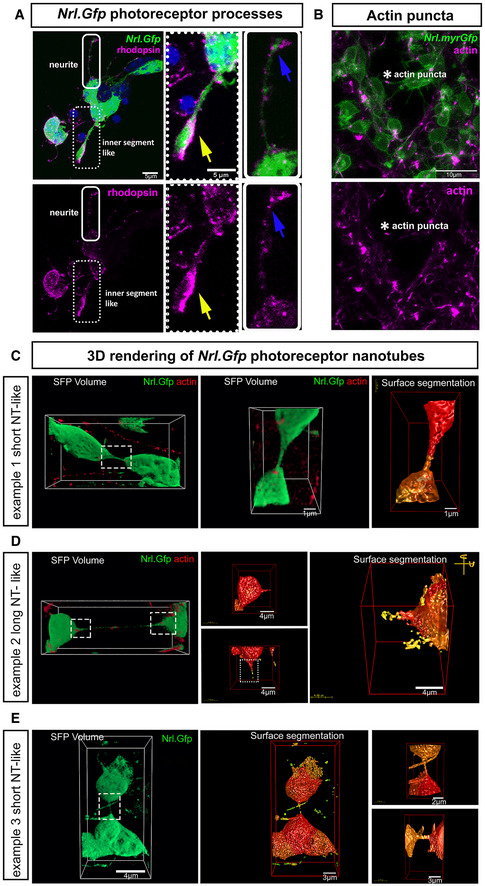
Photoreceptors form nanotube‐like processes in culture ARepresentative MIP example of rhodopsin immunostaining of fixed *Nrl.Gfp^+/+^* photoreceptors at 6 DIC. Digitally enhanced microphotographs of a neurite (*solid line rectangle*) and an inner segment‐like process (*dotted rectangle*), showing strong rhodopsin staining in the bulbous inner segment‐like structure (*yellow arrow*), compared to more dispersed staining in a neurite (*blue arrows*). Green = *myrGFP*, Magenta = Rhodopsin; Scale bar = 5 µm.BRepresentative MIP example of live imaging of P8 *Nrl.Cre^+/−^* × *mTmG* ^+/+^ (*myrGFP*) photoreceptor processes. SiR‐actin (*magenta*) reveals the presence of actin puncta (*white asterisk*) at the point of contact between a nanotube‐like process (herein termed ^Ph^NTs) and an adjacent photoreceptor. *Green* = *myrGFP*, *Magenta* = SiR‐actin; Scale bar = 10 µm.C–ERepresentative 3D deconvolved images of ^Ph^NTs from *Nrl.Gfp^+/+^* (*green*) P8 photoreceptor live‐imaged at DIV1–3 and labelled with SiR‐actin (*red*). (C) (*left*) Simulated Fluorescence Process (SFP) image of an example of a short ^Ph^NT and (*middle, right*) digitally enhanced 3D rendered surface per volume images of the same ^Ph^NT in rotation; Scale bar = 1 µm; (D) (*left*) SFP image of an example of a long ^Ph^NT and (*middle, right*) digitally enhanced 3D rendered surface per volume images of the same ^Ph^NT in rotation. Representative images from *N* = 7 independent cultures. Scale bar = 4 µm; (E) (*left*) SFP and (*middle, right*) digitally enhanced 3D rendered surface per volume images of the ^Ph^NT shown in Fig 3J. Scale bar = 4 µm; 3 µm; 2 µm; 3 µm, respectively, from left to right.

To explore this *in vivo*, *Nrl.Cre^+/−^* photoreceptor‐derived EVs were injected into the subretinal or intravitreal space of *TdTomato^floxed^* reporter mice (“no reporter‐to‐red” following Cre‐mediated recombination) and compared to subretinal transplantation of *Nrl.Cre^+/−^* photoreceptors or injection of either recombinant Cre or *AAV‐Nrl.Cre* virus (Fig 2). As we have reported previously (Pearson *et al*, 2016), transplantation of viable *Cre^+ve^* photoreceptors in the subretinal space results in recombination of host photoreceptors (Fig 2A and B). In notable contrast, when 100 K *Nrl.Cre^+/−^* photoreceptor‐derived EVs were injected into either the subretinal space (Figs 2C and D, and EV1, 2) or intravitreally (Figs 2E and F, and EV1D) we observed striking levels of recombination but only in Müller glia cells (Fig 2D and F). Injection of the 10 K and 2 K fractions, which may additionally contain microvesicles and/or apoptotic bodies (Thery *et al*, 2006, 2018), also yielded Müller glia‐specific labelling (Fig 2D) throughout the injection site. No other cell types were labelled. Importantly, transplantation of *Nrl.Cre^+/−^* photoreceptors that were pre‐treated with UV light to induce cell death yielded no recombination (Figs 2G and EV1E), nor did subretinal injection of recombinant Cre protein (2 μg; Fig 2F, right) or non‐photoreceptor‐derived EVs (Fig EV1D). Transduction with *AAV‐Nrl.Cre* virus yielded widespread recombination in the photoreceptors and some labelling of RPE cells (Fig 2B), most likely due to non‐specific expression of Cre within the RPE, or possibly recombination resulting from uptake of small amounts of Cre protein. Taken together, these data demonstrate that photoreceptor cells have the capacity to release EVs, at least in culture, and if present in the intact retina, these are specifically taken up by Müller glial cells. However, EVs do not mediate material transfer between photoreceptors.

### Photoreceptors form NT‐like processes *in vitro*

**Figure 3:**
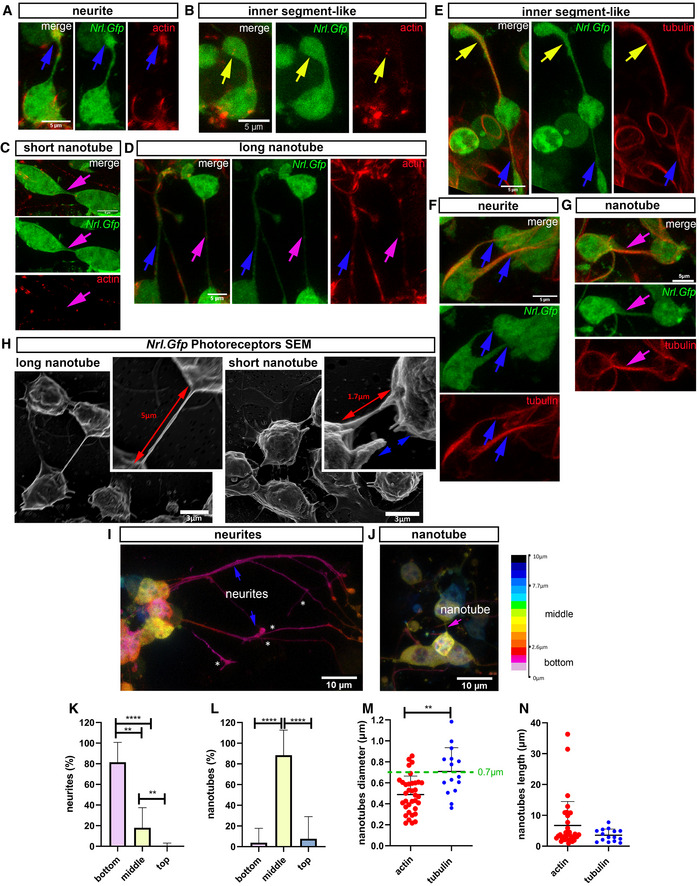
Photoreceptors form nanotube‐like processes to connect with neighbouring photoreceptors in culture A–DRepresentative 3D deconvolved MIP images from live imaging of *Nrl.Gfp^+/+^* (*green*) P8 photoreceptors at DIV1–3, labelled with SiR‐actin (*red*) (*N* = 7 independent cultures). Photoreceptors exhibit a variety of processes, including (A) neurites (*blue arrow*), (B) nascent inner segment‐like protrusions (*yellow arrow*), and (C) short and (D) long (*magenta arrows*) actin^+ve^ nanotube‐like (herein termed ^Ph^NTs) connections; Scale bar = 5 µm.E–GRepresentative 3D deconvolved MIP images from live imaging of *Nrl.Gfp^+/+^* (*green*) P8 photoreceptors at DIV1–3 labelled with SiR‐tubulin (*red*) (*N* = 7 independent cultures). Photoreceptors exhibit a variety of processes, including nascent inner segment‐like protrusions (*yellow arrows*), neurites (*blue arrows*) and rare tubulin^+ve Ph^NTs (*magenta arrows*); Scale bar = 5 µm.HRepresentative SEM images with digitally enhanced microphotographs of cultured P8 *Nrl.Gfp^+/+^* photoreceptors. *Red arrows* indicate ^Ph^NT connections between neighbouring photoreceptors, while *blue arrows* indicate broken ^Ph^NT connections (*N* = 3 cultures). Scale bar = 5 µm.I, JRepresentative images of depth colour coding (ImageJ) of x,y,z live imaging of *Nrl.Gfp^+/+^* photoreceptor cultures showing (I) neurites (*blue arrow*s) growing along the substrate (asterisks denote secondary branching of long GFP^+^ neurites), and in (J), a free‐floating ^Ph^NT (*magenta arrow*). Scale bar = 10 µm.K, LQuantification of the localization of *Nrl.Gfp^+/+^* processes in the z‐axis (depth) (*N* = 4 independent cultures, *n* = 1,096 cells, *n*_neurites_ = 440, *n*_nanotubes_ = 31) where (K) neurites. *P*‐values = ****P < 0.0001 (bottom vs top); **P = 0.002 (bottom vs middle); **P = 0.003 (middle vs top) and (L) ^Ph^NTs; *P*‐values = ****P < 0.0001 (bottom vs middle & middle vs top). One‐way ANOVA, non‐parametric, Kruskal–Wallis with Dunns’ multiple comparisons test shows means vary significantly.M, NDiameter and length quantification of SiR‐actin^+ve Ph^NTs (*N* = 7 cultures; *n*_cells_ = 646 cells; *n*_nanotubes_ = 35, *red dots*) compared to SiR‐tubulin^+ve Ph^NTs (*N* = 7 cultures; *n*_cells_ = 885 cells, *n*_nanotubes_ = 15; *blue dots*). One‐way ANOVA, non‐parametric, Kolmogorov–Smirnov test shows non‐significant (ns) difference in length but significant difference in diameter between actin^+ve^ versus tubulin^+ve Ph^NTs. *P*‐values; **P = 0.001, and *P* = 0.245, in (M) and (N), respectively. Data information: Graphs show mean ± SD.

In the original descriptions of material transfer in photoreceptor transplantation, donor cells were often, but not always, in close physical proximity with acceptor host cells (Pearson *et al*, 2016; Santos‐Ferreira *et al*, 2016; Singh *et al*, 2016), sometimes apparently extending processes towards the host acceptor cells (Singh *et al*, 2016; Ortin‐Martinez *et al*, 2017). We therefore considered whether physical cell–cell connections underlie photoreceptor material transfer (Fig 3). First, we performed confocal live imaging of *Nrl.Gfp^+/+^* photoreceptor cultures labelled with the cytoskeletal probes SiR‐actin or SiR‐tubulin, to explore and characterize the different processes extended by photoreceptors (Fig 3A–G; Appendix Table S1; Fig EV2); these include (i) neurite‐like extensions, (ii) nascent inner segment‐like processes, and (iii) nanotubes. Analysis of the actin cytoskeleton revealed neurite‐like extensions that terminate in actin‐rich exploratory growth cones (Fig 3A), as well as thicker processes ending in bulbous inner segment‐like terminals, which could be distinguished by the additional presence of tubulin (Fig 3B and E; Movie EV6). These inner segment‐like processes often robustly expressed the photopigment Rhodopsin (Fig EV2A). Examining SiR‐tubulin, many tubulin‐rich processes were long and thick and often extended around other cells (Fig 3F). However, they only rarely appeared to form cell‐to‐cell connections (Fig 3G). Strikingly, however, using either probe, we also observed distinctive NT‐like processes that formed continuous structures connecting the somas of pairs of adjacent cells (Fig 3C, D and G; Movies EV1–EV3). These NTs were typically short (< 10 μm), although longer lengths were observed (Fig 3C, D and G) and often remarkably straight (Fig 3H). Membrane continuity between the connected cells was further confirmed by scanning electron microscopy of *Nrl.Gfp^+/+^* photoreceptor cultures (Fig 3H). Live imaging showed that NTs extend freely between cells and are not attached to the substratum, in contrast with neurite‐like processes, which were usually attached to the substratum or other cells (Fig 3I–L); this free‐floating property also makes them sensitive to fixation (Fig 3H, right).

We were able to further classify photoreceptor NTs into Type I and Type II, based on their morphological appearance and molecular structure (Onfelt *et al*, 2006). Both types are usually short in length (< 10 μm) (Fig 3C and J) although some extend many tens of microns (Fig 3D and N). Type I are actin‐rich (Figs 3C, D, and J, and EV2C–E), the actin typically observed as plaques or puncta at the interface between soma and process, and along the process itself (Figs 3D and EV2B). In photoreceptor cultures, type I processes are typically very straight (Fig 3D) and < 0.7 μm diameter (Fig 3M). Type II processes additionally contain tubulin (Fig 3G) and are of thicker diameter (> 0.7 μm; Fig 3M). Neither type exhibited secondary branching in culture. At present, there is no single or panel of molecules that serve as definitive markers of NT‐like processes, but the above morphological properties of both types are consistent with published descriptions of NT‐like structures in other cell types (Korenkova *et al*, 2020). Both Type I and Type II NT‐like structures are relatively rare, comprising 5.4% and 1.7%, respectively, of all photoreceptor processes seen in culture. Herein, we refer to all photoreceptor–photoreceptor NT‐like processes as ^Ph^NTs.

### Photoreceptor NT‐like processes (^Ph^NTs) mediate material transfer *in vitro*

**Figure 4:**
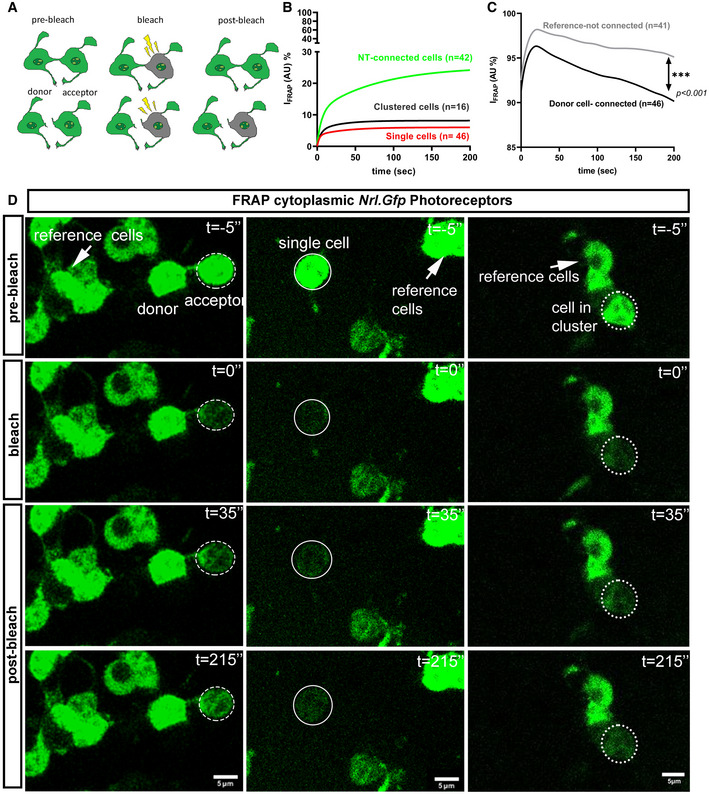
^Ph^NTs facilitate recovery after photobleaching in culture ASchematic representation of cytoplasmic (c)GFP mobility during fluorescence recovery after photobleaching (FRAP).BFRAP recovery curves for ^Ph^NT‐connected cells (*green*, *n* = 42, *R* ^2^ = 0.99), clustered cells (*black*, *n* = 16, *R* ^2^ = 0.94) and single cells (*red, n* = 46, *R* ^2^ = 0.97); Mann–Whitney analysis of fit for curves.CFRAP recovery curves in unbleached reference cells (*black*, *n* = 41) versus “donor” ^Ph^NT‐connected photoreceptor cells (*purple*, *n* = 46) show significant slope difference. Mann–Whitney analysis of fit for curves, ***P < 0.001.DRepresentative time‐lapse images of _c_GFP mobility between two ^Ph^NT‐connected *Nrl.Gfp^+/+^* photoreceptors (dashed ellipse = bleached area) versus an isolated cell (solid ellipse = bleached area) and clustered cells (dot ellipse = bleached area). Images show pre‐bleach (*t* = −5’’), bleach (*t* = 0’’) and post‐bleach (*t* = 35’’ and 215’’). Unbleached reference cells also indicated (arrows); Green = cytoplasm; Scale bar = 5 µm.

To assess the potential for cytoplasmic exchange of molecular information via ^Ph^NTs, pairs of ^Ph^NTs‐connected *Nrl.Gfp^+/+^* photoreceptors were examined using an adapted *fluorescence recovery after photobleaching* (FRAP) protocol (Fig 4A) and compared to isolated photoreceptors or those in clusters (but without apparent cell–cell connections; see Appendix Table S1 for criteria used to pre‐define connected cells prior to FRAP). As expected, isolated cells and cells within a cluster exhibited only limited recovery of cGFP (cytoplasmic GFP), reaching a maximum of ˜ 5% and ˜ 8% recovery of pre‐bleaching fluorescence levels, respectively (Fig 4B–D) with recovery kinetics indicating the redistribution of small amounts of cGFP throughout the cell (*n*_SINGLE_ = 46 cells, ti_1/2 fast_ = 1.87 s, ti_1/2 slow_ = 24.84 s; *n*_CLUSTERED_ = 16 cells, ti_1/2 fast_ = 3.18 s, ti_1/2 slow_ = 19.32 s). Conversely, in cells that exhibited a ^Ph^NT‐like connection with an adjacent “donor” cell (^Ph^NT‐connected cells; Movie EV4), we observed a slow but much larger recovery, achieving ˜ 25% of pre‐bleach fluorescence levels by the end of the imaging period (225 s) without having reached plateau (Fig 4B and C; *n*_NT_ = 42 cells, ti_1/2 fast_ = 4.19 s ti_1/2 slow_ = 54.86 s). Notably, this was accompanied by a concomitant reduction in cGFP fluorescence in the non‐bleached donor cell (Fig 4D), indicating that these cells are connected via an open‐ended cytoplasmic bridge.

**Figure 5:**
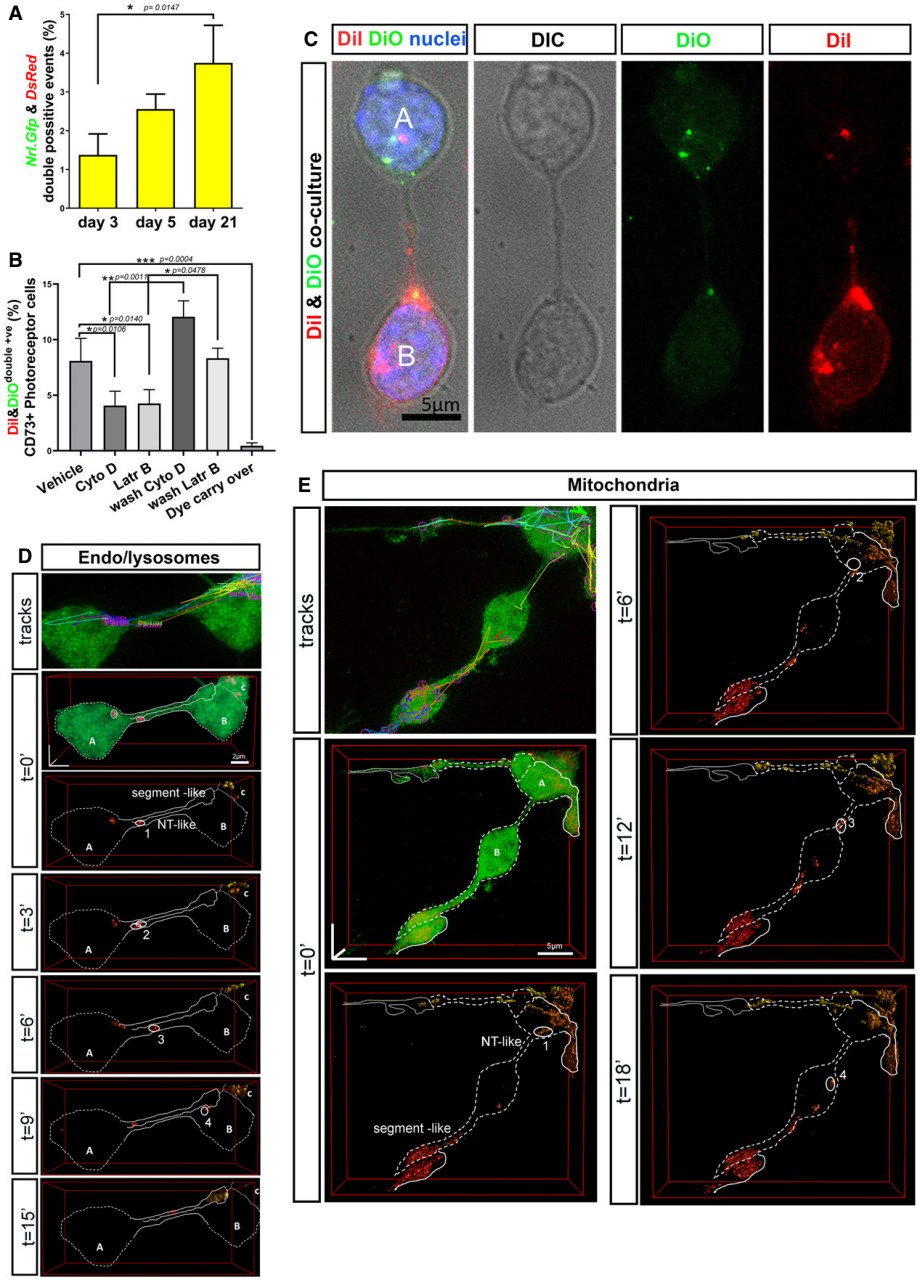
^Ph^NTs enable the intercellular transfer of cytoplasmic and membrane reporters and, rarely, organelles, in culture AFlow cytometry analysis of cGFP and cDsRed following co‐culture of *Nrl.Gfp* and *DsRed* cells (1:1) and analysed at 3 DIC (*N* = 3 independent co‐cultures with a technical triplicate), 7 DIC (*N* = 5), 21 DIC (*N* = 4); one‐way ANOVA non‐parametric, Kruskal–Wallis.BFlow cytometry analysis of DiI/DiO dual labelling of CD73^+^ cells following co‐culture of DiI‐ and DiO‐labelled photoreceptors (1:1) with pharmacological interventions applied at 5 DIC for 48 h, including vehicle control (DMSO), cytochalasin D (2 μM) and Latrunculin B (5 μM), after wash‐off of Cyto D and LatrB, and a final wash control for DiI/DiO carry over; *N* = 4 independent co‐cultures per condition; one‐way ANOVA non‐parametric, *F*‐test, Kolmogorov–Smirnov.CRepresentative MIP of DiI/DiO co‐cultures after fixation, showing DiO^+ve^ cell (A) and a DiI^+ve^ cell (B) cell connected by a ^Ph^NT. Note DiI and DiO puncta in the respective acceptor cells. *Blue* = Dapi (nuclei), *Green* = DiO, *Red* = DiI. Scale bar = 5 µm.DLysosomes can be exchanged, rarely, between ^Ph^NT‐connected cells. Lysosomes were labelled with SiR‐Lyso, and their movements were analysed by TrackMate software. Images show deconvolution of 3D reconstructions of lysosomes (surface; *red*) and cGFP (volume; *green*) from time‐lapse live imaging series of *Nrl.Gfp^+/+^* photoreceptor cultures (*green*); the position of a transferred lysosome is marked as1 (*t* = 0), 2 (*t* = 3’), 3 (*t* = 6’), 4 (*t* = 9’); N.B. Deconvolution shows a segment‐like process extending from cell “A” and a ^Ph^NT connecting cells “A” and “B”; Scale bar = 2 µm.EMitochondria can be exchanged, rarely, between ^Ph^NT‐connected cells. Mitochondria movements determined by TrackMate software. Images show deconvolution of 3D reconstructions of mitochondria (surface; *red*) and cGFP(volume; *green*) from time‐lapse live imaging series; position of transferred mitochondrion is marked as 1 (*t* = 0), 2 (*t* = 6’), 3 (*t* = 12’), 4 (*t* = 18’); Mitochondria labelled with mito‐Tracker‐Orange; Scale bar = 5 µm. Data information: Graphs show mean ± SD.

**Figure EV3:**
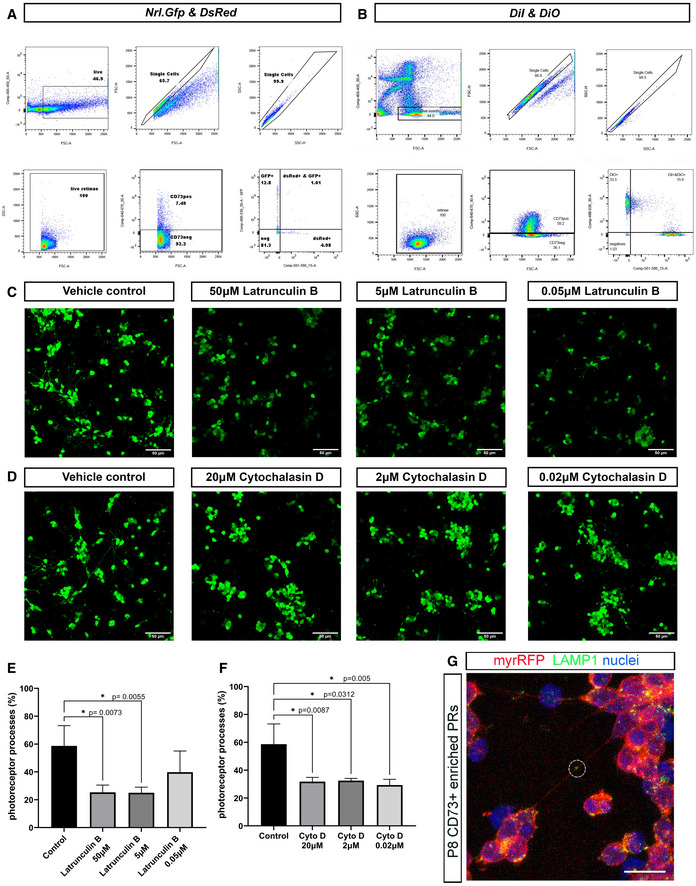
Pharmacological disruption of actin dynamics affects photoreceptor process formation and transfer of cytoplasmic and lipid reporters between photoreceptors in culture A, BFlow cytometry gating strategy of either (A) co‐cultures of *Nrl.Gfp* and *DsRed* cells, or (B) DiI and DiO labelled cells. In each case, panels reading *top left* to *bottom right* are: 1. Live cells selection, 2. Single events in FSC, 3. More single events in SSC, 4. Single live events in FSC/SSC, 5. CD73^−^APC versus FSC, 6. *Nrl.Gfp^+^* vs *DsRed^+^* within the CD73APC^+^ population (for A) or 6. DiI^+^ vs DiO^+^ within the CD73APC^+^ population (for B).C, DRepresentative examples of confocal imaging of P8 *Nrl.Gfp^+/+^* photoreceptors fixed after 48 h exposure to different concentrations of the actin polymerization inhibitors (C), Latrunculin B (50 µM, 5 µM and 0.05 µM) and (D), Cytochalasin D (20 µM, 2 µM and 0.02 µM), compared to vehicle controls (DMSO); Scale bars = 50 µm.E, FStatistical analysis of the percentage of photoreceptors exhibiting processes following 48 h treatment with Latrunculin B (50 µM, 5 µM and 0.05 µM) or Cytochalasin D (20 µM, 2 µM and 0.02 µM), compared to vehicle control. One‐way ANOVA non‐parametric, two‐tailed analysis, *F*‐test, Kolmogorov–Smirnov, *P < 0.05 shows significant effect of both drugs on the ability of photoreceptors to elaborate processes, compared to vehicle control; (*N* = 3 independent cultures, *n* = 1 well per condition). Graph shows mean ± SD.GRepresentative MIP of myrRFP^+ve^/CD73^+ve^ photoreceptors (*red*) immunostained for LAMP1 (*green*) after 3DIC; *Circle* = LAMP1^+ve^ vesicle in long ^Ph^NTs. Scale bar = 10 µm.

We next sought to determine the levels of cytoplasmic fluorescent reporter exchange occurring spontaneously between cells in cocultures of sorted *Nrl.Gfp* ^+/+^ and *DsRed* ^+/+^ photoreceptors. As assessed by flow cytometry (Figs 5A and EV3A), a proportion of cells exhibited detectable levels of both labels (˜ 4% at 21DIV, increasing over time; Fig 5A), consistent with previous observations regarding NT‐like protrusions in other cell types (Rustom *et al*, 2004). Together, these results indicate that fluorescent cytoplasmic molecules can be exchanged between ^Ph^NT‐connected photoreceptor cells *in vitro*, but that this is likely to be a transient process occurring in a relative minority of cells at any given point in time, consistent with material transfer events occurring after photoreceptor transplantation (Pearson *et al*, 2016).

Next, we examined membrane exchange by co‐culture of wildtype photoreceptors pre‐labelled with either DiI or DiO membrane dyes (Figs 5B and C, and EV3B). Flow cytometry analysis showed that under control conditions (5DIC plus 48 h with DMSO vehicle control) ˜ 8% of rod photoreceptors were double labelled (Fig 5B), indicating lipid exchange. Of note, the fluorescence shift indicated that cells from each starting population acquired relatively small amounts of the other fluorescent label, rather than extensive membrane exchange (Fig EV3B). This was confirmed by live imaging, which showed pairs of ^Ph^NT‐connected photoreceptors, each containing puncta of the other label (Fig 5C). Puncta of both labels were also visible along the connecting process.

To examine the involvement of actin in ^Ph^NT formation and transfer, we combined DiI/DiO co‐culture with application of either Cytocholasin D (2 μM) or Latrunculin B (5 μM), which each prevent actin polymerization. Appropriate doses were selected based on the literature (Onfelt *et al*, 2006; Gurke *et al*, 2008; Bukoreshtliev *et al*, 2009) and a dose–response‐based assessment of process formation (all types) (Fig EV3C–F). The selected doses significantly impaired the formation of photoreceptor processes but did not affect cell viability. Each drug was therefore applied to DiI/DiO co‐cultures for 48 h at 5DIC; both inhibitors significantly reduced DiI/DiO exchange on photoreceptor cultures in a reversable manner (Fig 5B). Interestingly, DiI/DiO transfer was decreased by ˜ 2‐fold in the presence of actin inhibitors, which is similar to the observed ˜ 2‐fold reduction in ^Ph^NT formation.

We also assessed whether larger structures like lysosomes and mitochondria can be exchanged between ^Ph^NT‐connected photoreceptors. LAMP1^+ve^ endosomal vesicles were observed within ^Ph^NT processes connecting neighbouring photoreceptors in fixed cultures (Fig EV3G). Moreover, live imaging of *Nrl.Gfp^+/+^* photoreceptors labelled with the lysosomal label, SiR‐lyso (Fig 5D; Movie EV5) or mitochondrial label, Mito‐tracker Orange (Fig 5E; Movie EV6) showed rapid movements of lysosome/late endosomes and mitochondria within the cytoplasm and within a variety of processes, including segment‐like processes and ^Ph^NTs. Object tracking software and careful analysis of deconvolved images revealed examples where lysosomes or mitochondria were clearly transferred from one cell to another via ^Ph^NTs, although these events were relatively rare (1 and 2 confirmed exchange events, respectively, observed from imaging 50 pairs of ^Ph^NT‐connected cells, each pair imaged for ˜ 60 min; Fig 5D and E).

### ^Ph^NT‐like processes facilitate material transfer *in vivo*

**Figure 6:**
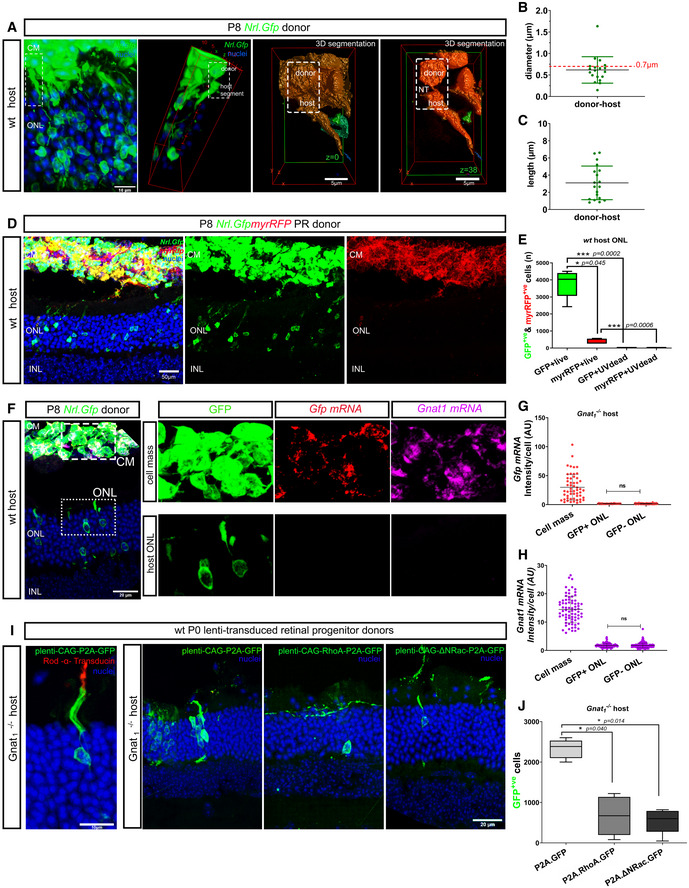
Transplanted photoreceptors form ^Ph^NT processes with host photoreceptors and transfer cytoplasmic and membrane proteins but transfer of mRNA was not detected AMIP image of wild‐type (wt) retina transplanted with P8 *Nrl.Gfp^+/+^* CD73^+^ MACS‐enriched photoreceptors; *dashed box* indicates ROI subjected to 3D deconvolution and volume reconstruction (*right*) and shows ^Ph^NT‐like connection between donor and host photoreceptor at the level of host inner segment. Additional images show 3D segmentation of *z* = 0 and *z* = 38 focal planes, and reveal the ^Ph^NT; *green* = GFP, *blue* = nuclei; Scale bar = 10 µm in MIP and 5 µm volume reconstruction.B, CAssessment of properties of ^Ph^NT‐like processes between donor and host photoreceptors (*n* = 19 ^Ph^NT‐like processes; *N* = 10 eyes) showing (B) diameter and (C) length.DMIP image of *wt* retina transplanted with P8 *Nrl.Gfp^+/+^ x myrRFP^/+^* photoreceptors; *showing wt* host ONL photoreceptor labelled with cGFP (*green*) but not with myrRFP (*red*); Scale bar = 50 µm.EQuantification and statistical analysis of material transfer events following subretinal transplantation of live (cGFP = 3,775 ± 804, myrRFP = 436 ± 145) or UV‐treated (cGFP = 1.3 ± 2.5, myrRFP = 4.3 ± 5.7) *Nrl.Gfp^+/+^x myr‐Rfp* ^+/+^ photoreceptors into wt hosts (*N* = 8 and *N* = 4 retinae, respectively); one‐way ANOVA, parametric, two‐tail, Tukey’s multiple comparisons; graph shows mean ± SD.F–HAssessment of potential for transfer of donor *Gfp* and *Gnat1* mRNA during transplantation using RNAscope™. (F) Representative MIP images of *Gnat1* ^−/−^ eyes transplanted with *Nrl.Gfp^+/+^* donor cells and processed *in situ* for *Gfp* mRNA (*red*) and *Gnat1* mRNA (*magenta*); *blue* = nuclei; ROIs indicated in (F) show GFP^+ve^ donor cell mass (CM) and GFP^+ve^ cells within *Gnat1* ^−/−^ host outer nuclear layer (ONL). G, H Quantification of *Gfp* and *Gnat1* mRNA (mean fluorescence intensity per cell), respectively (*N* = 3 retinae); one‐way ANOVA, non‐parametric, two‐tailed analysis showed no significant differences in signal intensity for either *Gnat1* or *Gfp* mRNA between GFP^+ve^ and GFP^−ve^ host photoreceptors.IManipulation of actin signalling alters material transfer *in vivo:* (*far left*) MIP image showing GFP^+ve^ host photoreceptor (*green*) expressing Rod α‐transducin (*red*) after transplantation of retinal cells transduced with *lenti‐CAG‐P2A.Gfp* (transduction control) into *Gnat1* ^−/−^ recipient; scale bar = 10 µm; adjacent images show *Gnat1* ^−/−^ eyes transplanted with P2 retinal cells transduced with (*left*) *plenti‐CAG‐P2A.GFP* (*green*), or (*middle*) *plenti‐CAG‐RhoA‐P2A.GFP* (*green*), or (*right*) *plenti‐CAG‐ΔΝRac1‐P2A.GFP (green)*; *green* = GFP, *blue* = nuclei; Scale bar = 20 µm.JQuantification of the number of GFP^+^ cells in *Gnat1* ^−/−^ host ONL, (*N* = 5 retinae per condition); *plenti‐CAG‐P2A.GFP* (2,328 ± 234), *plenti‐CAG‐RhoA‐P2A.GFP* (668 ± 481), *plenti‐CAG‐ΔΝRac1‐P2A.GFP* (547 ± 306); one‐way ANOVA, non‐parametric, Dunn’s multiple comparisons test. Graph shows mean ± SD. Data information: CM = cell mass; all eyes were processed 3 weeks post‐transplantation. RPE—Retinal Pigment Epithelium; ONL—outer nuclear layer; INL—inner nuclear layer; GCL—Ganglion Cell Layer. Graphs show mean ± SD.

**Figure EV4:**
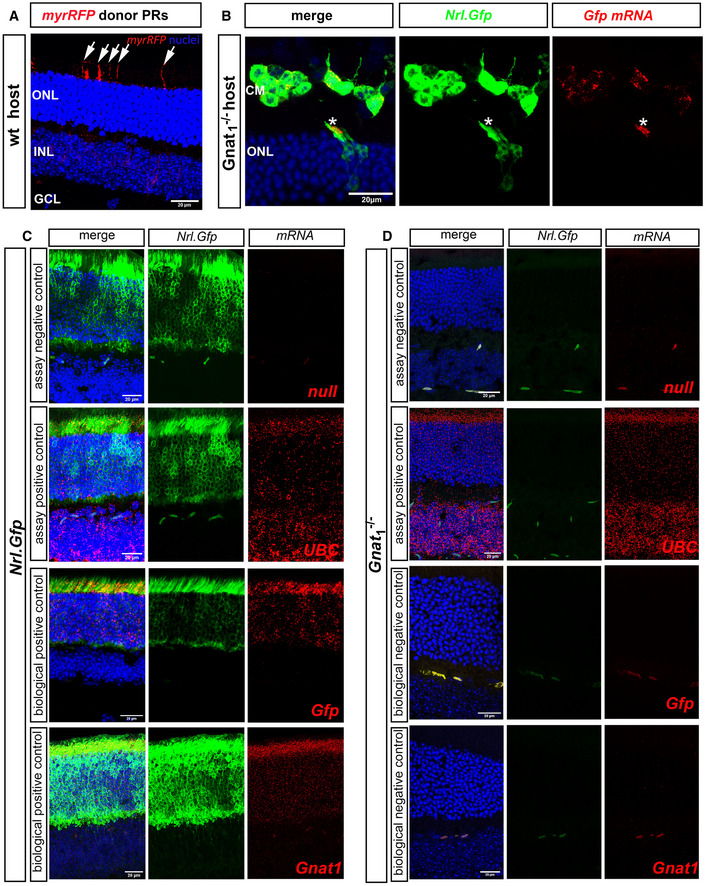
Transplantation of *Nrl.Gfp^+/+^* photoreceptors results in the transfer of cytoplasmic protein and some membrane‐bound protein, but little or no mRNA ARepresentative MIP confocal images of *wild‐type* (wt) eye cups fixed 21 days post‐transplantation with P8 myrRFP^+ve^ CD73^+ve^ MACS‐enriched photoreceptors. Note the presence of RFP^+^ host inner segments (*arrows*). myr‐RFP (*red*), nuclei (*blue*).BRepresentative MIP confocal images of *Gnat1* ^−/−^ eye cups fixed and stained in situ (RNAScope) with *Gfp* mRNA probe (*red*) 21 days post‐transplantation with P8 *Nrl.Gfp^+/+^* photoreceptors (*green*). *Blue* = Dapi (nuclei); CM: cell mass; ONL: Outer Nuclear Layer. Asterisks denote a rare example of an integrated donor photoreceptor located within the host ONL and presenting robust staining for *Gnat1* mRNA in the inner segment.C, DRepresentative MIP confocal images of (C), *Nrl.Gfp^+/+^* and (D), *Gnat1* ^−/−^ eye cups fixed and stained *in situ* (RNAScope) with a null probe (assay negative control, *red*), *UBC* mRNA probe (assay positive control, *red*), *Gfp* mRNA probe (biological positive control, *red*), *Gnat1* mRNA probe (biological positive control, *red*), and *Gnat1* ^−/−^ tissue stained with either *Gfp* mRNA probe (biological negative control, *red*) or *Gnat1* mRNA probe (biological negative control, *red*). *Nrl.Gfp^+/+^* photoreceptors (*green*), nuclei (*blue*); mRNA (*red*). Data information: Scale bars = 20 µm.

The existence of ^Ph^NT‐like processes capable of transferring membrane lipids, proteins and potentially even organelles between photoreceptors in culture is striking and we sought to determine whether similar structures exist and function *in vivo*. In the first instance, we examined interactions between transplanted donor and host photoreceptors. The outer segments of host photoreceptors project towards the RPE and those of GFP^+^ host acceptor cells are thus in very close proximity to the donor cell mass (Fig 6A). This is to be expected but makes it difficult to visualize donor–host connections using standard imaging. However, careful analysis of deconvolved confocal images of host wild‐type retinae following transplantation of *Nrl.Gfp* ^+/+^ donor photoreceptors revealed, alongside with the expected presence of larger neurites, GFP^+ Ph^NT‐like processes extending between individual donor–host pairs (Fig 6A; Movie EV7). Sensitivity to fixation and tissue processing constraints meant that verified examples were seen relatively infrequently but those observed were very thin (Fig 6B) and lacked secondary branching. They were also typically < 10 μm in length (Fig 6C), but we note that longer connections may be more prone to disruption by fixation and sectioning. We also transplanted donor cells derived from *Nrl.Gfp^+/+^ x myrRfp^+/+^* mice, in which GFP^+^ rods additionally express membrane‐tagged RFP (myrRFP). While the transfer of cGFP was similar to that reported previously (Pearson *et al*, 2016; Santos‐Ferreira *et al*, 2016; Singh *et al*, 2016), fewer examples of myrRFP transfer were found, despite very robust fluorescence being observed in the donor cell mass (Fig 6D and E). RFP fluorescence was predominantly observed in the inner segments of host cells (Fig EV4A). This may reflect a more spatially limited exchange of membrane‐bound molecules, as was also observed for DiI/DiO exchange in photoreceptor cultures, and by others (Rustom *et al*, 2004). However, detection of myrRFP transfer may also be reduced due to its slightly weaker fluorescence, compared to cGFP. Regardless, transfer of both membrane and cytoplasmic reporters requires healthy viable donor cells and does not arise from uptake of debris; no transfer was seen in any host photoreceptors following transplantation of UV‐treated donors, at either 72 h (*N* = 4) or 21 days (*N* = 6) post‐transplantation (Fig 6E and Appendix Fig S2A and B).

We have previously shown that transplantation of *Nrl.Gfp* ^+/+^ photoreceptors into the *Gnat1* ^−/−^ model of stationary night blindness leads to the presence of both GFP and rod α‐transducin (*Gnat1*) in host photoreceptors (Pearson *et al*, 2012; Warre‐Cornish *et al*, 2014). The efficiency of transfer appears similar for both molecules as rod α‐transducin is found in > 95% of GFP^+^ host acceptor cells (Warre‐Cornish *et al*, 2014; Pearson *et al*, 2016) and the extent of transfer is sufficient to restore visual function (Pearson *et al*, 2012, 2016). Whether this rescue occurs by the transfer of RNA and/or protein is not yet known but is important for our understanding of material transfer in the transplantation setting and any potential role ^Ph^NT‐like extensions might play in intercellular communication more broadly. To examine this, we transplanted *Nrl.Gfp^+/+^* photoreceptors into *Gnat1* ^−/−^ hosts and employed RNA in situ hybridization (ISH), using RNAscope™, to label either *Gfp* or *Gnat1* mRNA (Figs 6F–H, and EV4B–D). RNAscope uses an RNA ISH method, where single‐molecule visualization in individual cells is achieved through use of a novel probe design strategy and a hybridization‐based signal amplification system to simultaneously amplify signals and suppresses background. As expected, *Nrl.Gfp^+/+^* retinae show robust labelling in the ONL with both *Gfp* and *Gnat1* probes, while the same probes yielded no labelling in *Gnat1* ^−/−^ retinae (Fig EV4C and D). Similarly, after transplantation, *Nrl*.*Gfp^+/+^* donor cells in the subretinal space of *Gnat1* ^−/−^ recipients showed robust labelling for both probes (Fig 6F). Conversely, while numerous GFP^+^ acceptor cells were present within the host ONL, little or no labelling for either *Gfp* (Fig 6F and G) or *Gnat1* (Fig 6F and H). Rarely (2/246 GFP^+^ host cells), we observed GFP^+^ cells in the host ONL that showed positive labelling for Gfp mRNA (Fig EV4B). Both cells were within the first 1–2 rows of the host ONL and their nuclei exhibited a multi‐chromocenter pattern of staining, consistent with that of immature rods (Solovei *et al*, 2009), or cones. These therefore likely represent rare true integration events, as we have described previously (Pearson *et al*, 2016). Thus, we suggest that material transfer between donor and host is predominantly mediated as protein, consistent with the rapid exchange of cGFP observed *in vitro* (Fig 4A). It also supports the notion that such transfer is transient, limited by the half‐life of the protein in question and sustained by the continued presence of, and physical interaction with, donor cells, as we had previously predicted (Pearson *et al*, 2016).

**Figure EV5:**
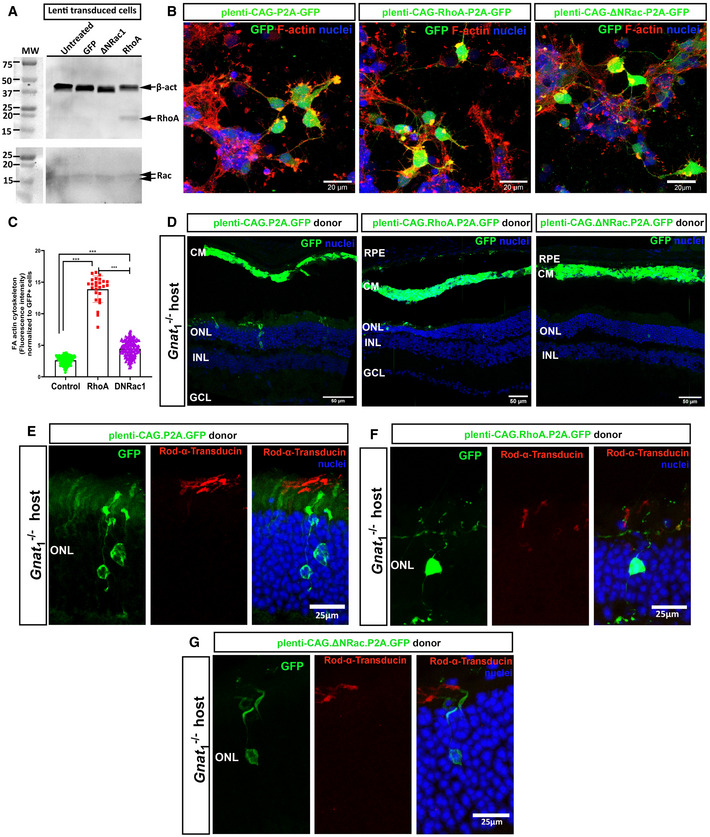
Manipulation of actin dynamics by overexpression of RhoA or DNRac1 in transplanted photoreceptors correlates with reduced GFP and rod α‐transducin transfer ARepresentative Western blots of *RhoA and Rac1* expression in mixed P0–2 retinal cultures transduced with plenti‐CAG‐P2A.GFP, plenti‐CAG‐ΔΝRac1‐P2A.GFP or plenti‐CAG‐RhoA‐P2A.GFP compared to β‐actin and assessed after 8 DIC.BRepresentative MIP confocal images of P0–2 retinal cultures transduced with plenti‐CAG‐P2A.GFP (*green*) or plenti‐CAG‐RhoA‐P2A.GFP (*green*) or plenti‐CAG‐ΔΝRac1‐P2A.GFP (*green*), fixed and stained with F‐actin (*red*) (*N* = 2 independent cultures, *n* = 4 wells per condition); Scale bars = 20 µm.CQuantification and statistical analysis of the effect of overexpression of RhoA and ΔΝRac1, versus control, on actin in P0–2 retinal cultures at 7DIC. Effect assessed by measurement of mean intensities (Image J) of F‐actin plaques (*red*) in GFP^+^ cells (*green*), normalized to GFP^+^ cell number; control 2.6 ± 0.7; RhoA 13.9 ± 2.1, ΔΝRac 4.5 ± 1.3. Mean ± SD. One‐way ANOVA non‐parametric two‐tail, Kruskal–Wallis post‐test ***P < 0.001 (*N* = 2 independent cultures, *n* = 4 wells per condition).DRepresentative tile‐scan images of *Gnat1* ^−/−^ eyes transplanted with P2 retinal cells transduced with *plenti‐CAG‐P2A.GFP* (*green*) or *plenti‐CAG‐RhoA‐P2A.GFP* (*green*) or *plenti‐CAG‐ΔΝRac1‐P2A.GFP (green)*. *Green* = GFP, *blue* = Dapi (nuclei); Scale bars = 50 µm.E–GRepresentative MIP images of *Gnat1* ^−/−^ eyes transplanted with P2 retinal cells transduced with (E), *plenti‐CAG‐P2A.GFP* (*green*) or (F), *plenti‐CAG‐RhoA‐P2A.GFP* (*green*) or (G), *plenti‐CAG‐ΔΝRac1‐P2A.GFP (green)*. Immunostaining for Rod α‐transducin indicates that inhibition of actin polymerization impairs transfer of Rod α‐transducin alongside that of GFP. *Green* = GFP, *blue* = Dapi (nuclei), *red* = Rod α‐transducin; Scale bars = 50 µm. Data information: RPE = retinal pigment epithelium, CM = cell mass, ONL = outer nuclear layer, INL = inner nuclear layer, GCL = ganglion cell layer. All eyes were fixed and examined 21 days post‐transplantation.

We next sought to manipulate actin dynamics in donor cells *in vivo* to examine whether this impeded cGFP transfer, like the effects of pharmacological inhibition of actin in cultures. Rho GTPases are important for actin stress fibre formation and actomyosin contractility and the activity of RhoA is incompatible with membrane protrusion (Raftopoulou & Hall, 2004). Conversely, Rac1 activity is required for membrane protrusion at lamellipodia tips (Mehidi *et al*, 2019) and, of relevance here, has been linked to a fundamental role in the biogenesis of NTs (Hanna *et al*, 2017). Wild‐type P0–2 cells were transduced with lentivirus over‐expressing RhoA (*RhoA.GFP*) or a dominant negative form of Rac1 (*DNRac1.GFP*) or empty vector expressing only GFP (*Ctrl.GFP*). Expression was confirmed by GFP fluorescence, Western blotting and F‐actin staining (Fig EV5A–C). Donor cells were transplanted into *Gnat1* ^−/−^ adult eyes and examined 3 weeks later. Similarly sized, large cell masses were seen in the subretinal space of all retinae transplanted with *RhoA.GFP^+^, DNRac1.GFP^+^* or *Ctrl.GFP* donor cells (Fig EV5D). However, both displayed significantly reduced transfer of cGFP (Fig 6I and J) and rod α‐transducin (Fig EV5E–G) to host acceptor photoreceptors, indicating that actin polymerization is required for material transfer and the concomitant gain of function (rod‐α‐transducin), *in vivo*.

### Evidence of material transfer between photoreceptors in the normal retina

**Figure 7:**
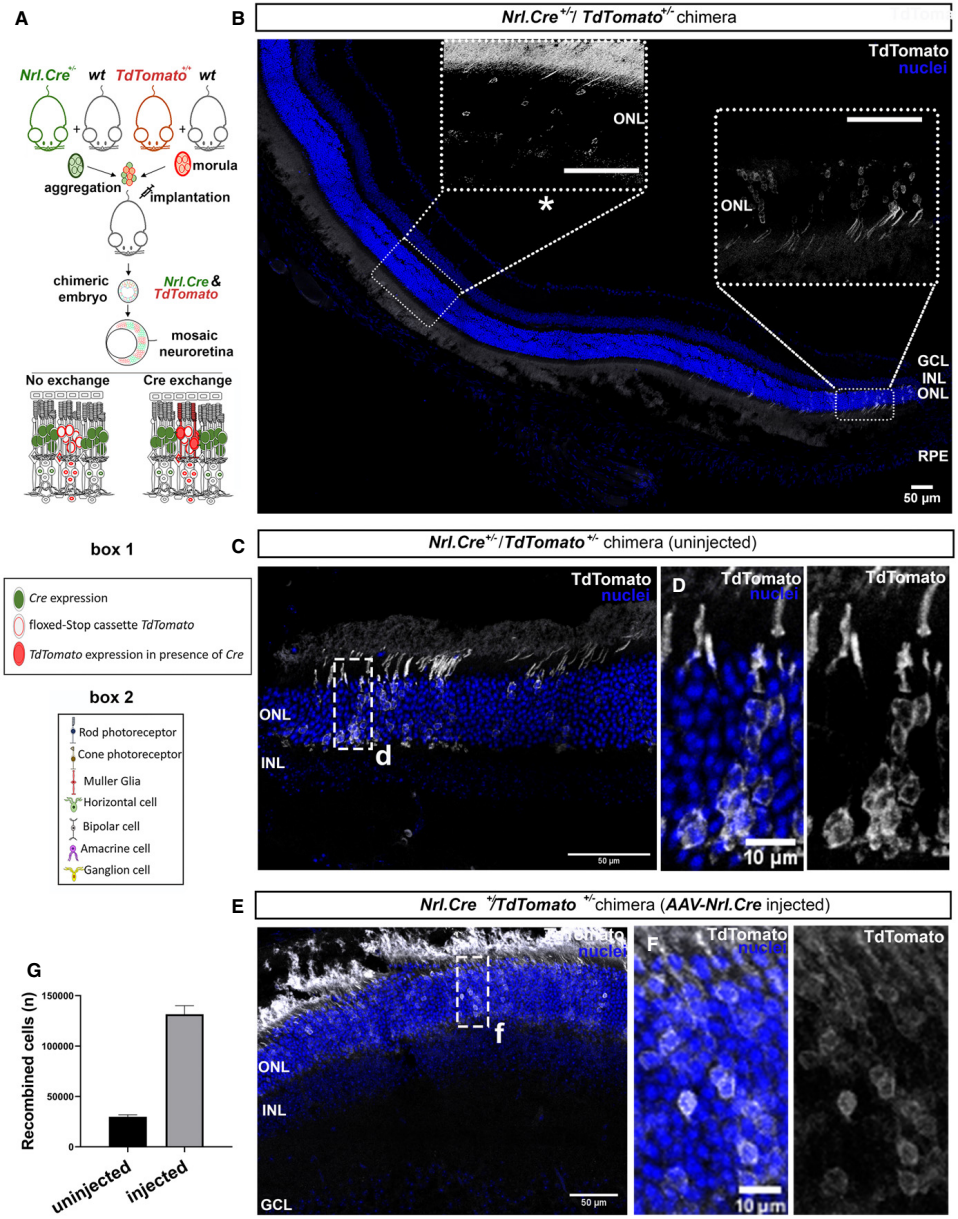
Generation of *Nrl.Cre^+/−^/TdTomato^floxed^* aggregate chimeras reveals intercellular exchange of Cre between photoreceptors *in vivo* ASchematic representation of *Nrl.Cre^+/−^/TdTomato^+/−^* floxed chimera generation via morula aggregation, which results in chimeric mosaicism in the neural retina. Recombination and expression of TdTomato is only possible if *Nrl.Cre^+/−^* photoreceptors exchange material (Cre) with *TdTomato^floxed^* cells via intercellular communication.BRepresentative tile scan of uninjected *Nrl.Cre^+/−^*/*TdTomato^+/−^* chimera showing endogenous recombination (*white*); ROIs, as indicated with dashed or dot boxes; Scale bars = 50 µm.C, DROI from uninjected *Nrl.Cre^+/−^*/*TdTomato^+/−^* chimera showing spontaneous recombination presenting in mosaic stripe patterning; Scale bar = 50 µm in (C) and 10 µm in (D).E, FRepresentative images of *Nrl.Cre^+/−^*/*TdTomato^+/−^* chimera after receiving a subretinal injection of *AAV‐Nrl.Cre*. The injected eyes show widespread recombination, which also shows the anticipated mosaic stripe patterning; Scale bar = 50 µm in (E) and 10 µm in (F).GQuantification of number of recombined cells in uninjected (29,825 cells ± 2,031) and *AAV‐Nrl.Cre‐*injected (131,562 ± 8,478) *Nrl‐Cre^+/−^*/*TdTomato^+/−^* chimeras, compared with uninjected *Nrl‐Cre* ^−/−^ *(wt)*/*TdTomato^+/−^* chimera (0 ± 0). Graph shows mean ± SD. Data information: *white* = TdTomato^+ve^ recombined cells; *blue* = nuclei. RPE‐ Retinal Pigment Epithelium; ONL—outer nuclear layer; INL—inner nuclear layer; GCL—Ganglion Cell Layer.

Lastly, since transplantation involves a degree of stress to both donor and host cells, we asked whether material transfer between photoreceptors occurs in the normal mammalian retina. For this purpose, chimeric embryos were generated by aggregation of *Nrl.Cre^+/−^* (in which expression of Cre is tightly restricted to rod photoreceptors; Appendix Fig S1A and B) and *TdTomato^+/−^* morulae. In *Nrl.Cre^+/−^/TdTomato^+/−^* chimeras, rod photoreceptors will carry either *Cre* or *TdTomato^floxed^*, but not both; only *TdTomato^+/−^* cells acquiring Cre from neighbouring Cre^+^ rods could potentially express TdTomato and recombination is not possible in any other chimera outcome (Fig 7A). Strikingly, we observed many TdTomato^+^ photoreceptors—and only photoreceptors—in *Nrl.Cre^+/−^/TdTomato^+/−^* adult chimera (Fig 7B–D). These cells bore all the morphological characteristics of rod photoreceptors and only ever exhibited a single nucleus that displayed a single chromocenter, typical of rods. They presented both as single cells (Fig 7B, left dashed box) and more typically as small clusters (Fig 7B, right dashed box, C and D) and were most numerous towards the peripheral retina. Conversely, no spontaneous recombination was seen in any control *TdTomato^+/−^* eyes (*n* = 20). Administration of AAV‐*Nrl.Cre* virus, in which expression of *Cre* is under the control of the *Nrl* promoter, was used to confirm photoreceptor‐specific recombination of the *Nrl.Cre^+/−^/TdTomato^+/−^* genotype (Fig 7E and F; see also Appendix Fig S1B). To our knowledge, this is the first demonstration of material transfer between photoreceptors in the intact adult neuroretina *in vivo*.

## Discussion

Neuronal communication is mediated by chemical and electrical synapses and by paracrine signals, such as neurotrophic factors and neuropeptides. Increasingly, however, other mechanisms including EVs, TNTs and other NT‐like processes are being proposed to play important roles in neuro‐glial networks in processes as diverse as synaptic pruning and neurodegeneration (Agnati & Fuxe, 2014). There is mounting evidence to suggest that EVs might mediate neuron–neuron and neuron‐glia signalling, but evidence of the existence of NT‐like structures in primary mammalian neurons is very limited, and whether they form *in vivo* has not been shown.

During our investigations into photoreceptor transplantation, we, and others, discovered the unusual phenomenon of material transfer (Pearson *et al*, 2016; Santos‐Ferreira *et al*, 2016; Singh *et al*, 2016; Decembrini *et al*, 2017; Ortin‐Martinez *et al*, 2017), where transplanted donor photoreceptors exchange a wide range of molecules, both endogenous and transgenic, with photoreceptors in the host retina. Where the host acceptor cells are diseased, this process can be sufficient to restore function (MacLaren *et al*, 2006; Lamba *et al*, 2009; Pearson *et al*, 2012). Here, using aggregate chimeras, we report the important further discovery that material transfer may also occur between photoreceptors in the intact retina.

In the first instance, we considered EVs as the most likely mediators of material transfer between photoreceptors, since they fulfil many of the characteristics of transfer seen both in chimeric retinae and in transplantation. We found that postnatal rod photoreceptors form MVBs, which are necessary for EV maturation, *in vivo* and can release EVs, at least in culture. Photoreceptor‐derived EVs display a molecular signature specific to photoreceptors, but distinct from the cytoplasmic contents of the same cells. These EVs contained either protein or mRNA for components of the phototransduction machinery, amongst others, but not the transcription factor, *Crx,* in contrast to recent reports (Zhou *et al*, 2018). Interestingly, some molecules were present as mRNA while others were detectable as protein. This is consistent with other reports of EV‐derived cargo (Jeppesen *et al*, 2019; Murillo *et al*, 2019) although it remains to be determined why these differences arise. One potential explanation may be that membrane‐bound and cytosolic proteins might become incorporated within an EV in different forms (i.e. as mRNA or as protein) but this is beyond the scope of the current study.

Perhaps surprisingly, photoreceptor‐derived EVs are not taken up by other photoreceptors either in culture or following injection into the adult but are instead taken up specifically by Müller Glia. Elsewhere, EV‐mediated neuron‐glia communication has been linked to synaptic activity and plasticity (Chivet *et al*, 2014; Bahrini *et al*, 2015; Ashley *et al*, 2018; Pastuzyn *et al*, 2018) and myelination (Fruhbeis *et al*, 2013). It is perhaps surprising therefore that we did not see recombination in Müller Glia in *Nrl.Cre^+/−^/TdTomato^+/−^* chimeric mice, indicating that, if photoreceptors do indeed release EVs in the normal retina, they do not represent a significant mode of neuron‐glia communication in the healthy retina. An alternative explanation may be that EVs are indeed released by photoreceptors *in vivo,* but they are immediately cleared by local immune cells under normal conditions, as recently shown in zebrafish (Verweij *et al*, 2019). Indeed, other studies indicate that EVs are involved in various stages of the injury process, including propagating inflammation, mediating neuroprotection (Sun *et al*, 2020), and modulating regeneration (Peng *et al*, 2018). As such, they are proposed to play active roles in the pathogenesis of several neurodegenerative diseases (Hill, 2019). It will therefore be of significant future interest to determine what (if any) role photoreceptor‐derived EVs play in neuron‐glia signalling in retinal degeneration.

Material transfer between photoreceptors instead appears to be mediated by physical connections in the form of ^Ph^NT processes. TNTs and NT‐like processes have been regarded with scepticism by some parts of the scientific community (Ariazi *et al*, 2017; Baker, 2017) as there is relatively limited information regarding their structural identity and if, or how, they differ both amongst each other and from other cellular protrusions, such as filopodia. Nonetheless, in the chick embryo NT‐like processes can form between migrating neural crest cells and mediate directional changes in following cells (Teddy & Kulesa, 2004). Perhaps the most compelling evidence for a functional role *in vivo* comes from *Drosophila*, where cytonemes are responsible for the distribution of morphogens, including Wnt and Hedgehog (Hg), in the development of the wing imaginal disk and eye disk (Roy *et al*, 2011; Bischoff *et al*, 2013; Stanganello *et al*, 2015). To date, the only evidence that such structures exist in mammalian tissues *in vivo* is between immune cells in the mouse cornea (Chinnery *et al*, 2008) and, recently, between pericytes (Alarcon‐Martinez *et al*, 2020).

Here, we show that, at least in culture, photoreceptors can form ^Ph^NT structures that fall into one of two groups, Type I (thin, typically straight, actin‐rich) or Type II (thicker, containing both actin and microtubules) processes, with Type I being more numerous; both were distinct from exploratory neurite‐like processes. These characteristics are consistent with the descriptions of TNTs between macrophages in culture (Onfelt *et al*, 2006) and differ from the purely actin‐containing TNTs described in PC12 (Rustom *et al*, 2004) and CAD (Gousset *et al*, 2009; Vargas *et al*, 2019) cells. Earlier studies indicated that actin‐only and actin/microtubule‐containing structures may support unidirectional (Onfelt *et al*, 2006; Arkwright *et al*, 2010) and bidirectional (Rustom *et al*, 2004; Gousset *et al*, 2009; Domhan *et al*, 2011) transfer, respectively. Moreover, a recent elegant fluorescent EM analysis of NTs formed by CAD cells *in vitro* showed bundles of individual actin‐rich NTs facilitating the movement of vesicles and organelles along their length using motor proteins such as Myo10 (Sartori‐Rupp *et al*, 2019). Regardless of type, the biosynthesis of NT‐like protrusions is attributed to the activity of Rac1 and Cdc42 (Hanna *et al*, 2017) and F‐actin polymerization (Onfelt *et al*, 2006; Gurke *et al*, 2008; Bukoreshtliev *et al*, 2009; Ljubojevic *et al*, 2021). These pathways obviously perform many different functions within the cell, but in keeping with a role in NT formation, we found that F‐actin depolymerizing drugs significantly impaired material transfer between photoreceptors in culture, while a dominant negative form of Rac1 and overexpression of RhoA each significantly impaired the transfer of the cytoplasmic fluorescent reporter, GFP, and the phototransduction protein, rod α‐transducin, from GFP^+^ wildtype donors to dysfunctional host photoreceptors *in vivo,* following transplantation.

Precisely what is exchanged via NT‐like processes is likely determined by the cells in question and the type of processes formed. Some include gap junction proteins and permit the propagation of electrical and calcium signals, but may exclude proteins of molecular weights > 1 kD, like GFP (Watkins & Salter, 2005; Hase *et al*, 2009; Wang *et al*, 2012b; Alarcon‐Martinez *et al*, 2020). Paradoxically, open‐ended TNTs formed by PC12 cells are reported to permit the exchange of small diameter organelles but not the passive diffusion of cGFP (Rustom *et al*, 2004). Conversely, cytonemes are thought to be close‐end tubes and can establish signalling gradients by releasing molecules, such as Hg, packaged in EVs, from their terminal tips (Gradilla *et al*, 2014; Chen *et al*, 2017). Here, we found that cytoplasmic proteins can be exchanged between connected photoreceptors with surprising speed and efficacy, as assessed by FRAP in culture and in transplantation *in vivo*, while membrane labels were exchanged but to a more limited extent. Organelles (lysosomes, mitochondria) were also observed to pass, at least in culture, albeit rarely. We suggest that organelle exchange may be mediated by Type II ^Ph^NTs, which are thicker than Type I and additionally contain tubulin and may thus be able to facilitate large cargo transfer. Type II ^Ph^NTs were rarely present in culture, so the chances of directly observing organelle transfer may be correspondingly limited. Type I ^Ph^NTs were more numerous, and we consider these to be the most likely mediators of protein transfer between photoreceptors, both in culture and in transplantation.

Along with proteins and organelles, we also addressed the potential for mRNA transfer. Despite the robust presence of proteins such as cGFP and rod‐α‐transducin in acceptor cells, at least in transplantation, mRNA of these molecules was not observed using a sensitive RNA in situ hybridization technique (RNAscope™). We cannot exclude the possibility of small amounts of mRNA transferring between connected cells, as reported recently between stem cells in culture (Haimovich & Gerst, 2019). Together, the above indicate that material transfer between photoreceptors can occur via two types of open‐end ^Ph^NTs. These thus appear distinct from the recently reported closed‐end pericyte NTs that regulate neurovascular coupling in the retina (Alarcon‐Martinez *et al*, 2020). Our data show that, at least for cytoplasmic molecules, the transfer mechanism involves an actin‐dependent network, both in culture and *in vivo*, in line with *in vitro* reports by others for other cell types (Rustom *et al*, 2004; Hanna *et al*, 2017). Elucidating the sorting mechanisms that determine what cargo can/cannot be exchanged and how this relates to different types of NT‐like processes will be of major importance as we explore their role in tissue homeostasis.

Perhaps one of the most striking findings of our investigations was the demonstration of material transfer between photoreceptors in the intact chimeric retina. Further work is required to conclusively show that this is mediated by ^Ph^NTs but it nonetheless raises fundamental questions regarding the role of material transfer in retinal function, and how this might sit within the framework of intercellular communication more broadly. Here, we show that ^Ph^NT‐like processes mediate the transfer of molecules that, at least within the transplantation paradigm, have been shown to support gain of function in the receiving cell. Rod α‐transducin can be transferred from transplanted donor cells to non‐functional *Gnat1* ^−/−^ host photoreceptors in sufficient quantities to render those cells light responsive (Pearson *et al*, 2012, 2016). This raises the possibility that ^Ph^NT‐like structures may be important for maintaining function under stress and/or dysfunction. Consistent with this notion, transplanted hematopoietic progenitor stem cells appeared to partially rescue the corneal defect seen in cystinosin knockout (*Ctns* ^−/−^) mice by local, unidirectional transfer of cystinosin, from healthy donors to *Ctns* ^−/−^ macrophages *in vivo* (Rocca *et al*, 2015). Indeed, the role of NT‐like processes in disease and tissue homeostasis is still unclear as, at least in culture, the transfer of organelles such as mitochondria and lysosomes has been reported to occur both to and from the diseased/dying cells (Spees *et al*, 2006; Wang *et al*, 2011; Rocca *et al*, 2015; Wang & Gerdes, 2015; Rustom, 2016). In our hands, material transfer occurs between apparently healthy neurons *in vitro*, in transplantation and in the intact retina, and has the potential to bring about gain‐of‐function where the acceptor cell is dysfunctional.

In summary, we show that photoreceptors can form open‐ended membranous NTs that mediate the transfer of cytoplasmic and lipid‐bound material to other photoreceptors, both *in vitro* and in transplantation. Of note, a similar set of observations have been described in a parallel study conducted by Ortín‐Martínez *et al* (2021); Wallace and colleagues also show that material transfer between photoreceptors is mediated via membranous processes whose formation is regulated via Rho‐GTPases. These processes, which they also term photoreceptor nanotubes (^Ph^NTs), facilitate the transfer of fluorescent reporter molecules *in culture* and in transplantation. Moreover, we further show, for the first time, that material transfer can occur between photoreceptors in the intact retina. Both studies provide new insights into the mechanisms underlying transplant‐mediated cellular repair in the nervous system and raise important questions about the potential roles played by NTs and material transfer in both the healthy nervous system and in disease.

## Materials and Methods

### Reagents and Tools table

**Table:** 

Reagent/Resource	Reference or source	Identifier or catalog number
**Experimental Models**		
C57BL/6J (*M. musculus*)	Jackson Lab	B6.129P2Gpr37tm1Dgen/J
Nrl.gfp (*M. musculus*)	Kind gift of Dr Anand Swaroop, now available through Jackson Lab	B6. Cg‐Tg(Nrl‐EGFP)1Asw/J
Ai9(RCL‐tdT) or TdTomato (M. *musculus*)	Jackson Lab	B6. Cg‐Gt(ROSA)26Sortm9(CAG‐tdTomato)Hze/J
mTmG or myrRFP (*M. musculus*)	Jackson Lab	Gt(ROSA)26Sortm4(ACTB‐tdTomato,‐EGFP)Luo/J
Gnat1^−/−^ (*M. musculus*)	kind gift of Dr Janis Lem, Tufts Medical College	Gnat1^−/−^; Gnat1KO
Nrl.Cre (*M. musculus*)	Jackson Lab	C57BL/6J‐Tg(Nrl‐cre)1Smgc/J
**Recombinant DNA**		
Pd10.Nrl.Cre	This paper, based on Akimoto *et al*, 2006 and Brightman *et al*, 2016 sequences	N/A
AAVPk	Addgene	N/A
pHGTI helper	Plasmid factory	PF1810
pLenti‐CAG‐GFP‐P2A	Generated by Dr Ortín‐Martínez Arturo and Professor Valerie Wallace, University of Toronto	N/A
pLenti‐CAG‐RhoA‐GFP‐P2A	Generated by Dr Ortín‐Martínez Arturo and Professor Valerie Wallace, University of Toronto	N/A
pLenti‐CAG‐ΔNRac‐GFP‐P2A	Generated by Dr Ortín‐Martínez Arturo and Professor Valerie Wallace, University of Toronto	N/A
pMD2.G	Addgene	N/A
pCMVΔR8.74	Addgene	N/A
**Antibodies**		
CD81	Cell Signalling	10037
TSG101	BD Transd	612696
CD9	EDM Millipore	CBL162
Alix	Cell Signalling	2171
Cre recomb	EDM Millipore	MAB3120
GM130	Cell Signalling	12480
Opsin	Sigma	O4886
G‐alpha‐t1	Santa Cruz INC	Sc389
Recoverin	MERCK	AB5585
GFP/CFP/RFP	Clontech	632380
Anti‐LAMP1	Abcam	[1D4B] (ab25245)
Beta actin	R&D Systems	MAB8929
Beta actin	Sigma	A2228
Rac	Cytoskeleton Inc	ARC03‐S
RhoA	Cell Signaling	(67B9) Rabbit mAb #2117
CD73 APC	Miltenyl	130‐103‐052
**Oligonucleotides and sequence‐based reagents**		
*rtQ*PCR primers	This study	Expanded View‐ Materials and Methods
Gfp: GAAGCGCGATGACATGGT/CCATGCCGAGAGTGATCC/ probe 67	This study	Expanded View‐ Materials and Methods
Cre: ATCTGGCATTTCTGGGGATTG/ GCAACACCATTTTTTCTGACCC/ probe 20	This study	Expanded View‐ Materials and Methods
Gnat1: AGAGCTGGAGAAGAAGCTGAAA/ TAGTGCTCTTCCCGGATTCA/ probe 89	This study	Expanded View‐ Materials and Methods
Rcvrn: CAATGGGACCATCAGCAAA/ CCTCAGGCTTGATCATTTTGA/ probe 67	This study	Expanded View‐ Materials and Methods
Rho: ACCTGGATCATGGCGTT/ TGCCCTCAGGGATGTACC/probe 32	This study	Expanded View‐ Materials and Methods
Crx: CCCCAATGTGGACCTGAT/GGCTCCTGGTGAATGTGGT/ probe 64	This study	Expanded View‐ Materials and Methods
Nrl; TTCTGGTTCTGACAGTGACTACG/ TGGGACTGAGCAGAGAGAGG/ probe 53	This study	Expanded View‐ Materials and Methods
Actb:AAGGCCAACCGTGAAAAGAT/GTGGTACGACCAGAGGCATAC/probe 56	This study	Expanded View‐ Materials and Methods
**Chemicals, enzymes and other reagents**		
RNase	Sigma	
Super RNAse Out 20 U/µl,	Thermo Fisher	
TaqMan^®^ Universal PCR Master Mix	Roche UK	
FAM RTQPCR probe	Universal Probe Library; Roche, Germany	
Mitotracker‐Orange	(Invitrogen/Molecular Probes, M7512)	
SiR‐actin	Cytoskeleton	
SiR‐tubulin	Cytoskeleton	
SiR700‐lysosome	Cytoskeleton	
verapamil	Cytoskeleton	
DiI or DiO lipophilic tracers	Thermo Fisher	
Cytochalasin D	Sigma	
ROCK inhibitor Y‐27632	Sigma	
Latrunculin B	Sigma	
Fibronectin	Sigma	
Poly‐D‐lysine hydrobromide	Sigma	
**Software**		
GraphPad Prism 8	GraphPad	
Adobe Photoshop version 22.4	Adobe	
Image J (FIJI)	NIH	
HyVolution software (Scientific Volume Imaging Huygens)	Scientific Volume Imaging	
LEICA LASX	Leica	
Chemidoc‐Gel Lab	Bio‐Rad	
Fast Real‐time Sequence Detection System (Applied Bioscience, USA)	Applied Bioscience	
**Other**		
QuantiTect^®^ Whole Transcriptome	Qiagen	
Qiagen RNeasy^®^ Mini kit	Qiagen	
Immobilon™ Western Chemiluminescent HRP substrate kit	Millipore, USA	
CD73 APC and anti APC microbeads and magnetic rack	Miltenyl	
Worthington Papain Dissociation System	Worthington Biochemical Corporation, USA	

### Methods and Protocols

#### Animals

Animal lines used: C57BI/6J (wildtype, wt) (Harlan), Nrl.Gfp^+/+^ (kind gift of A. Swaroop, University of Michigan, USA) (Akimoto *et al*, 2006), Ai9^(RCL‐tdT)^ or TdTomato^floxed^ (Madisen *et al*, 2010) Gt(ROSA)26Sor^tm4(ACTB‐tdTomato,‐EGFP)Luo^ or “mTmG” or “myrRFP” (Muzumdar *et al*, 2007) Gnat1^tm1Clma^ or Gnat1^−/−^ (kind gift of J. Lem, Tufts University School of Medicine, USA) (Calvert *et al*, 2000) and C57BL/6J‐Tg(Nrl‐cre)1Smgc/J or *Nrl.Cre^+/−^* (Brightman *et al*, 2016), except for *Nrl.Cre*, which was maintained as hemizygote, due to lethality issues. Mice were maintained in the animal facilities at University College London or King’s College London. All experiments have been conducted in accordance with the Policies on the Use of Animals and Humans in Neuroscience Research, revised and approved by the ARVO Statement for Use of Animals in the Ophthalmic Research, under the regulation of the UK Home Office Animals (Scientific Procedures) Act 1986. Briefly, rodents were maintained on a standard 12/12‐h light dark cycle, housed in same sex groups or sustained breeding pairs wherever possible and provided with fresh bedding and nesting material and food and water ad libitum.

#### Transplantation surgery

Transplantation was conducted as we have described previously (Pearson *et al*, 2012) All recipients were adult (6–8 weeks) at time of transplantation. Briefly, recipient mice were anaesthetized with a single intra‐peritoneal injection of a mixture of Dormitor (1 mg/ml medetomidine hydrochloride, Pfizer Pharmaceuticals, Kent, UK), ketamine (100 mg/ml, Fort Dodge Animal Health, Southampton, UK) and sterile water for injections in the ratio of 5:3:42. Prior to cell transplantation, the tip of 34‐G needle was inserted to the sclera and slowly removed. The needle with the cell suspension was then inserted tangentially in through the sclera and RPE, injecting slowly the 1 μl cell suspension, or 2 µl (2 μg) of concentrated EV fractions, or 2 μl (2 μg) of Cre protein (ab134845) underneath the RPE in the superior retina resulting in small retinal detachment.

#### Generation of Chimeras by morula aggregation

*De novo* chimeric mice were generated by mixing two genetic populations *Nrl.Cre* and the *TdTomato^floxed^* reporter lines, both of C57BI/6J background, by morula reaggregation. Briefly, 3–4 weeks old *wt* females were super ovulated with PMSG; 5 IU [International Unit] in 0.1 ml IP on day 1 followed by a second injection of hCG; 5 IU on day 3, after which they are immediately placed in the cage with the mutant male. Embryos were collected at the eight‐cell morula‐stage from the two genotypes by flushing oviducts at 2.5 dpc. All embryos were collected and washed with FHM medium and KSOM. The sandwich aggregation technique was performed by Dr Signore, UCL Institute of Child Health. Briefly, the removal of embryo’s zona pellucida was achieved using Tyrode’s acid solution and transferred to FHM medium, and finally, the zona‐free embryos were transferred back into KSOM. Once all the embryos are isolated, the embryos of the first genotype were placed individually into the indentations of the aggregation plate, and the process was repeated for the second genotype making sure that the embryos in each indentation are physically attached to each other. The aggregation plate was incubated for 19–24 h at 37°C/ 5% CO_2_. The next day, the aggregates should have formed a single embryo at the late morula or blastocyst stage. The embryos were implanted by microinjection in pseudo‐pregnant recipient females and the females checked for pregnancy several days post‐implantation. Chimeras were collected at P45.

#### Chimera genotyping

Genomic DNA was extracted from ear clips following manufacture’s protocol (Extracta™DNA Prep for PCR, QuantaBio), and 5 μl of extract was used per 50 μL PCR reaction. PCR reactions were performed following the manufacture’s protocol (Promega) following a cycle of: Denaturation (2 min 95°C), Reaction (30 s 95°C, 30 s 60°C, 45 s 72°C), and Final extension (5 min 72°C), with a final hold at 10°C.

Primers sequences: *Nrl.Cre*; 5'‐GCATTACCGGTCGATGCAACGAGTGATGAG‐3' and 5’GAGTGAACGAACCTGGTCGAAATCAGTGCG‐3’ with a product of 400 bps. *TdTomato* (*wt*); 5’‐AAGGGAGCTGCAGTGGAGTA‐3’ and 5’‐CCGAAAATCTGTGGGAAGTC‐3’ (mutant) 5’‐GGCATTAAAGCAGCGTATCC‐3’ and 5’‐CTGTTCCTGTACG GCATGG‐3’ with products; tdTomato^−/−^ 297 bp, tdTomato^−/+^ 196 bp 297 bp tdTomato^+/+^ 196 bp accordingly.

#### Tissue harvesting fixation and cryo‐sectioning

Animals were sacrificed by cervical dislocation. Eyes were removed, and the cornea punctured to improve the efficiency of the fixation. The eyes were fixed in 4% PFA (PBS) for 1 h at RT (conventional IHC), or 4% PFA (dH_2_O) 24 h (RNAScope), eye cups dissections performed, followed by cryopreservation with 20% sucrose (Sigma, USA) in PBS for 1 h at RT or for 24 h at 4°C (RNAScope). To preserve nanotube‐like structures *in vivo* punctured eyes were washed in 5% Sucrose and fixed with 7.5% sucrose, 2% PFA (dH_2_O) overnight in dark humid chambers at 4–8°C. Eyes embedded in OCT and rapidly frozen in liquid Nitrogen. The frozen samples were stored at −80°C until cryo‐sectioning on a Bright OTF5000 cryostat (Bright Instruments Co Ltd, UK) set at 12–17 μm thickness. Sections were serially transferred onto 2–10 sets of SuperFrost‐TM ultra Plus Adhesion glass slides (Thermo Fisher, USA) and stored at −20°C for staining, and at −80°C for RNAScope.

#### In situ *branched hybridization method (RNA Scope)*

Multi‐plex RNAscope assay was performed according to manufacturer’s protocol with sterile solutions and enzymes in a specific HybEZ™ Oven with EZ‐Batch™ Slide holder all provided by Advanced Cell Diagnostics (ACD) (Wang *et al*, 2012a). Briefly, samples were air‐dried, washed with PBS, baked for 30 min at 60°C and post‐fixed with 4% PFA 15 min at 4°C. Slides were then dehydrated through a series of Ethanol washes (50%, 70%, 100%), air‐dried and treated with Hydrogen Peroxidase for 10 min, washed at RT and subjected to antigen retrieval solution (ACD) for 3 min at 88°C, before immediately washing with dH_2_O, 100% Et‐OH, and air‐dried again prior to protease III treatment for 30 min at 40°C. The slides then were washed with dH_2_O and hybridized with RNAscope probe mix (*Gnat1* probe (524881‐C2) diluted 1:50 in *EGFP* probe (400281‐C1) solution; see ACD bank link for sequences: https://acdbio.com/catalog‐probes) for 3 h 40°C, before being washed with Wash Buffer (ACD) and stored overnight in 5XSSC solution at RT. Slides were then subjected in three subsequent series of amplification with Multiplex FL v2 Amp 1, Amp2 and Amp3 for 30 min at 40°C each and subsequent wash in wash buffer. For visualizing both EGFP_C1 and Gnat1_C2, HRP signal was developed in two steps for HRP_C1 and then HRP C2 for 15 min at 40°C followed by a 5 min wash in wash buffer. Fluorochromes were assigned by using Opal™ dyes (Akoya’s Opal™ Multiplex IHC kits diluted 1;1,500 in TSA probe diluent buffer, ACD) and incubation for 30 min at 40°C followed by a 5 min wash. Finally, HRP signal was blocked by incubation for 15 min at 40°C with Multiplex FL v2 HRP blocker (ACD) and slides washed with wash buffer and counterstained with DAPI and subjected to IHC for anti‐GFP immunostaining. Negative and positive multiplex control probes were provided by ACD and tested in parallel with main probes following either a single or multiplex protocol (shown in Fig EV4).

#### Immunohistochemistry (IHC) and Immunocytochemistry (ICC)

Tissue sections were air‐dried for 30 min, rehydrated with PBS and incubated in blocking buffer at RT for 1 h. ICC blocking buffer composed of 0.1% Saponin, 1% BSA and 2% NGS or the appropriate serum depending on the secondary used. IHC blocking buffer composed of 2% NGS, 1% BSA and 0.05–1% Triton X‐100. Primary antibodies used in the range of 1:100–1:1,000. Primary antibodies used; Anti‐LAMP1 (rat monoclonal, 1:500, [1D4B] (ab25245), Abcam), anti‐Gat_1_ (rabbit polyclonal, 1:1,000, K‐20, SC‐389 Santa Cruiz), anti‐Rhodopsin (mouse, 1:2,000, O4886, Sigma) and anti‐GFP, (goat polyclonal, 1:1,000, ab6673, Abcam). Sections were then incubated in primary antibody overnight at 4°C, washed with PBS and incubated with secondary antibody for 2 h at RT in blocking solution. Sections were then washed and counterstained with DAPI (4’,6‐Diamidino‐2‐Phenylindole, Dihydrochloride; 1:1,000 dilution in 1× PBS (Molecular ProbesTM, USA)). Subsequently, sections mounted under coverslips with fluorescence mounting medium (Dako, Denmark) and stored covered at 4°C to prevent exposure to light. Immunocytochemistry followed the same procedure as immunohistochemistry. Negative controls omitted the primary antibody.

#### Confocal microscopy of fixed tissue

Images of fixed tissue sections were acquired on a Leica TCS SPE upright confocal laser scanning microscope (Leica, Germany) that was configured to a scanning frequency of 400 Hz, fitted with 5× (air, numerical aperture or NA = 0.15), 40× (oil immersion, NA = 1.15) and 63× (oil immersion, NA = 1.3) objectives and photomultiplier tubes to detect fluorescence emission. DAPI was excited with a 405 nm laser source. The 488 nm and 532 nm laser sources were used for the fluorophores Alexa Fluor^®^ 488 and Alexa Fluor^®^ 546, respectively. When acquiring the fixed images, xyz confocal stacks of representative fields of view were captured at a resolution of 1,024 × 1,024 pixels at a step size of 1 μm for retinae tissue, 0.5 μm for cell cultures. To maintain consistency, tissue sections within a given experiment were imaged with the same laser power, detector gain and offset settings. Identical settings allowed for semi‐quantitative comparison of protein expression levels between developmental time points. LAS AF image browser software was used to compile and export the acquired images. Lif data files were then processed in Fiji/ImageJ (National Institutes of Health, USA) (Schindelin *et al*, 2012).

#### Primary photoreceptors, retinal cultures and cocultures

Retinal dissociation was performed as previously described (Pearson *et al*, 2012) with some modifications. Briefly, neuroretinas were removed and placed in fresh dissection medium were the neuroretina was removed (DMEM/F12 of PBS or HBSS or EBSS no phenol red (Invitrogen) supplemented with 15 mM HEPES, 1 mM Taurine and 0.5 U/ml DNAase) and then placed in prewarm papain/DNAase solution (˜ 100 μl/retinae) for dissociation using the Papain Dissociation System (Huettner & Baughman, 1986). The solution was then incubated for ˜ 20 min in a water bath at 37°C with discontinuous agitation, following centrifugation at 260 × *g* for 5 min at RT. The supernatant was discarded, and the pelleted cells were reconstituted with resuspension buffer (2.7 ml EBSS, 300 µls reconstituted albumin‐ovomucoid inhibitor, and 50 µl of DNase solution), 250 µl of resuspension buffer was used per 10 retinae and layered over 1 ml of albumin‐inhibitor solution in a centrifuge tube, to form a discontinuous gradient, then centrifuged at 70 × *g* for 10 min at RT. Dissociated retinae cells pellet at the bottom of the tube and transferred in chemically defined medium (CD), cell viability, assessed with Trypan blue and a ViCellXR Cell Counter and for retinae cultures cells plated in a final density not exceeding 70,000 cells/mm^2^ in 1 mg/ml PDL (Sigma) /0.04 mg/ml fibronectin (Sigma) pre‐coated glass bottom plates. For mixed population retinal cocultures, cells from two different genotypes were isolated in the same day and mixed in a ratio 1:1.

In the experiments involving primary photoreceptor cultures, photoreceptors were enriched using APC‐conjugated CD73 (Eberle *et al*, 2011; Lakowski *et al*, 2015), which immuno‐reacts with anti‐APC magnetic beads (Miltenyl) and sorted with a MACSQuant^®^ Analyzer (Miltenyl). Briefly, after dissociation, cells were resuspended into MACS buffer (Miltenyl) supplemented with 15 mM HEPES, 1 mM Taurine and 0.5 U/ml DNAase at a concentration of 10^7^ cells per 100 µl and Primary anti‐mouse‐CD73 APC‐conjugated antibody (Miltenyl) was added to the cells at a concentration of 1 μl Ab/10^6^ cells for 20–30 min at 4°C. Cells were then centrifuged for 10 min at 260 × *g* at 4°C, and the cell pellet was resuspended in anti‐APC solution (20 µl anti‐APC‐Microbeads and 80 µl MACS buffer) and incubated for 20 min at 4°C. The samples were subsequently loaded into the MACSQuant^®^ Analyzer, selecting the “Positive selection sensitive” programme for magnetic separation. CD73^+^APC Photoreceptor precursors are found in the second eluate, with the first (negative) eluate comprising the fraction of all other retinae cells. The positive fraction was centrifuged immediately at 160 × *g* for 20 min at 4°C. Viability was then assessed as for retinal cultures and photoreceptors were plated in a final density not exceeding 85,000 cells/mm^2^ in 1 mg/ml PDL (Sigma)/0.04 mg/ml fibronectin (Sigma) pre‐coated glass bottom plates.

Both photoreceptor and retinal primary cultures were maintained in CD medium; BrainPhys™ Neuronal Medium, (STEMCELL Technologies Inc) supplemented with 15 mM HEPES (Invitrogen), 0.01 mg/ml recombinant human insulin, 0.0055 mg/ml human transferrin (substantially iron‐free) and 0.005 μg/ml sodium selenite, 1 mM Taurine, 3% Tissue culture grade BSA solution (Sigma) and antibiotics Pen/ Strep 10 mg/ml (Sigma) and incubated at 37°C, 5% CO_2_. Medium was changed every 3–4 days.

For photoreceptor‐retinal cell trans‐well co‐cultures, a trans‐well insert system was used where retinal cells were plated in the bottom of a 24‐well plate pre‐coated with PDL at a density of 3–4 × 10^5^ cells per well. The retinae cells that plated at the bottom of the dish were allowed to recover for 8–19 h, before the medium was changed, and photoreceptor cells were added on top of them at a trans‐well insert (0.4 μm pore diameter, Millipore) at a density of 1 × 10^6^ cells/insert. For these experiments, CD medium was supplemented with 5% BSA (Sigma) instead of 3% and medium was not changed but topped up to adequate volumes every 5 days for 21 days.

#### DiI/DiO co‐cultures and pharmacological interventions

After retinal cells were dissociated, cells were labelled with DiI or DiO lipophilic tracers (Thermofisher, 1 μl per 10^6^ cells) in a suspension at 37°C for 15 min in CD medium and then simultaneously washed by centrifugation (300 × *g*, 5 min, RT) for four times with PBS every time in new clean tubes. Supernatant of the final wash was kept and used as control for dye carry over in *wt* retinal cultures. DiI^+^ and DiO^+^ labelled populations were mixed in a ratio 1:1 and plated as stated above for 5 days and on day 5 in culture pharmacological inhibitors were added in appropriate concentrations of Cytochalasin D (Hanna *et al*, 2017) or Latrunculin B (Osteikoetxea‐Molnar *et al*, 2016) for 48 h (Fig EV3). Dose range was based on the published literature and confirmed by a dose–response assessment and determined as the concentration required to significantly decrease the proportion of photoreceptors extending actin^+^ processes but without causing cell detachment and loss of viability. As the inhibitors were dissolved in DMSO, controls were supplemented with the equivalent dose of DMSO (vehicle control).

#### Flow cytometry analysis

After the incubation of co‐cultures with the appropriate drugs/conditions, the cells were detached with pre‐warmed papain/DNAase solution and layered over 1 ml of albumin‐inhibitor solution, centrifuged as above and resuspended in FACS buffer (PBS, 0.05% BSA, 50 mM HEPES and DNAse) containing CD73PE‐Cy7 BioLegend antibody in a dilution 1/300 μl (1,000,000 cells) on ice. Single stain controls and compensation controls were also used to set up the lasers of the cytometer (Fortessa BD). Gating strategy is provided in Fig EV2. The samples acquired with FACS Diva software and further analysed with FlowJo (LLC) program.

#### Live imaging of organelles and cytoskeleton

Cells were grown in 12 mm glass bottom dishes, and after 2 days in culture, the culture medium was replaced with staining solution, which comprised the culture medium and the diluted fluorescent probe, supplemented with the verapamil inhibitor. Live imaging of the dynamics of endosomes/lysosomes and the cytoskeleton of primary photoreceptor cultures performed with the use of SiR‐actin, SiR‐tubulin, SiR700‐lysosome, described in (Lukinavicius *et al*, 2013, 2014), and purchased from Cytoskeleton, Inc. SiR‐actin & SiR‐tubulin and SiR700‐lysosome probes were dissolved in anhydrous DMSO to make a 1 mM stock solution. This was further diluted in the cell medium (phenol red free) in the dilution of 1:10,000 (SiR‐actin & SiR‐tubulin) or 1:5,000 (SiR‐lysosome) supplemented with 10 μM verapamil. Serial dilutions were performed ranging from 1:1,000 to 1:10,000 according to manufacturer’s protocol. The selected dilutions of SiR‐probes could stain the cells without affecting their mobility/viability. Similarly, for mitochondria visualization Mitotracker‐Orange (Invitrogen/Molecular Probes, M7512) was diluted in 1 mM stock in DMSO and then further diluted in 0.01 mM working solution in culture medium from which a final dilution of 1:1,000 was used to stain the primary cells. Post‐labelling the cells were incubated for 3 h of incubation at 37°C at 5%CO_2_ prior live video recording with Leica TCS SP8 confocal microscope equipped with a temperature‐controlled chamber and CO_2_ supply. When acquiring live tissue, xyzt confocal stacks of representative fields of view were captured at resolution ranging from 1,024 × 1,024 to 2,046 × 2,046, step size 0.2–0.5 μm, bidirectional scanning, time‐lapse duration 60 min with 3 min interval using Leica SP8 inverted confocal laser scanning microscope, 63× (oil immersion, NA = 1.3) objectives and photomultiplier tubes to detect fluorescence emission. For SiR‐actin and SiR‐tubulin, the cells were imaged with standard Cy5 filter sets with optimal excitation 650 nm and emission 670 nm, but for SiR700‐lysosome cells were imaged with standard Cy7 filter sets. Excitation was 630–640 nm for SiR700 and SiR emission 650–700 nm (SiR channel) and 700–770 nm (SiR700 channel). For Mitotracker Orange, the 543‐nm line of a green He:Ne laser was used. Images were acquired with the Leica LASX software followed by post hoc analysis using ImageJ.

#### Post‐acquisition confocal image analysis

3D reconstruction was demonstrated as surface (organelles/cytoskeleton) per volume (cGFP) for culture live video recordings. For the visualization of NT‐like structures in culture and *in vivo*, Otsu watershed segmentation of appropriately thresholded cGFP surface was performed. 3D reconstructions and applied deconvolution post‐acquisition was performed with HyVolution software (Scientific Volume Imaging Huygens). xyz drift correction post‐acquisition from live video recordings was performed with object stabilizer with HyVolution software. Movies or live video recordings or 3D reconstruction rotations were generated as mp4 files via Movie maker plugin of HyVolution software and were further edited with Photoshop 2020 (version 21.2).

#### Object tracking analysis

Lysosomes and mitochondria movement was imaged as described above and was analysed with TrackMate plugin of ImageJ Software (Tinevez *et al*, 2017). Resolution 1,024 × 1,024, z step size 0.5 μm, bidirectional scanning, time‐lapse duration 60 min with 3‐min interval using Leica SP8 inverted confocal laser scanning microscope, 63× (oil immersion, NA = 1.3) objectives and photomultiplier tubes to detect fluorescence emission. Briefly, lif files of single cells or connected cell pairs were loaded as maximum z projections of 8 bit colour xyt images. Tracks analysed with DoG detector segmentation in organelle channel; for lysosomes blob diameter = 0.6 microns/threshold = 2.5, for mitochondria blob diameter=1 microns/threshold = 2.5, with enabled median filter and sub‐pixel localization. Initial threshold was further manually adjusted to eliminate artificial generated tracks. Hyperstack displayer view was selected, and appropriate filters on spots were adjusted appropriately where needed. Simple LAP tracker algorithm was selected, and linking max distance/ gap closing max distance was the length between the outer edge of the cell cytoplasm of both interconnected cells or the single cell length composed from the cell body and the processes (ex 15 microns) gap closing max frame=2. Track feature analyses: Branching: N spots, Gaps, Longest gap, Splits, Merges, Complex.; Track duration provides: Duration, T start, T stop, Displacement.; Track index provides: Index, ID.; Track location provides: X, Y, Z.; Velocity provides: Mean V, Max V, Min V, Median V, V std.; TRACK_SPOT_QUALITY provides: Mean Q, Max Q, Min Q, Median Q, Q std. Analysis was further assessed by the Track‐Scheme analyser and methodology/data/images of tracks exported as doc, xls and tiff files, respectively.

#### NT Depth localization analysis

The cells were imaged as above after 2–3 days in culture and temporal colour‐coding hyper‐stacks were generated with ImageJ to analyse their localization within the z dimension (Fig 3). 16 colour grade scale was selected and according to this scale, blue shades (7.7–10 μm) indicate free floating processes/cells, yellow to orange shades (2.6–7.7 μm) indicate depths in the middle of the dish, while purple to white colours (0–2.6 μm) indicate proximity to the dish surface. Neurites versus nanotube‐like processes were assessed according to the criteria laid out in Appendix Table S1, manually quantified and plotted as percentages normalized to cell numbers.

#### cGFP Fluorescent recovery after photobleaching (FRAP) of *Nrl.Gfp* Photoreceptor primary cultures

FRAP is the most commonly used bulk kinetics approach to study the dynamics of protein mobility. The assay was performed at 37°C and with 5% CO_2_ in PR culture medium and followed the general guidelines of FRAP experiment described by Koskinen and Hotulainen (Koskinen & Hotulainen, 2014). For the purposes of revealing the mobility of cGFP between two interconnecting cells, cell clusters or single cells, the cGFP was photobleached within the round area that surrounds the whole cell body cytoplasm (ROI). For the confirmation of nanotube‐connected cells, xyz imaging in 1,024 × 1,024 resolution as described above occurred prior photobleaching and nanotube processes vs neurites were classified according to the criteria of Appendix Table S1. The frame including the ROI (diameter ˜ 8 μm) was imaged five times before bleaching with 512 × 512, 2× digital zoom, 63× oil immersion inverted lens, resolution settings and frame average = 3 with bidirectional on, power of 700 Hz and pinhole = 2 Airy Units. Photobleaching was achieved with three scans (total bleach time 2.589 s) of the region of interest with 100% laser power at the sample (442/405 diode). Imaging of the area was resumed immediately after photo‐bleaching and continued every ti = 0.863 ms for a duration *t* = 215 s with normal scanning with approximately 10% power from image acquisition settings. The recording of the last frames was used based on FRAP in single cells to ensure a plateau is reached. All the post‐bleach values were double normalized hence, divided by the values from the non‐bleached area of the cells and normalized to the first pre‐bleach value. The first post‐bleach measurement was set to 0 s. The analysis of the FRAP recovery data was performed as described in the text using LAS AF (Leica Microsystems, Germany), ImageJ, Excel (Microsoft) software. Double normalized values were then fitted with double association exponential over a single exponential equation, as required, providing a set of two *t*_1/2_ values (fast and slow) as shown below in GraphPad prism 8 software, and fitted curves are represented of an average of *n* ˜ 40 cells (Fig 4) per condition.

#### Primary photoreceptor culture SEM

Sample preparation was performed by Dr Matt Hayes, UCL Institute of Ophthalmology Imaging Facility. For this purpose, photoreceptors were cultured in glass coverslips or cultured in 12 mm, hydrophilic PTFE, 0.4 µm pore culture inserts for 3 days in culture, washed with HBSS ^wCa/Mg^ (Invitrogen) and then fixed overnight with in Karnovsky’s Glutaraldehyde solution (3% glutaraldehyde / 1% PFA (sigma, UK) buffered to pH 7.4 with 0.08 sodium cacodylate‐HCl). The samples then osmicated and dehydrated as described above and further dehydrated by rapid immersion in hexamethyldisilazane (Sigma‐Aldrich, USA) for 2 min and then were allowed to dry on a conductive carbon‐tab on an aluminium SEM stub. The sample was ‘back‐filled’ with silver paint to promote conductivity, allowed to dry for 2 h in a desiccator and platinum‐coated (1.5 nm) in a sputter‐coater (Cressington 108 auto, Cressington, UK). Samples were examined using a Zeiss Sigma SEM using both in‐lens and VP‐back scatter detectors (Zeiss, Germany). Imaging settings; InLens, aperture size 30 mm, WW 3.3 mm, 14.24 K X EHT = 3 kV.

#### EVs enrichment from primary cultures

EV enrichment from primary culture supernatants was based on the protocol described by Thery and colleagues (Thery *et al*, 2006), with the modifications proposed by Kowal and colleagues (Kowal *et al*, 2016) (see also (Thery *et al*, 2018)). To generate adequate numbers of 100 K small EVs for characterization and experimentation, a pool of three independent cultures was required >90*10^6^ photoreceptor cells. Cells were cultured in absence of serum in a chemically defined medium (CM) that was collected every 48 h for 7 days from primary photoreceptor cultures. Briefly, the cell supernatant was centrifuged at 350 × *g* for 10 min at 4°C pellet was discarded, then at 2,000 × *g* for 20 min at 4°C (2 K pellet was retrieved from this step), and then 10,000 rpm for 60 min at 4°C (10 K pellet was retrieved from this step). Remaining supernatant was filtered with 0.22 µm PVDF filter (Millipore) and transfer into ultra‐centrifuge tubes (30 ml, Beckman), and spun at 4°C, for ˜ 3 h at 25,000 rpm on a SW 32Ti rotor 38 ml tubes. The supernatant was then discarded, and the tubes drained in tissue paper, the 100 K pellets retrieved from this step were then reconstituted in 100 μl PBS, allowed on ice for 1 h and vortexed thoroughly before aliquoted and stored at −80°C.

#### DLS Analysis of 100 K small EVs

Dynamic light scattering (DLS) was used to measure particle biophysical properties (size) as describe by Van Der Pol and colleagues (van der Pol *et al*, 2010). All measurements were taken with Zetasizer Nano ZS (Malvern, UK). Briefly, samples allowed to equilibrate from 4°C to 25°C. Particles dispersed in sterile PBS EM grade in dilutions varying from 1:2 to 1:100. Liquid refractive index and viscosity values for 25°C are: viscosity of 0.8882 cPoise, refractive index of 1.33 and dielectric constant of 79.0. The intensity changes are analysed with a digital autocorrelator, which generates a correlation function. This curve was analysed to give the size and the size distribution. Due to the polydispersity of EV samples, results are presented in intensity and volume per size plots for further clarity. This is because in some cases small intensity peaks may disappear in the transformation from intensity to volume or number of particles (according to manufacturer Malvern Panalytical). All data generated (PDI, size intensity, volume with Mie Theory) were saved in excel files and plots were generated with GraphPad Prism 8. If PDI was high, (example > 0.7 measurement was omitted).

#### TEM analysis of photoreceptor derived EVs

Transmission Electron Microscopy (TEM) was used to visualize the various EV pellets and to confirm their identity. Briefly, fresh samples of 2 K, 10 K and 100 K pellets were resuspended in fixation buffer containing 2% PFA, 5% Sucrose. EV samples were allowed to be absorbed by Formvar‐carbon coated EM grids 20 min RT (Agar scientific), washed with PBS and then fixed with 1% Glutaraldehyde 5 min RT and washed with ddH_2_O. Negative staining performed with 1% uranyl‐acetate solution (UA) (pH = 7) for 5 min, followed by methyl‐cellulose UA incubation for 10 min on ice and the excess fluid was gently absorbed with a Whatman filter paper. Grids allowed to air dry for 5 min and stored in appropriate grid storage boxes. Grids were analysed using a JEOL 1010 Transmission Electron Microscope (80 kV), fitted with a digital camera for image capture.

#### Protein quantification of EV pellets or cells

EV pellets or cell pellets were resuspended in RIPA buffer (10 mM Tris‐Cl (pH 8.0), 1 mM EDTA, 1% Triton X‐100, 0.1% sodium deoxycholate, 0.1% SDS, 140 mM NaCl, 1 mM PMSF, Invitrogen, USA) supplemented with 1% v/v protease inhibitor cocktail. Further disruption occurred with sonication 4 × 10 s on ice. Lysates were allowed to recover on a rocking plate for 30 min at 4°C. 5 µl of supernatant was used for protein estimation with a DC Assay (Bio‐Rad, USA), according to manufacturer’s instructions. Samples that were used for dot blot analysis were further diluted in SDS lysis buffer (see below) to achieve a concentration of 2.5 μg/dot, whereas samples that used for Western blot analysis were diluted to a final concentration of 30 μg/lane.

#### Western blotting analysis

For Western blotting, further denaturation of cell lysates occurred with the addition of 5% beta‐mercaptoethanol in in 1× Laemmli sample buffer (40% Glycerol, 240 mM Tris/HCl pH 6.8, 8% SDS, 0.04% bromophenol blue, Bio‐Rad) and incubation at 95°C for 5 min. Samples were loaded into an acrylamide gel gradient of 4–15% for SDS–PAGE analysis. Protein separation took place in a voltage of 180–220 V for 40 min – 1 h, followed by wet‐transfer in pre‐activated with methanol PVDF membrane (Millipore) at 100 mA for 3 h at 4°C. Membranes were subsequently blocked in blocking buffer (5% milk in Tris‐buffered saline (TBS) with 0.05% Tween 20) for 1 h at RT with agitation. Primary antibodies (RhoA, (67B9) Rabbit mAb #2117 Cell Signaling; Rac, ARC03‐S, Cytoskeleton Inc; beta actin; A2228, Sigma) were diluted in the blocking solution, supplemented with 0.01% NaN3 and incubated overnight at 4°C with agitation. Blots were washed with TBS with Tween‐20 (TBS‐T). Horseradish peroxidase (HRP)‐conjugated secondary antibodies (DAKO) were diluted in TBS‐T (1:5000). Blots were then incubated in the secondary solution at RT with agitation for 1 h. Protein bands were visualized using Immobilon™ Western Chemiluminescent HRP substrate kit (Millipore, USA). The resulting blot was imaged with a ChemiDoc TM MP Imaging System (Bio‐Rad, USA).

#### Filter trap assay (Dot Blot)

EV pellets and cell pellets were resuspended in SDS lysis buffer [10 mM Tris–HCl buffer, pH 8.0, containing 150 mM NaCl and 2% (w/v) SDS] supplemented with 1% (v/v) Protease inhibitor cocktail (Sigma). Cell disruption was completed by sonication. Undiluted samples were loaded onto a 0.45 µm PVDF membrane activated with 100% Met‐OH and pre‐equilibrated with SDS wash buffer [10 mM Tris–HCl buffer, pH 8.0, containing 150 mM NaCl and 0.1% (w/v) SDS]. After loading, 0.1% (w/v) SDS wash buffer was added to each well. Samples allowed to flow through the membrane by gravity for 20 min at 22°C. Thereafter, membranes were washed three times in SDS wash buffer and subjected to a vacuum (Lai *et al*, 2015). Membranes were blocked with 5% milk in TBS‐T‐0.05% or 5% BSA in TBS‐T‐0.05% for 1 h RT and then immunoblotted with primary antibodies listed below. Primary antibodies were diluted in the blocking solution and supplemented with 0.01% NaN3. Primary antibodies for the following epitopes were used; Beta actin (MAB8929, R&D Systems,1:5,000), CD63 (MAB5417, R&D Systems, 1:1,000), CD81 (10037, Cell Signalling, 1:1,000), TSG101 (612696, BD Transd, 1:1,000), CD9 (CBL162, EDM Millipore, 1:1,000), Alix (2171, Cell Signalling, 1:1,000), Cre recombinase (MAB3120, EDM Millipore, 1:1,000), GM130 (12480, Cell Signalling, 1:1,000), Rhodopsin (O4886, Sigma, 1:5,000), G‐alpha‐t1 (Sc389, Santa Cruz INC, 1:500), Recoverin (AB5585, MERCK, 1:5,000) and GFP/CFP/RFP (632380, Clontech, 1:2,000). Blots were washed with TBS with Tween‐20 (TBS‐T) × 3, 5 min each, at RT with agitation. Horseradish peroxidase (HRP)‐conjugated secondary antibodies (Sigma) were diluted in TBS‐T (1:5,000). Results visualized using Immobilon™ Western Chemiluminescent HRP substrate kit (Millipore, USA) following the manufacturer’s instructions. Blots were imaged with a ChemiDoc TM MP Imaging System (Bio‐Rad, USA).

#### Real‐time quantitative PCR in small EVs

To assess the potential presence of RNA within EVs, 100 K EV pellets were treated as described by Valadi and colleagues (Valadi *et al*, 2007) with 0.4 μg/μl RNase (Sigma) for 10 min at 37°C in reactions of 50 µl in PBS. After incubation, 1 U/µl RNase Inhibitor (Super RNAse Out 20 U/µl, Thermo Fisher) was added to the mixture and incubated at RT for 2 min. Mixtures were additionally treated with 0.25% trypsin for 10 min at 37°C. After incubation, samples were diluted in 3 ml sterile EM grade PBS and ultra‐centrifuged for 2 h at 100 K (55 K A100, Beckman) at 10°C. Supernatant was discarded, and samples resuspended in 200 µl PBS to further purify RNA and left for 30 min at RT with discontinuous vortexing of 10 s. As a control, 5 μg cellular RNA was added to the control EVs before the RNase treatment. RNA extraction was performed with Qiagen RNeasy^®^ Mini kit according to manufacturer’s instructions. Whole transcriptome Reverse transcription PCR performed with QuantiTect^®^ Whole Transcriptome (Qiagen) kit according to manufacturer’s instructions. For the RTQ‐PCR analysis, cDNA 50 ng/µl were diluted 1:16 in TaqMan^®^ Universal PCR Master Mix (Roche, UK), 2 µM forward primer, 2 µM reverse primer and 0.2 µM probe (Universal Probe Library; Roche, Germany). Primers for the target markers and endogenous reference control (β‐actin) were designed, and appropriate hydrolysis probes were chosen via the Universal Probe Library Design Centre (Roche, UK). RTQ‐PCR was run on an ABI Prism 7900HT Fast Real‐time Sequence Detection System (Applied Bioscience, USA). All samples were run in triplicates. The general conditions used had an initial denaturation at 94°C of 2 min. Reactions followed by 40 cycles of: Denaturing step at 95°C for 30 s, Annealing step at 42°C for 30 s, Extension step at 72°C for 1 min, and final extension of 5 min at 72°C was performed to finish the reaction. The relative expression between comparable samples in relation to the expression of the genes was normalized using the following methodology: signal thresholds were first manually determined and maintained for each individual experimental run on Sequence Detection Systems software 2.2.2 (Applied Biosystems, USA). To normalize target against β‐actin control expression levels, cycle numbers at threshold (Ct) were used to calculate ΔCt. With the formula; ΔCt = Ct (target) – Ct (β‐actin). To normalize sample (e.g. treated) against control levels of target expression (e.g. nontreated), ΔΔCt was calculated by the formula ΔΔCt = ΔCt (sample) – ΔCt (control). Finally, to obtain normalized relative target expression levels in ‘% of control levels’, the following formula was applied Normalized expression levels (%) = (2‐ΔΔCt) × 100. The following primer/probe combinations were used; Gfp: GAAGCGCGATGACATGGT/CCATGCCGAGAGTGATCC/ probe 67, Cre: ATCTGGCATTTCTGGGGATTG/ GCAACACCATTTTTTCTGACCC/ probe 20, Gnat1: AGAGCTGGAGAAGAAGCTGAAA/ TAGTGCTCTTCCCGGATTCA/ probe 89, Rho: ACCTGGATCATGGCGTT/ TGCCCTCAGGGATGTACC/probe 32, Rcvrn: CAATGGGACCATCAGCAAA/ CCTCAGGCTTGATCATTTTGA/ probe 67, Crx: CCCCAATGTGGACCTGAT/GGCTCCTGGTGAATGTGGT/ probe 64, Nrl; TTCTGGTTCTGACAGTGACTACG/ TGGGACTGAGCAGAGAGAGG/ probe 53, Actb:AAGGCCAACCGTGAAAAGAT/GTGGTACGACCAGAGGCATAC/probe 56.

#### AAV production

##### *Nrl* promoter‐driven expression constructs

*Cre* was subcloned into a pD10 expression and AAV packaging‐compatible construct downstream of either the CMV promoter or the *NRL* promoter region. A 2.5 kb segment upstream of the *Nrl* gene was cloned from mouse genome and used as the *NRL* promoter in this study, as per (Akimoto *et al*, 2006). *Nrl.Cre* and *CMV.Cre* plasmid constructs were used to produce AAVShH10‐Y445F (herein termed *AAV‐Nrl.Cre* and *AAV‐CMV.Cre*). Recombinant AAVShH10‐Y445F vectors were produced following the tripartite transfection method, which required the transfection of three plasmids (pD10 vector containing transgene with packaging signal, AAV capsid plasmid ShH10 and pDHelper) in HEK293T cells as previously described(Gonzalez‐Cordero *et al*, 2018). Briefly, HEK293T cells were grown in D10 cell culture medium (DMEM supplemented with 10% foetal calf serum (Gibco, USA) and antibiotics (Gibco, USA)) at 37°C / 5% CO_2_ until the cells reached 70% confluency. The transfection mix was prepared with the appropriate plasmids and polyethylenimine 2 mg/ml (PEI) transfection reagent. The cell factory was then left to incubate at 37°C / 5% CO_2_ for three days. Three days post‐transfection, the cells were harvested and centrifuged for 20 min at 3,500 × *g*. Cell pellets were then resuspended in TD buffer (140 mM NaCl / 5 mM KCl / 0.7 mM K_2_HPO_4_ / 3.5 mM MgCl_2_ / 25 mM Tris base in ddH2O, pH 7.5) and lysed by 4 freeze/thaw/vortex cycles to release the viral particles, followed by enzymatic lysis using 50 U/ml benzonase (Sigma, USA) for 30 min at 37°C. Subsequently, the solution was then centrifuged at 18,000 × *g* for 30 min. The supernatant was then filtered through 0.45‐μm syringe filters to remove cell debris and purified using affinity chromatography (AVB Sepharose column, GE Healthcare, USA). The final virus preparation was resuspended in PBS‐MK buffer (0.1 M phosphate‐buffered saline (PBS) / 2.5 mM KCl / 1 mM MgCl2) and concentrated with Vivaspin 6 columns to a final volume of 200–250 μl. The virus was then aliquoted and stored at −80°C. qPCR analysis indicated that the virus titre as viral particles/ml.

#### Lentivirus production

pLenti plasmids; pLenti‐CAG‐GFP‐P2A, pLenti‐CAG‐RhoA‐GFP‐P2A and pLenti‐CAG‐ΔNRac‐GFP‐P2A were a kind gift generated by Dr Ortín‐Martínez Arturo and Professor Valerie Wallace, University of Toronto. Lentivirus production performed by Dr Mark Basche and followed the tripartite transfection method in HEK293T cells similar to AAV production, which required the transfection of three plasmids (pLenti‐containing transgene with packaging signal, envelope plasmid pMD2.G, helper plasmid pCMVΔR8.74) diluted in OptiMEM. In this case, 80 µg DNA per 15 cm dish in DMEM^+^PEI were used for transfection in a total volume of ˜ 2.7 ml for 5 h. Cell medium was then replaced with D10, and the cells were incubated at 37°C, 5% CO_2_ for 48 h. Harvest of the cell supernatant containing the lentiviral particles took place 48 and 72 h post‐transfection. Simply the cell supernatant was then sequentially centrifuged at 350 × *g* for 10 min at 4°C, then at 2,000 × g for 20 min at 4°C and then 10,000 rpm for 60 min at 4°C to remove course cell debris, then filtered with 0.22 µm PVDF filter (Millipore) and transfer into ultra‐centrifuge tubes (30 ml, Beckman), and spun at 4°C, for ˜ 3 h at 23,000 rpm on a SW 32Ti rotor 38 ml tubes. The supernatant was then discarded, and the tubes drained in tissue paper, the pellets were then reconstituted in 100 μl OptiMEM, allowed on ice for 1 h and vortexed thoroughly before aliquoted and stored at −80°C. Quantification performed with infecting HEK293T in serial dilutions of virus and assessed via cell counting based on GFP expression levels.

#### Statistical analysis

All the data analysis is shown as means ± standard deviation (SD) of the mean, unless otherwise indicated. Generation of plots, curve fitting and statistical analyses were performed using GraphPad Prism 8 for Windows Version 10 (GraphPad Software Inc). Statistical significance was assessed using non‐parametric one‐way analysis of variance (ANOVA) with Dunnett's multiple comparison *post hoc* test (compared all groups against control group) or Bonferroni multiple comparisons *post hoc* test (compared all groups or selected groups) or as stated in figure legends. *N* and *n* numbers as stated below, unless indicated; cell cultures; *N* = number of experiments derived from an independent litter dissociation and/or isolation and consecutive cell culture, *n* = number of wells, chambers, coverslips used. Processes counts; *n* = number of processes counted from a pool of individual isolations/cultures (3 individual experiments or more, unless stated). Cell counts; *n* = number of cell’s somata/nuclei counted from a pool of individual isolations/cultures (three individual experiments or more, unless stated). Animal work: *N* = number of cohorts of independent injections/treatments, *n* = number of eyes of all cohorts pooled. Flow cytometry: *N* = number of experiments derived from an independent litter dissociation/ isolation and consecutive cell culture, *n* = number of wells used per condition. *P*‐values; *P* < 0.05 = statistically significant*, *P* < 0.01 = very significant**, *P* < 0.001 = highly significant***

#### *Post hoc* image processing

Figure panels were generated with Photoshop software (version 22.3.1). For better visualization, some automatically generated scale bars were covered and redrawn. Image stitching after tile scans acquired using 40× magnification were performed automatically in LasX software and ImageJ.

## Author contributions

Conceptualization: AAK, RRA, RAP; Methodology: AAK, AJS, RRA, RAP; Formal analysis: AKK, RAP; Investigation: AAK, MB, AH, ELW; Resources: RRA, RAP; Writing—original draft: AAK, RAP; Writing—review & editing: AAK, AJS, RRA, RAP; Funding acquisition: RRA, RAP; Supervision: RAP.

## Conflict of interest

The authors declare that they have no conflict of interest.

## Supporting information



Appendix

Expanded View Figures PDF

Movie EV1

Movie EV2

Movie EV3

Movie EV4

Movie EV5

Movie EV6

Movie EV7

## Data Availability

This study includes no data deposited in external repositories.
