# Supplementary material for: Nanotube‐like processes facilitate material transfer between photoreceptors
Source: EMBO Rep. 2021 Sep 8;22(11):e53732. doi: 10.15252/embr.202153732 (PMC8567251; doi:10.15252/embr.202153732)
Supplement: Supplementary file 1 — Appendix [file EMBR-22-e53732-s003.pdf]

Appendix

Nanotube-like processes facilitate material transfer between photoreceptors

Aikaterini A. Kalargyrou[1,2,#], Mark Basche[1,2], Aura Hare[1,2], Emma L. West[1,2], Alexander J. Smith[1,2], Robin R. Ali[1-3], Rachael A. Pearson[1,2,#]

[1] University College London Institute of Ophthalmology, 11-43 Bath Street, London, EC1V 9EL; [2] Centre for Cell and Gene Therapy, King's College London, 8th Floor Tower Wing, Guy's Hospital, London SE1 9RT; [3] Kellogg Eye Center, University of Michigan, 1000 Wall St., Ann Arbor, Michigan 48105, USA.

Appendix Table of Contents

**Appendix Table S1.** Morphological characteristics of the different types of processes exhibited by purified photoreceptor cells in culture.
**Appendix Figure S1:** Crossing of *Nrl.Cre*$^{+/-}$ *and mTmG*^{floxed} reporter transgenic animals yields photoreceptor-specific Cre-mediated recombination.
**Appendix Figure S2:** Transplantation of UV-dead P8 *Nrl*.Gfp$^{+/+}$ photoreceptors does not result in cGFP transfer.

Appendix table S1

**Nanotube (NT)-like**	**Neurites**	**Segment-like**
A process without defined limits that directly connects two cells.<br><br>Extends directly to connected cell<br><br>No visible terminus	A process with discrete limits that is extended by a neuronal cell.<br><br>May protrude in any direction in culture.<br><br>May show filopodia-like extensions and/or brush border<br><br>N.B. Synaptic terminals are specialised connections between neurites requiring pre- and post-synaptic cells. Not formed between neighbouring rods.	A specialized cilium with a bulbous, membranous end. May express photopigments such as rhodopsin.<br><br>Note that, *in vivo*, the segment comprises of inner and outer domains, but these distinctions are rarely established in dissociated cultures.
Not attached to the substratum (in culture)	Typically attached to the substratum (in culture)	Typically attached to the substratum (in culture)
Vulnerable to fixation	Not vulnerable to usual fixation methods	Not vulnerable to usual fixation methods
Variable length, but typically short (< 10 μm)	Variable length	
Straight, but longer length connections may exhibit some curvature	May exhibit curvature	May exhibit curvature
No secondary branching	May exhibit secondary branching	No secondary branching
Either: Actin-rich (thin < 0.7 μm, Type I)<br>or<br>Actin/Tubulin-rich (thick > 0.7 μm, Type II)	Contain both actin and microtubules.<br><br>Actin-rich forms are associated with brush-border growth-cone terminals.	Tubulin and actin-rich inner segments.<br><br>Usually thick structures.

Appendix Table S1. Morphological characteristics of the different types of processes exhibited by purified photoreceptor cells in culture.

Photoreceptor processes assessed using fluorescent live imaging of *Nrl.Gfp*$^{+/+}$ photoreceptors, were used to classify them as: NT-like, neurites and nascent inner segment-like protrusions.

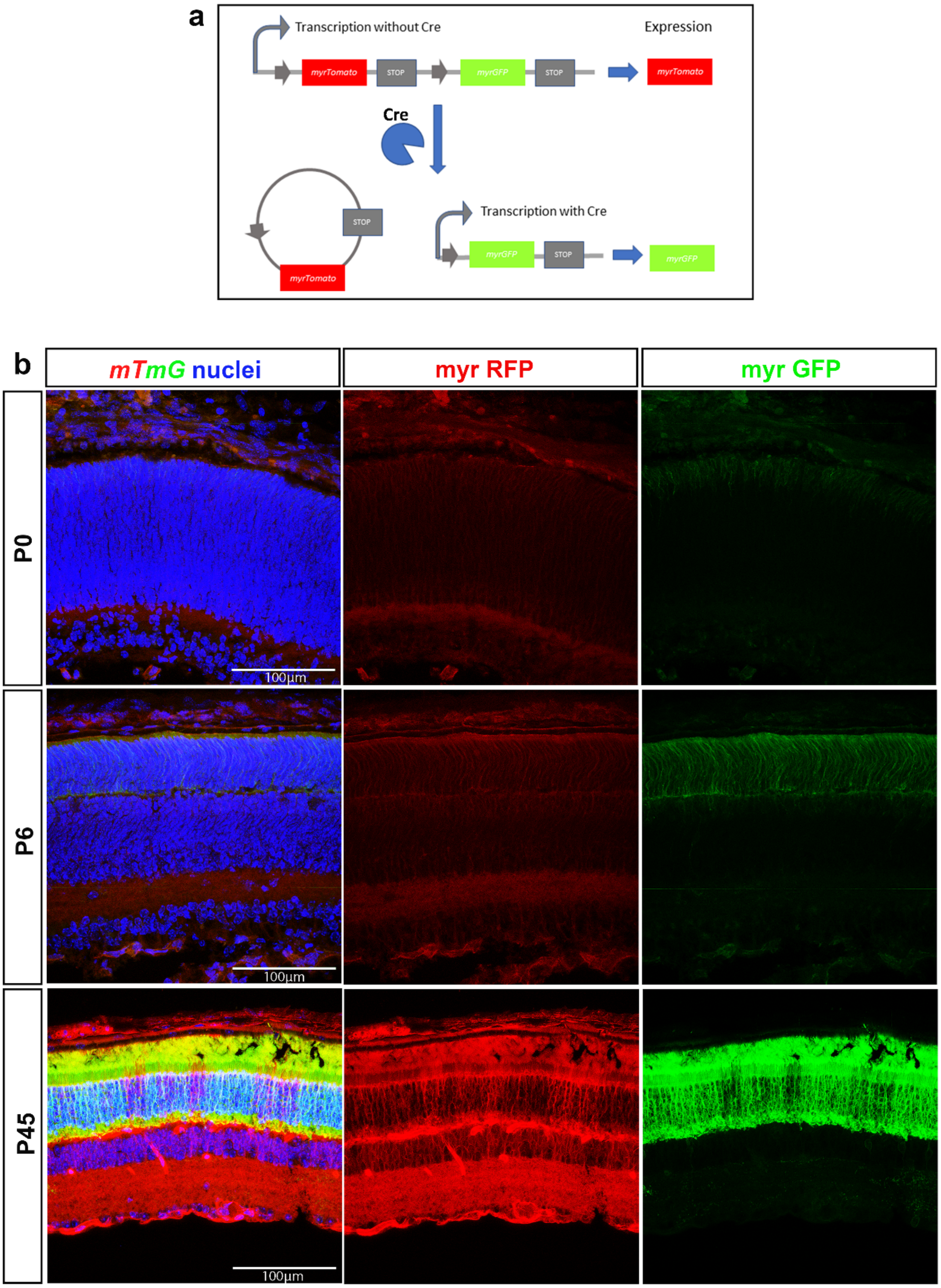

Appendix Figure S1: Crossing of *Nrl.Cre*$^{+/-}$ *and mTmG*^{floxed} reporter transgenic animals yields photoreceptor-specific Cre-mediated recombination.

a, Schematic representation of *mTmG* reporter transgenic line fluorescent reporter expression in absence or presence of Cre recombinase;

b, Representative confocal MIP images of *Nrl.Cre*$^{+/-}$ *x mTmG*^{floxed} eye cups at postnatal day (P)0, 6 and 45. All cells express myrRFP (*red*) unless they undergo Cre-mediated recombination, which reduces expression of myrRFP and switches on expression of myrGFP (*green*). Visible rod-specific recombination begins at P0, increasing by P6 and is widespread throughout the photoreceptor layer by P45. MyrGFP was not seen in any other layers of the retina, confirming the specificity of the *Nrl* promoter. *Blue* = Dapi (nuclei), *red* = myrRFP, *green* – myrGFP. Images are representative of N = 5 animals / timepoint. Scale bar = 100µm.

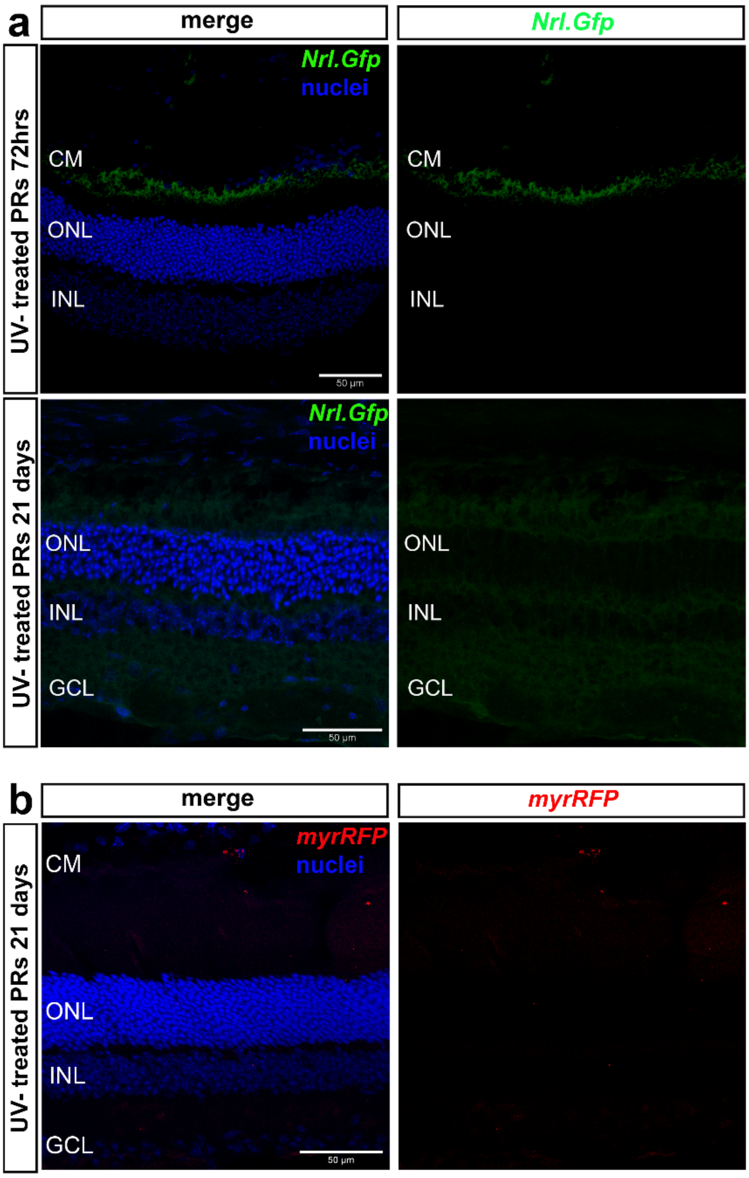

Appendix Figure S2: Transplantation of UV-dead P8 *Nrl*.Gfp$^{+/+}$ photoreceptors does not result in cGFP transfer.

a & b, Representative MIP images of wildtype hosts receiving subretinal transplants of **a**, UV-treated (dead) P8 *Nrl*.Gfp$^{+/+}$ photoreceptors and **b,** UV-treated (dead) P8 *myrRFP*$^{+/+}$

photoreceptors. N=3 Eyes were fixed and examined at 96h post transplantation or 21 days post-transplantation.

Data information CM = cell mass, ONL = outer nuclear layer, INL = inner nuclear layer, GCL = ganglion cell layer. *green* = GFP; *blue* = nuclei.
