# Supplementary material for: Nanotube‐like processes facilitate material transfer between photoreceptors
Source: EMBO Rep. 2021 Sep 8;22(11):e53732. doi: 10.15252/embr.202153732 (PMC8567251; doi:10.15252/embr.202153732)
Supplement: Supplementary file 2 — Expanded View Figures PDF [file EMBR-22-e53732-s009.pdf]

Expanded View Figures

Figure EV1. Photoreceptor-derived EV subpopulations contain Cre and GFP protein.

A–C Representative dot blots of GFP (A), Cre (B) and GM130 (C) expression in P8 *Nrl.Cre*$^{+/-}$, *Nrl.Gfp*$^{+/+}$, *Nrl.Cre*$^{+/-}$ × *mTmG*$^{+/+}$ (*myrGFP*) photoreceptor-derived 100 K EV pellets, as appropriate ($N = 8$ experiments, each dot represents a pool from three independent EV isolations, derived from $60*10^6$ cells from two samples). The lack of GM130 staining confirms the absence of contamination from Golgi within the EV preparations. Positive GM130 staining from whole cell lysate is shown in Fig 1.

D Representative tile-scan images following (*left*) subretinal and (*middle*) intravitreal injection of EVs (100 K fraction) derived from P8 *Nrl.Cre*$^{+/-}$ photoreceptors, compared with (*right*) subretinal injection of EVs (100 K fraction) derived from non-photoreceptors (*Nrl.Cre*$^{+/-}$); *red* = *TdTomato* recombined cells; *blue* = nuclei; Scale bar = 100 μm (*left* & middle) and 50 μm (right).

© 2021 The Authors

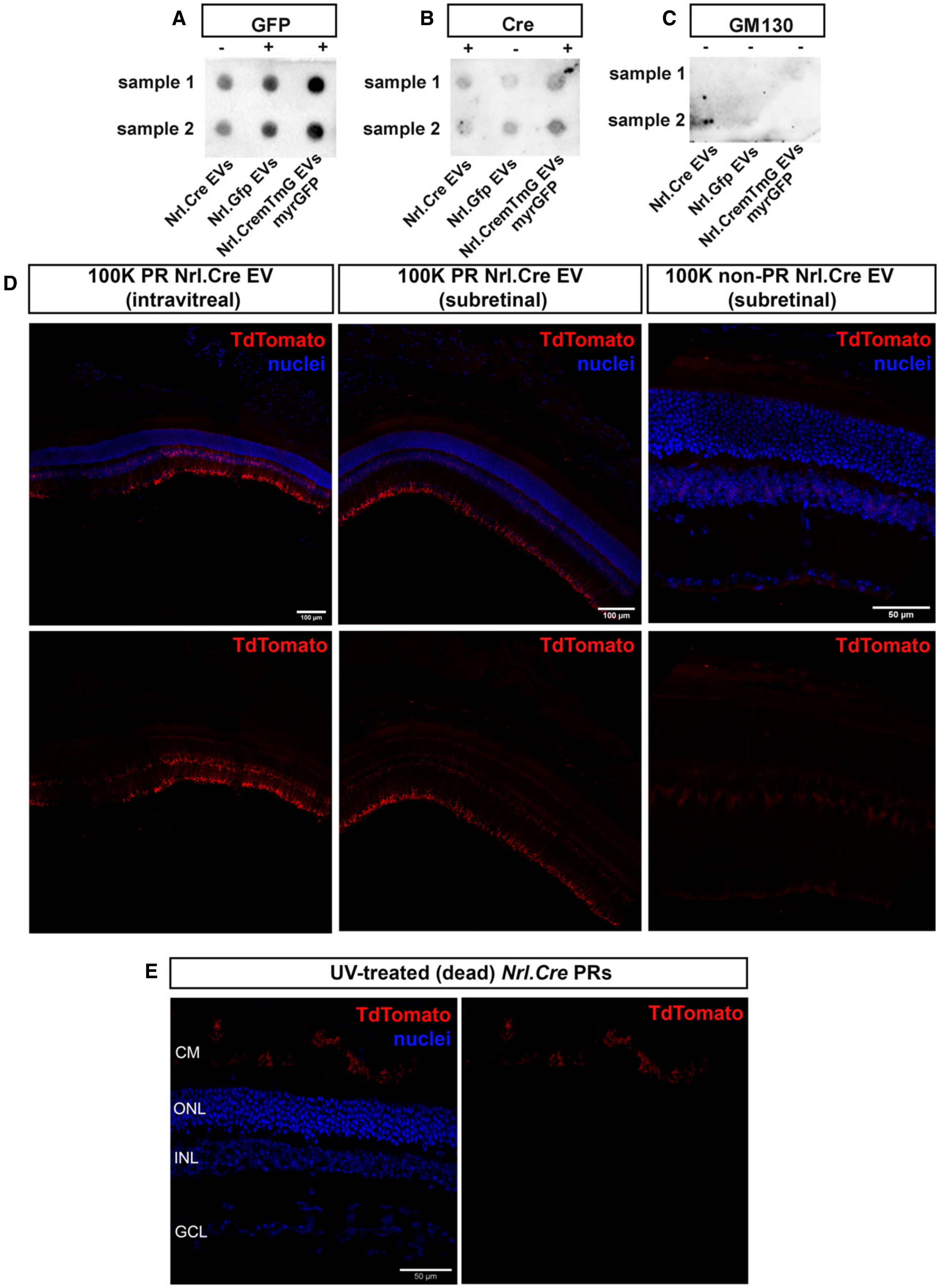
A
GFP
- + +
sample 1
sample 2
Nrl.Cre EVs
Nrl.Gfp EVs
Nrl.CremTmG EVs myrGFP
B
Cre
+ - +
sample 1
sample 2
Nrl.Cre EVs
Nrl.Gfp EVs
Nrl.CremTmG EVs myrGFP
C
GM130
- - -
sample 1
sample 2
Nrl.Cre EVs
Nrl.Gfp EVs
Nrl.CremTmG EVs myrGFP
D
100K PR Nrl.Cre EV (intravitreal)
100K PR Nrl.Cre EV (subretinal)
100K non-PR Nrl.Cre EV (subretinal)
TdTomato
nuclei
100 µm
50 µm
E
UV-treated (dead) Nrl.Cre PRs
CM
ONL
INL
GCL
50 µm

Figure EV1.

Figure EV2. Photoreceptors form nanotube-like processes in culture.

A Representative MIP example of rhodopsin immunostaining of fixed *Nrl.Gfp*$^{+/+}$ photoreceptors at 6 DIC. Digitally enhanced microphotographs of a neurite (*solid line rectangle*) and an inner segment-like process (*dotted rectangle*), showing strong rhodopsin staining in the bulbous inner segment-like structure (*yellow arrow*), compared to more dispersed staining in a neurite (*blue arrows*). Green = *myrGFP*, Magenta = Rhodopsin; Scale bar = 5 μm.

B Representative MIP example of live imaging of P8 *Nrl.Cre*$^{+/-}$ × *mTmG*$^{+/+}$ (*myrGFP*) photoreceptor processes. SiR-actin (*magenta*) reveals the presence of actin puncta (*white asterisk*) at the point of contact between a nanotube-like process (herein termed ^{Ph}NTs) and an adjacent photoreceptor. *Green* = *myrGFP*, *Magenta* = SiR-actin; Scale bar = 10 μm.

C–E Representative 3D deconvolved images of ^{Ph}NTs from *Nrl.Gfp*$^{+/+}$ (*green*) P8 photoreceptor live-imaged at DIV1–3 and labelled with SiR-actin (*red*). (C) (*left*) Simulated Fluorescence Process (SFP) image of an example of a short ^{Ph}NT and (*middle, right*) digitally enhanced 3D rendered surface per volume images of the same ^{Ph}NT in rotation; Scale bar = 1 μm; (D) (*left*) SFP image of an example of a long ^{Ph}NT and (*middle, right*) digitally enhanced 3D rendered surface per volume images of the same ^{Ph}NT in rotation. Representative images from *N* = 7 independent cultures. Scale bar = 4 μm; (E) (*left*) SFP and (*middle, right*) digitally enhanced 3D rendered surface per volume images of the ^{Ph}NT shown in Fig 3J. Scale bar = 4 μm; 3 μm; 2 μm; 3 μm, respectively, from left to right.

 © 2021 The Authors

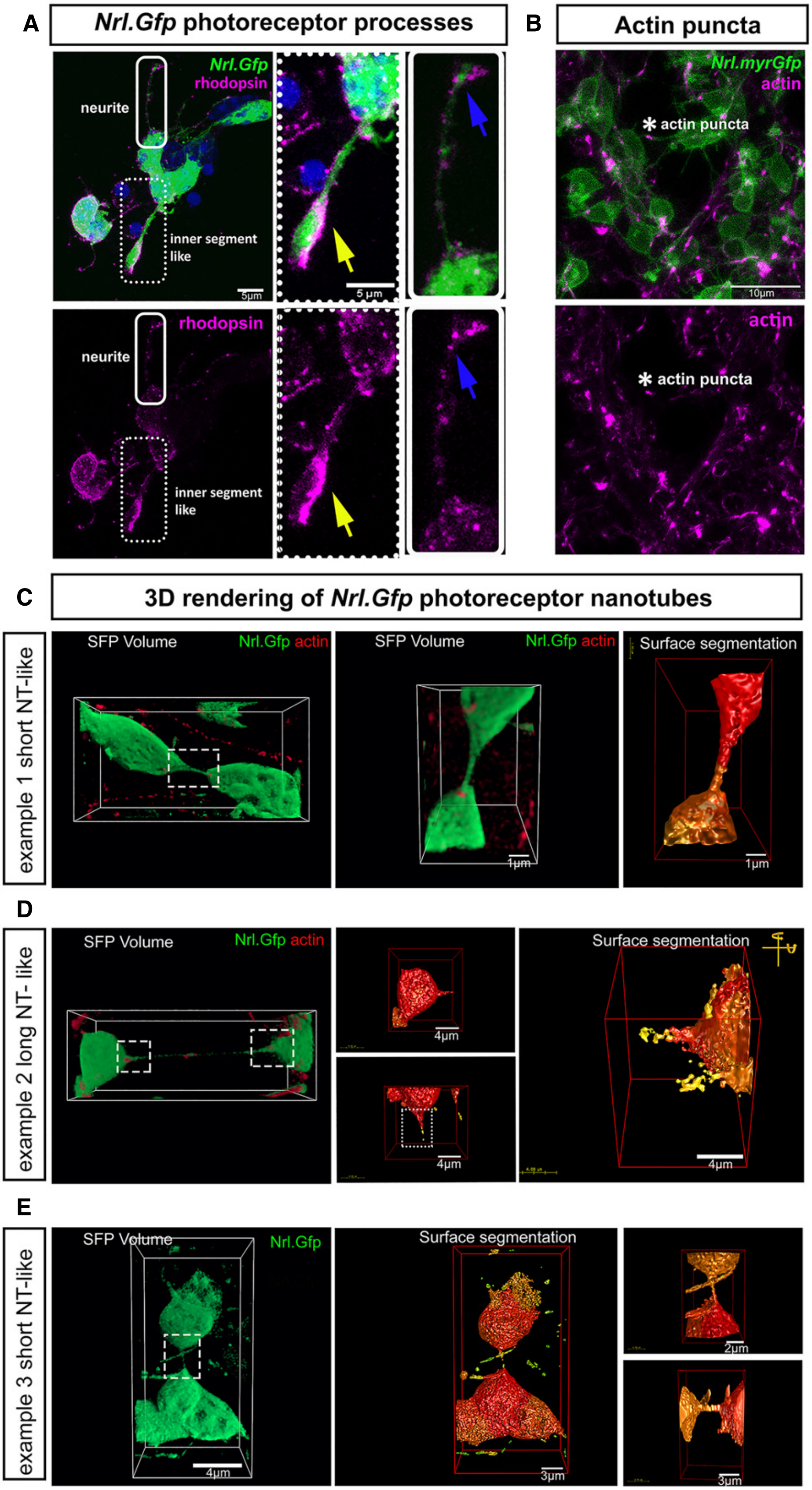

A
Nrl.Gfp photoreceptor processes
Nrl.Gfp
rhodopsin
neurite
inner segment like
5μm
5 μm
rhodopsin
neurite
inner segment like
B
Actin puncta
Nrl.myrGfp
actin
* actin puncta
10μm
actin
* actin puncta
C
3D rendering of Nrl.Gfp photoreceptor nanotubes
example 1 short NT-like
SFP Volume
Nrl.Gfp actin
SFP Volume
Nrl.Gfp actin
Surface segmentation
1μm
1μm
D
example 2 long NT- like
SFP Volume
Nrl.Gfp actin
4μm
4μm
Surface segmentation
4μm
E
example 3 short NT-like
SFP Volume
Nrl.Gfp
4μm
Surface segmentation
3μm
2μm
3μm

Figure EV2.

Figure EV3. Pharmacological disruption of actin dynamics affects photoreceptor process formation and transfer of cytoplasmic and lipid reporters between photoreceptors in culture.

A, B Flow cytometry gating strategy of either (A) co-cultures of *Nrl.Gfp* and *DsRed* cells, or (B) DiI and DiO labelled cells. In each case, panels reading *top left* to *bottom right* are: 1. Live cells selection, 2. Single events in FSC, 3. More single events in SSC, 4. Single live events in FSC/SSC, 5. $CD73^{-}$APC versus FSC, 6. $Nrl.Gfp^{+}$ vs $DsRed^{+}$ within the $CD73APC^{+}$ population (for A) or 6. DiI^{+} vs DiO^{+} within the $CD73APC^{+}$ population (for B).

C, D Representative examples of confocal imaging of P8 $Nrl.Gfp^{+/+}$ photoreceptors fixed after 48 h exposure to different concentrations of the actin polymerization inhibitors (C), Latrunculin B (50 μM, 5 μM and 0.05 μM) and (D), Cytochalasin D (20 μM, 2 μM and 0.02 μM), compared to vehicle controls (DMSO); Scale bars = 50 μm.

E, F Statistical analysis of the percentage of photoreceptors exhibiting processes following 48 h treatment with Latrunculin B (50 μM, 5 μM and 0.05 μM) or Cytochalasin D (20 μM, 2 μM and 0.02 μM), compared to vehicle control. One-way ANOVA non-parametric, two-tailed analysis, *F*-test, Kolmogorov–Smirnov, $^{*}P < 0.05$ shows significant effect of both drugs on the ability of photoreceptors to elaborate processes, compared to vehicle control; ($N = 3$ independent cultures, $n = 1$ well per condition). Graph shows mean ± SD.

G Representative MIP of $myrRFP^{+ve}/CD73^{+ve}$ photoreceptors (*red*) immunostained for LAMP1 (*green*) after 3DIC; *Circle* = $LAMP1^{+ve}$ vesicle in long ^{Ph}NTs. Scale bar = 10 μm.

© 2021 The Authors

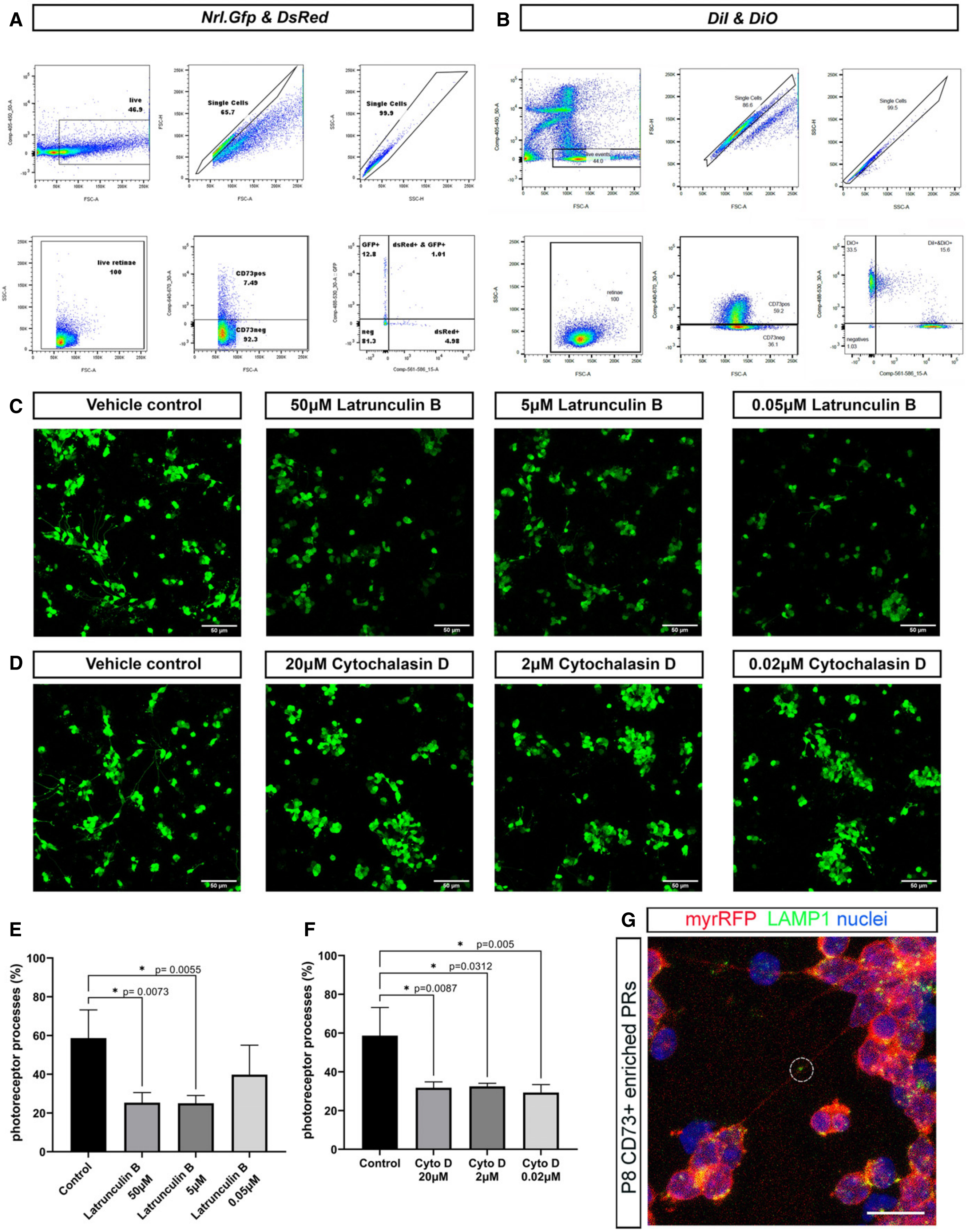
A
Nrl.Gfp & DsRed
B
Dil & DiO
C
Vehicle control
50μM Latrunculin B
5μM Latrunculin B
0.05μM Latrunculin B
D
Vehicle control
20μM Cytochalasin D
2μM Cytochalasin D
0.02μM Cytochalasin D
E
photoreceptor processes (%)
p= 0.0073
p= 0.0055
Control
Latrunculin B 50μM
Latrunculin B 5μM
Latrunculin B 0.05μM
F
photoreceptor processes (%)
p=0.0087
p=0.0312
p=0.005
Control
Cyto D 20μM
Cyto D 2μM
Cyto D 0.02μM
G
myrRFP LAMP1 nuclei
P8 CD73+ enriched PRs

Figure EV3.

Figure EV4. Transplantation of *Nrl.Gfp*$^{+/+}$ photoreceptors results in the transfer of cytoplasmic protein and some membrane-bound protein, but little or no mRNA.

A Representative MIP confocal images of *wild-type* (wt) eye cups fixed 21 days post-transplantation with P8 myrRFP^{+ve} CD73^{+ve} MACS-enriched photoreceptors. Note the presence of RFP^{+} host inner segments (*arrows*). myr-RFP (*red*), nuclei (*blue*).

B Representative MIP confocal images of *Gnat1*$^{-/-}$ eye cups fixed and stained in situ (RNAScope) with *Gfp* mRNA probe (*red*) 21 days post-transplantation with P8 *Nrl.Gfp*$^{+/+}$ photoreceptors (*green*). *Blue* = Dapi (nuclei); CM: cell mass; ONL: Outer Nuclear Layer. Asterisks denote a rare example of an integrated donor photoreceptor located within the host ONL and presenting robust staining for *Gnat1* mRNA in the inner segment.

C, D Representative MIP confocal images of (C), *Nrl.Gfp*$^{+/+}$ and (D), *Gnat1*$^{-/-}$ eye cups fixed and stained *in situ* (RNAScope) with a null probe (assay negative control, *red*), *UBC* mRNA probe (assay positive control, *red*), *Gfp* mRNA probe (biological positive control, *red*), *Gnat1* mRNA probe (biological positive control, *red*), and *Gnat1*$^{-/-}$ tissue stained with either *Gfp* mRNA probe (biological negative control, *red*) or *Gnat1* mRNA probe (biological negative control, *red*). *Nrl.Gfp*$^{+/+}$ photoreceptors (*green*), nuclei (*blue*); mRNA (*red*).

Data information: Scale bars = 20 µm.

 © 2021 The Authors

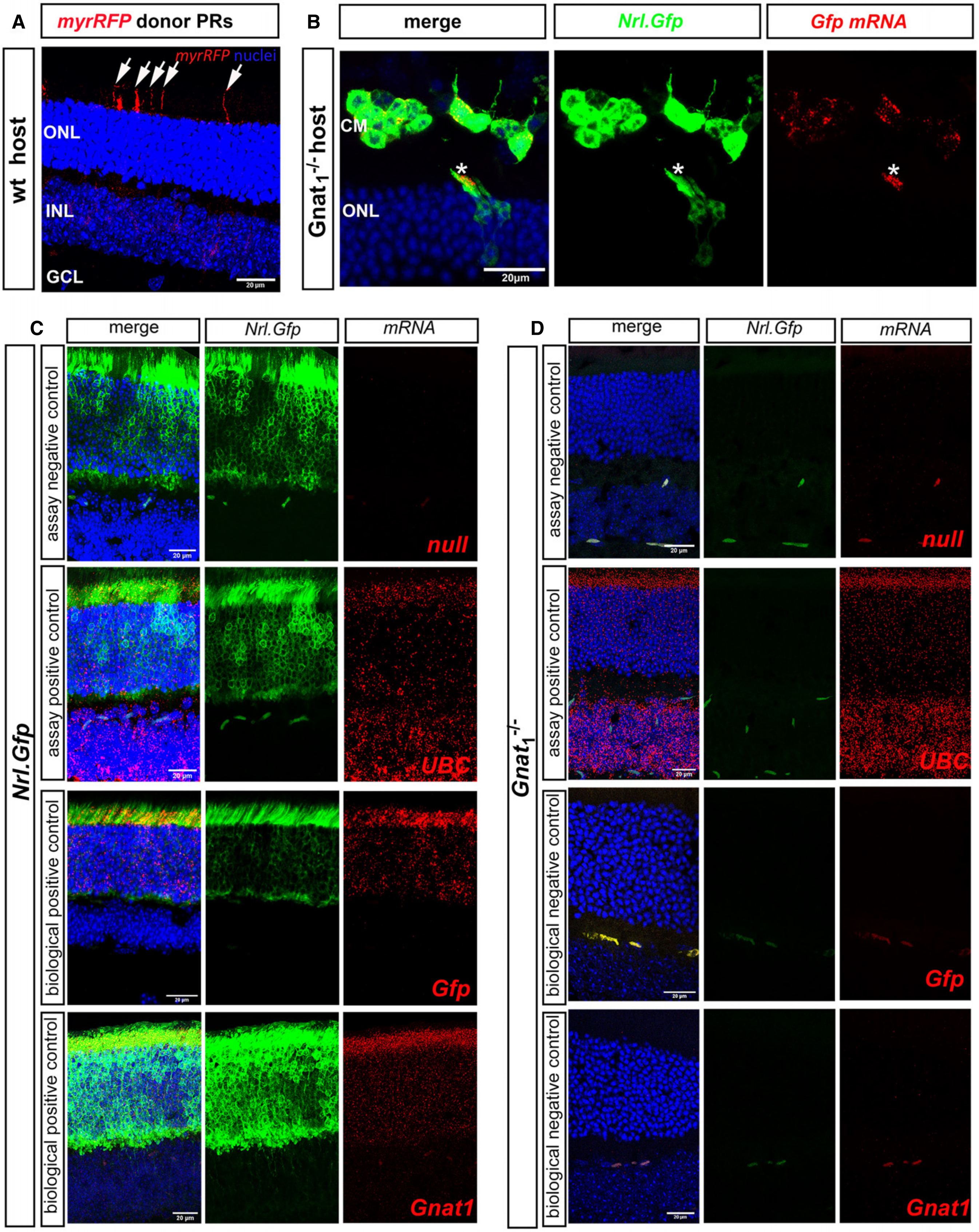

A
myrRFP donor PRs
wt host
myrRFP nuclei
ONL
INL
GCL
20 µm
B
merge
Nrl.Gfp
Gfp mRNA
Gnat1-/- host
CM
ONL
20µm
C
merge
Nrl.Gfp
mRNA
Nrl.Gfp
assay negative control
null
assay positive control
UBC
biological positive control
Gfp
biological positive control
Gnat1
D
merge
Nrl.Gfp
mRNA
Gnat1-/-
assay negative control
null
assay positive control
UBC
biological negative control
Gfp
biological negative control
Gnat1

Figure EV4.

Figure EV5. Manipulation of actin dynamics by overexpression of RhoA or DNRac1 in transplanted photoreceptors correlates with reduced GFP and rod α-transducin transfer.

A Representative Western blots of *RhoA and Rac1* expression in mixed P0–2 retinal cultures transduced with plenti-CAG-P2A.GFP, plenti-CAG-ΔNRac1-P2A.GFP or plenti-CAG-RhoA-P2A.GFP compared to β-actin and assessed after 8 DIC.

B Representative MIP confocal images of P0–2 retinal cultures transduced with plenti-CAG-P2A.GFP (*green*) or plenti-CAG-RhoA-P2A.GFP (*green*) or plenti-CAG-ΔNRac1-P2A.GFP (*green*), fixed and stained with F-actin (*red*) ($N = 2$ independent cultures, $n = 4$ wells per condition); Scale bars = 20 μm.

C Quantification and statistical analysis of the effect of overexpression of RhoA and ΔNRac1, versus control, on actin in P0–2 retinal cultures at 7DIC. Effect assessed by measurement of mean intensities (Image J) of F-actin plaques (*red*) in GFP^+ cells (*green*), normalized to GFP^+ cell number; control 2.6 ± 0.7; RhoA 13.9 ± 2.1, ΔNRac 4.5 ± 1.3. Mean ± SD. One-way ANOVA non-parametric two-tail, Kruskal–Wallis post-test ***$P < 0.001$ ($N = 2$ independent cultures, $n = 4$ wells per condition).

D Representative tile-scan images of *Gnat1*$^{-/-}$ eyes transplanted with P2 retinal cells transduced with *plenti-CAG-P2A.GFP* (*green*) or *plenti-CAG-RhoA-P2A.GFP* (*green*) or *plenti-CAG-ΔNRac1-P2A.GFP (green)*. *Green* = GFP, *blue* = Dapi (nuclei); Scale bars = 50 μm.

E–G Representative MIP images of *Gnat1*$^{-/-}$ eyes transplanted with P2 retinal cells transduced with (E), *plenti-CAG-P2A.GFP* (*green*) or (F), *plenti-CAG-RhoA-P2A.GFP* (*green*) or (G), *plenti-CAG-ΔNRac1-P2A.GFP (green)*. Immunostaining for Rod α-transducin indicates that inhibition of actin polymerization impairs transfer of Rod α-transducin alongside that of GFP. *Green* = GFP, *blue* = Dapi (nuclei), *red* = Rod α-transducin; Scale bars = 50 μm.

Data information: RPE = retinal pigment epithelium, CM = cell mass, ONL = outer nuclear layer, INL = inner nuclear layer, GCL = ganglion cell layer. All eyes were fixed and examined 21 days post-transplantation.

 © 2021 The Authors

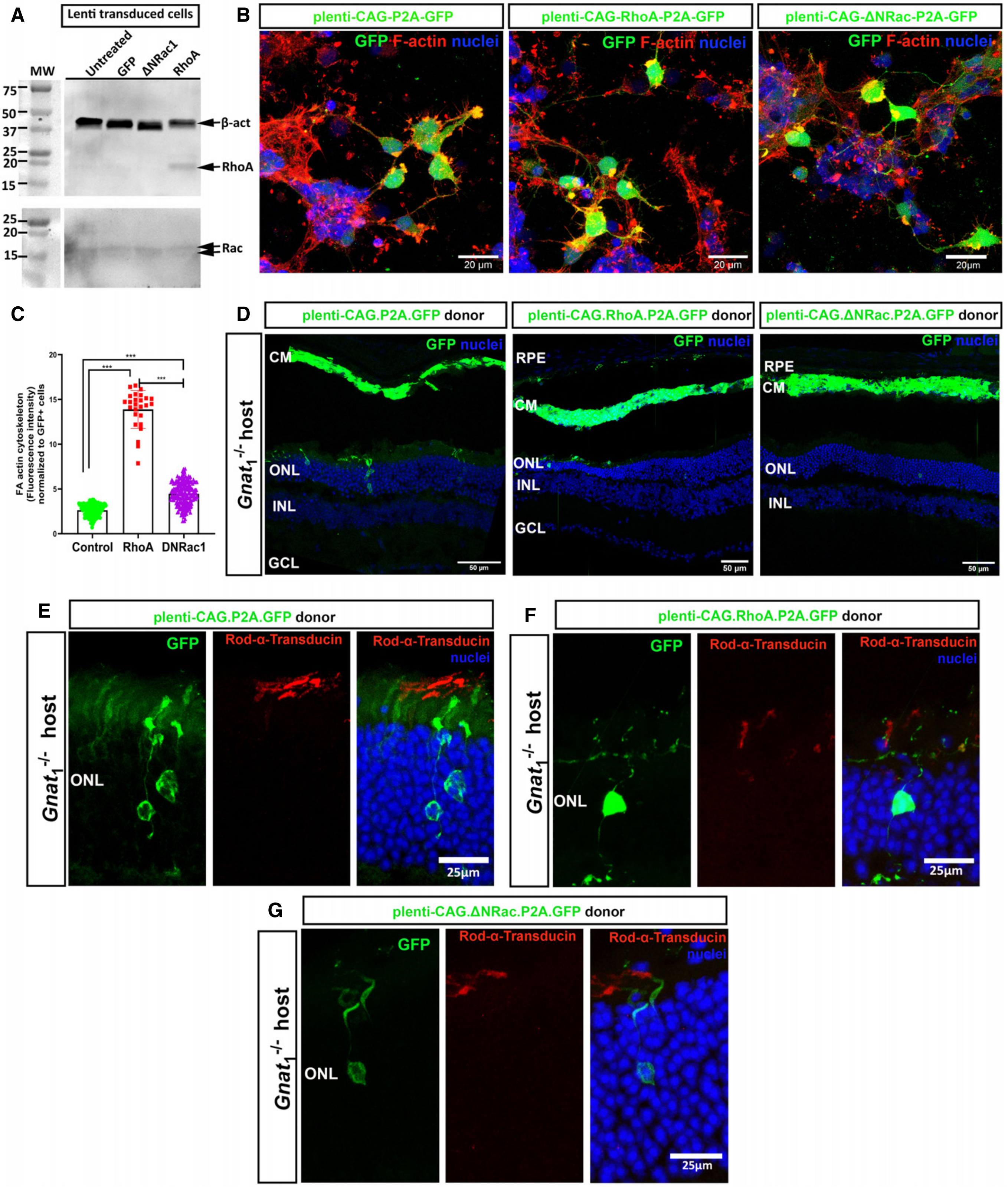
A
Lenti transduced cells
MW
Untreated
GFP
ΔNRac1
RhoA
75
50
37
25
20
15
β-act
RhoA
Rac
B
plenti-CAG-P2A-GFP
plenti-CAG-RhoA-P2A-GFP
plenti-CAG-ΔNRac-P2A-GFP
GFP F-actin nuclei
20 μm
C
FA actin cytoskeleton (Fluorescence intensity) normalized to GFP+ cells
***
Control
RhoA
DNRac1
D
plenti-CAG.P2A.GFP donor
plenti-CAG.RhoA.P2A.GFP donor
plenti-CAG.ΔNRac.P2A.GFP donor
Gnat1 -/- host
GFP nuclei
CM
RPE
ONL
INL
GCL
50 μm
E
plenti-CAG.P2A.GFP donor
GFP
Rod-α-Transducin
Rod-α-Transducin nuclei
25μm
F
plenti-CAG.RhoA.P2A.GFP donor
G
plenti-CAG.ΔNRac.P2A.GFP donor

Figure EV5.
