# Supplementary material for: Nanotube‐like processes facilitate material transfer between photoreceptors
Source: EMBO Rep. 2021 Sep 8;22(11):e53732. doi: 10.15252/embr.202153732 (PMC8567251; doi:10.15252/embr.202153732)
Supplement: Supplementary file 3 — Movie EV1 [file EMBR-22-e53732-s005.zip › 107292R_Movie_EV1/107292R_Movie_EV1_Legend.docx]

**Movie EV 1. Photoreceptors form nanotube-like processes in culture.**

3D deconvolved surface per volume images from live imaging of *Nrl.Gfp^+/+^* (*green*) P8 photoreceptors in culture showing two cells (*dashed circles*) connected with a NT-like process (*dashed box*), herein termed ^Ph^NTs, followed by digitally enhanced 3D surface segmentation of the same ^Ph^NT (*dashed box*) in 360° rotation.
