# Supplementary material for: Nanotube‐like processes facilitate material transfer between photoreceptors
Source: EMBO Rep. 2021 Sep 8;22(11):e53732. doi: 10.15252/embr.202153732 (PMC8567251; doi:10.15252/embr.202153732)
Supplement: Supplementary file 4 — Movie EV2 [file EMBR-22-e53732-s007.zip › 107292R_Movie_EV2/107292R_Movie_EV2_Legend.docx]

**Movie EV 2. Actin labelling in short thin ^Ph^NTs forming between photoreceptors in culture.**

3D deconvolved surface per volume images from live imaging of *Nrl.Gfp^+/+^* (*green*) P8 photoreceptors show two connected cells with a thin ^Ph^NT process (*dashed box*). First part of the movie shows SiR-actin surface rendering (*red*) versus cytoplasm volume (*green*) in 360º rotation. Second part of the movie shows segmentation of the cell cytoplasm (GFP) in 360º rotation.
