# Supplementary material for: Nanotube‐like processes facilitate material transfer between photoreceptors
Source: EMBO Rep. 2021 Sep 8;22(11):e53732. doi: 10.15252/embr.202153732 (PMC8567251; doi:10.15252/embr.202153732)
Supplement: Supplementary file 5 — Movie EV3 [file EMBR-22-e53732-s001.zip › 107292R_Movie_EV3/107292R_Movie_EV3_Legend.docx]

**Movie EV 3. Tubulin labelling in short thick ^Ph^NTs forming between photoreceptors in culture.**

3D deconvolved volume movie from live imaging of *Nrl.Gfp^+/+^* (*green*) P8 photoreceptorsshows two connected cells with a thick, SiR-tubulin-rich (*red*) ^Ph^NT. First part of movie shows volume localization in xyz coordinates with a heatmap scale in 360º rotation. Second part shows segmentation of the cell cytoplasm segmentation in 360º rotation.
