# Supplementary material for: Nanotube‐like processes facilitate material transfer between photoreceptors
Source: EMBO Rep. 2021 Sep 8;22(11):e53732. doi: 10.15252/embr.202153732 (PMC8567251; doi:10.15252/embr.202153732)
Supplement: Supplementary file 6 — Movie EV4 [file EMBR-22-e53732-s008.zip › 107292R_Movie_EV4/107292R_Movie_EV4.docx]

**Movie EV 4. FRAP of cGFP in ^Ph^NT-connected photoreceptor shows robust fluorescent recovery of GFP fluorescence.**

Representative example from live imaging of *Nrl.Gfp^+/+^* (*green)* P8 photoreceptors showing xyt of FRAP of cGFP in whole cell cytoplasm of a ^Ph^NT-connected photoreceptor (*dashed circle* = bleaching ROI of acceptor cell) versus the connected donor cell (labelled *donor*), alongside non-bleached reference cells. GFP fluorescence recovers over time in the connected bleached cell. Movie shows 5 frames prior photobleaching followed by a post-bleaching recovery period of 210 s. Frame rate = 0.826 s.
