# Supplementary material for: Nanotube‐like processes facilitate material transfer between photoreceptors
Source: EMBO Rep. 2021 Sep 8;22(11):e53732. doi: 10.15252/embr.202153732 (PMC8567251; doi:10.15252/embr.202153732)
Supplement: Supplementary file 7 — Movie EV5 [file EMBR-22-e53732-s002.zip › 107292R_Movie_EV5/107292R_Movie_EV5_Legend.docx]

**Movie EV 5. ^Ph^NT-connected photoreceptors can occasionally exchange lysosomes.**

Example of lysosomal transfer between ^Ph^NT-connected photoreceptors. Movie shows live imaging of *Nrl.Gfp^+/+^* (*green*) P8 photoreceptors, showing a 3D deconvolved surface (SiR-Lyso; *red*) versus volume (cytoplasm, *green*) time-lapse (60 mins duration). The connected cells are shown in both xy and yz 180º rotations. Bottom movie indicates the 3D surface unidirectional movement of lysosomes across time within the connected cell borders. Frame rate = 3 mins.
