# Supplementary material for: Nanotube‐like processes facilitate material transfer between photoreceptors
Source: EMBO Rep. 2021 Sep 8;22(11):e53732. doi: 10.15252/embr.202153732 (PMC8567251; doi:10.15252/embr.202153732)
Supplement: Supplementary file 8 — Movie EV6 [file EMBR-22-e53732-s010.zip › 107292R_Movie_EV6/107292R_Movie_EV6_Legend.docx]

**Movie EV 6. ^Ph^NT-connected photoreceptors can occasionally exchange mitochondria.**

Example of mitochondrial transfer between ^Ph^NT-connected photoreceptors. Movie shows live imaging of *Nrl.Gfp^+/+^* (*green*) P8 photoreceptors showing a 3D deconvolved surface (mitochondria, MitoTracker, *red*) versus volume (cytoplasm, *green*) time-lapse (30 mins). The connected cells are shown in both xy and yz 180º rotations. Bottom movie indicates the 3D surface unidirectional movement of mitochondria across time within the connected cell borders. In contrast, mitochondria appear to move bidirectionally within the segment-like region. Frame rate = 3 mins.
