# Supplementary material for: Nanotube‐like processes facilitate material transfer between photoreceptors
Source: EMBO Rep. 2021 Sep 8;22(11):e53732. doi: 10.15252/embr.202153732 (PMC8567251; doi:10.15252/embr.202153732)
Supplement: Supplementary file 9 — Movie EV7 [file EMBR-22-e53732-s004.zip › 107292R_Movie_EV7/107292R_Movie_EV7_Legend.docx]

**Movie EV 7. Transplanted donor and host photoreceptors form ^Ph^NT-like processes in vivo facilitating molecular cytoplasmic exchange.**

Representative image of 3D reconstruction followed by segmentation of *wildtype*retina transplanted with P8 *Nrl.Gfp^+/+^* CD73+ MACS-enriched photoreceptors; *dashed box* indicates ROI subjected to 3D deconvoluted surface reconstruction, which shows ^Ph^NT-like connection at the level of host inner segment region between a GFP^+ve^ donor photoreceptor and a host photoreceptor, which has received cGFP. *Green* = GFP, *blue* = nuclei; Data generated with HyVolution software.
