# Supplementary material for: Nanotube‐like processes facilitate material transfer between photoreceptors
Source: EMBO Rep. 2021 Sep 8;22(11):e53732. doi: 10.15252/embr.202153732 (PMC8567251; doi:10.15252/embr.202153732)
Supplement: Supplementary file 10 [file EMBR-22-e53732-s006.pdf]

EMBO reports

# Nanotube-like processes facilitate material transfer between photoreceptors

Aikaterini Kalargyrou, Mark Basche, Aura Hare, Emma West, Alexander Smith, Robin Ali, and Rachael Pearson
**DOI: 10.15252/embr.202153732**

*Corresponding author(s): Rachael Pearson (rachael.pearson@kcl.ac.uk) , Aikaterini Kalargyrou (aikaterini.kalargyrou@kcl.ac.uk)*



## Review Timeline:

| | |
|---|---|
| Transfer Date: | 31st Jul 21 |
| Editorial Decision: | 4th Aug 21 |
| Revision Received: | 16th Aug 21 |
| Accepted: | 20th Aug 21 |

*Editor: Deniz Senyilmaz Tiebe*


## Transaction Report: This manuscript was transferred to EMBO reports following peer review at The EMBO Journal.

(Note: With the exception of the correction of typographical or spelling errors that could be a source of ambiguity, letters and reports are not edited. Depending on transfer agreements, referee reports obtained elsewhere may or may not be included in this compilation. Referee reports are anonymous unless the Referee chooses to sign their reports.)

## 1st Editorial Decision 4th Aug 2021

Dear Dr. Pearson,

Thank you for transferring your manuscript to EMBO Reports, which was previously reviewed at The EMBO Journal. Having looked at everything, I would like to invite you to submit a manuscript with minor revisions as outlined below.

• We note that there are currently two manuscript files. Please upload the most up-to-date version in the word format.
• Please perform the textual changes/amendments you outlined in your point-by-point response with track changes on.
• We note that the Appendix is missing a Table of Contents, and the figure legend is in the Article file. The textual callout needs to be corrected to 'Appendix Figure S1' and the panel callouts needs to be added in.
• We note that the movies are currently missing. The movies need to be uploaded and ZIPped with their legend. The legends should be removed from the Article file.
• We note that Emma West is currently missing from the Author Contributions section.
• We realize that Fig EV1C is currently not called out in the text.
• As per our format requirements, the size of synopsis image 550px wide and 300-600px high. The current synopsis image gets difficult to read/understand when resized. Please provide a simplified synopsis image with larger labels.
• Our production/data editors have asked you to clarify several points in the figure legends (see attached document). Please incorporate these changes in the attached word document and return it with track changes activated.

Thank you again for giving us to consider your manuscript for EMBO Reports, I look forward to your minor revision.

Kind regards,

Deniz Senyilmaz Tiebe

--
Deniz Senyilmaz Tiebe, PhD
Editor
EMBO Reports

**Response to specific points raised by Reviewer 2**

1.) Major: The major point of contention remains in that the current manuscript still does not depict any evidence that the nanotubes mediate "intercellular communication" between photoreceptors. In their response, the authors state that they believe that "intercellular communication" does not require the material being exchanged to be functionally relevant. This makes no sense and would be incredibly misleading to the readers. The authors point out that several previous papers describe nanotubes as mediating "intercellular communication". In some cases, this is warranted (e.g. demonstration of active propagation of Ca2+ signals within nanotubes). In many cases, this is absolutely not warranted (e.g. when the authors merely demonstrate the transfer of a GFP reporter). Using the overinterpretations of other papers as justification to repeat a similar overinterpretation is not appropriate.

We accept the reviewer's concerns. We have revised the manuscript to remove the use of the term "intercellular communication" beyond the introduction and discussion, where nanotubes and EVs are discussed in broader context. We have replaced this with our original term "material transfer".

2.) Major: I must stand by the point that there is no evidence in this manuscript that extracellular vesicles (EVs) mediate intercellular communication between photoreceptors and Muller glial cells. While photoreceptors may contain multivesicular bodies, this is not a sufficient evidence that photoreceptors produce exosomes in vivo and, in fact, the authors recognize this weakness of their argument. While Muller glia can take up injected EVs (or apparently any type of membrane material as shown in a revised Figure 2), there is no evidence that this is a normally occurring phenomenon. In fact, the authors admit that in their own chimera experiments, they do not observe any such event. Yet, they claim that: "we show for the first time that sensory neurons can engage in intercellular communication in vivo by both EVs and open-end NT-like processes, each targeting different cell populations." Please revise your conclusions here and elsewhere accordingly.

As noted above, we have amended the manuscript to remove any assertions regarding intercellular communication and have revised the descriptions and conclusions around photoreceptor derived EVs to state that we observed their uptake by Muller glia, and not photoreceptors, following injection in vivo and that this shows EVs are not the mediators of material transfer between photoreceptors.

3.) Major: Please show the images for the UV-treated transplanted photoreceptors (Figs. 2G and 5E). This is essential to control for the non-specific Muller glia uptake of membranous debris.

It is unfortunate that this oversight was not picked up by any of the reviewers in the first submission, as we had this information available. Full quantification of these experiments was included in two figures and no transfer was seen in any UV-treated transplantations, but we agree that inclusion of an image would support this quantification. We have included an additional image of UV-treated Nrl.Cre +/- transplants in Expanded View Fig. EV1e and of UV-treated Nrl.Gfp x myrRFP+ve transplants in Appendix Figure 2 (see attached revised MS).

4.) Relatively major: The authors insist on the novelty of their findings based on the fact that the 2016 Stem Cells paper by Ortin-Martinez et al. did not use the word "nanotubes" while clearly showing physical connections that appear as long, cylindrical tubes between transplanted cells and host photoreceptors (Fig 3B in that paper). I recognize that the current study provides by far a larger body of work definitively identifying these structures as nanotubes, yet the authors should properly cite the work by Ortin-Martinez et al. in this specific context as providing early evidence that the material transfer may occur through a nanotube-mediated mechanism.

We consider this assertion to be unfair, given the recent acceptance of the co-submitted manuscript by Ortin-Martinez/Wallace on exactly the same topic. Both groups show very similar data, and both conclude the role of nanotube-like structures mediating material exchange between photoreceptors. To say that one, but not the other, is novel raises significant concerns.

We are happy to include a new sentence that makes specific reference to findings from not only Ortin-Martinez and colleagues, but also other papers at that time, that there were indications that material transfer might involve a physical interaction (page 9, start of second paragraph). However, it must be emphasised that this is different to actually elucidating the cellular mechanisms, as both we, and Ortin-Martinez and colleagues themselves, have now gone on to show. Indeed, Ortin-Martinez and colleagues concluded in their 2016 paper that "Donor/host DNA and mitochondrial transfer, and intercellular exchange via microvesicle/exovesicle GFP, tunneling nanotubes have been described in various systems, offering *prospective* cellular transfer mechanisms."

5.) **Minor:** As another over-emphasis of the "communicative" capacity of EVs, the authors state that: "Previously regarded as part of the cell's 'garbage disposal system', EVs are now recognized as lipid-encapsulated carriers of bioactive material, including cytosolic and membrane proteins and genetic material, which can alter acceptor cell function in culture and in vivo". While EVs can indeed have functional significance, they are not always "carriers of bioactive material". Please soften this line.

We have revised the sentence as requested (see page 3, start of first paragraph), which now reads:

"Previously regarded as part of the cell's 'garbage disposal system', EVs can carry cytosolic and membrane proteins and potentially even genetic material, which have been reported to alter acceptor cell function in culture and in vivo".

6.) **Minor:** Actin does not seem to be involved in material transfer through nanotubes while being important for nanotube formation. Indeed, the transfer was decreased by ~2-fold in the presence of actin inhibitors, which exactly corresponds to the observed ~2-fold reduction in nanotube formation. This should be made more clear in the text.

Thank you for pointing out this additional observation. We have added a sentence stating this to the relevant section (page 13, end of paragraph 1).

**7.) Minor:**
Figure 1g. Scale bars for top panels should all be the same. No comparison between transducin, recoverin, and rhodopsin can be made as currently shown.
We have amended the scale bars in the recoverin, rhodopsin and Crx graphs to include the split scale, as used for transducin – see Fig. 1g.

Fig. 6J. Please change P2A.Rho.GFP to P2A.RhoA.GFP.
We have amended the typo in Fig. 6J.

Fig. 2A. The RPE is transduced with AAV-Nrl.Cre; is this actually AAV-CMV.Cre?
No, the virus used was *AAV-Nrl.Cre*. We suggest the RPE recombination (also noted in the figure legend) may arise from either non-specific expression of Cre within the RPE, or possibly recombination resulting from uptake of small amounts of Cre protein. We have added a sentence to the Results to explain this (page 8, middle of first paragraph).

## 1st Revision - Editorial Decision 20th Aug 2021

Dear Rachael,

Thank you for submitting your revised manuscript. I have now looked at everything and all is fine. Therefore, I am very pleased to accept your manuscript for publication in EMBO Reports.

Congratulations on a nice work!

Kind regards,

Deniz
--
Deniz Senyilmaz Tiebe, PhD
Editor
EMBO Reports

--

At the end of this email I include important information about how to proceed. Please ensure that you take the time to read the information and complete and return the necessary forms to allow us to publish your manuscript as quickly as possible.

As part of the EMBO publication's Transparent Editorial Process, EMBO reports publishes online a Review Process File to accompany accepted manuscripts. As you are aware, this File will be published in conjunction with your paper and will include the referee reports, your point-by-point response and all pertinent correspondence relating to the manuscript.

If you do NOT want this File to be published, please inform the editorial office within 2 days, if you have not done so already, otherwise the File will be published by default [contact: emboreports@embo.org]. If you do opt out, the Review Process File link will point to the following statement: "No Review Process File is available with this article, as the authors have chosen not to make the review process public in this case."

Should you be planning a Press Release on your article, please get in contact with emboreports@wiley.com as early as possible, in order to coordinate publication and release dates.

Thank you again for your contribution to EMBO reports and congratulations on a successful publication. Please consider us again in the future for your most exciting work.

*****************************************************************************

THINGS TO DO NOW:

You will receive proofs by e-mail approximately 2-3 weeks after all relevant files have been sent to our Production Office; you should return your corrections within 2 days of receiving the proofs.

Please inform us if there is likely to be any difficulty in reaching you at the above address at that time. Failure to meet our deadlines may result in a delay of publication, or publication without your

corrections.

All further communications concerning your paper should quote reference number EMBOR-2021-53732V2 and be addressed to emboreports@wiley.com.

Should you be planning a Press Release on your article, please get in contact with emboreports@wiley.com as early as possible, in order to coordinate publication and release dates.

EMBO PRESS

YOU MUST COMPLETE ALL CELLS WITH A PINK BACKGROUND ↓

PLEASE NOTE THAT THIS CHECKLIST WILL BE PUBLISHED ALONGSIDE YOUR PAPER

Corresponding Author Name: Aikaterini Kalargyrou & Rachel Pearson

Journal Submitted to: EMBO Reports
Manuscript Number: EMBOR_2021_53732

## Reporting Checklist For Life Sciences Articles (Rev. June 2017)

This checklist is used to ensure good reporting standards and to improve the reproducibility of published results. These guidelines are consistent with the Principles and Guidelines for Reporting Preclinical Research issued by the NIH in 2014. Please follow the journal's authorship guidelines in preparing your manuscript.

### A- Figures
### 1. Data
### The data shown in figures should satisfy the following conditions:

- the data were obtained and processed according to the field's best practice and are presented to reflect the results of the experiments in an accurate and unbiased manner.
- figure panels include only data points, measurements or observations that can be compared to each other in a scientifically meaningful way.
- graphs include clearly labeled error bars for independent experiments and sample sizes. Unless justified, error bars should not be shown for technical replicates.
- if n< 5, the individual data points from each experiment should be plotted and any statistical test employed should be justified
- Source Data should be included to report the data underlying graphs. Please follow the guidelines set out in the author ship guidelines on Data Presentation.

### 2. Captions

### Each figure caption should contain the following information, for each panel where they are relevant:

- a specification of the experimental system investigated (eg cell line, species name).
- the assay(s) and method(s) used to carry out the reported observations and measurements
- an explicit mention of the biological and chemical entity(ies) that are being measured.
- an explicit mention of the biological and chemical entity(ies) that are altered/varied/perturbed in a controlled manner.

- the exact sample size (n) for each experimental group/condition, given as a number, not a range;
- a description of the sample collection allowing the reader to understand whether the samples represent technical or biological replicates (including how many animals, litters, cultures, etc.).
- a statement of how many times the experiment shown was independently replicated in the laboratory.
- definitions of statistical methods and measures:
  - common tests, such as t-test (please specify whether paired vs. unpaired), simple χ2 tests, Wilcoxon and Mann-Whitney tests, can be unambiguously identified by name only, but more complex techniques should be described in the methods section;
  - are tests one-sided or two-sided?
  - are there adjustments for multiple comparisons?
  - exact statistical test results, e.g., P values = x but not P values < x;
  - definition of 'center values' as median or average;
  - definition of error bars as s.d. or s.e.m.

Any descriptions too long for the figure legend should be included in the methods section and/or with the source data.

**In the pink boxes below, please ensure that the answers to the following questions are reported in the manuscript itself. Every question should be answered. If the question is not relevant to your research, please write NA (non applicable). We encourage you to include a specific subsection in the methods section for statistics, reagents, animal models and human subjects.**

### USEFUL LINKS FOR COMPLETING THIS FORM

http://www.antibodypedia.com
http://1degreebio.org
http://www.equator-network.org/reporting-guidelines/improving-bioscience-research-repor
http://grants.nih.gov/grants/olaw/olaw.htm
http://www.mrc.ac.uk/Ourresearch/Ethicsresearchguidance/Useofanimals/index.htm
http://ClinicalTrials.gov
http://www.consort-statement.org
http://www.consort-statement.org/checklists/view/32-consort/66-title
http://www.equator-network.org/reporting-guidelines/reporting-recommendations-for-tumo
http://datadryad.org
http://figshare.com
http://www.ncbi.nlm.nih.gov/gap
http://www.ebi.ac.uk/ega
http://biomodels.net/
http://biomodels.net/miriam/
http://jjj.biochem.sun.ac.za
http://oba.od.nih.gov/biosecurity/biosecurity_documents.html
http://www.selectagents.gov/

### B- Statistics and general methods

Please fill out these boxes ↓ (Do not worry if you cannot see all your text once you press return)

| | |
|---|---|
| 1.a. How was the sample size chosen to ensure adequate power to detect a pre-specified effect size? | Estimates of group sizes are based on variance estabished in pilot data (cell culture) or published data (transplantation experiments), as appropriate, setting a power of 0.8 and significance level of 0.05. All data is shown as means ± standard deviation (S.D.) of the mean, unless otherwise indicated. Generation of plots, curve fitting and statistical analyses were performed using GraphPad Prism 8 for Windows Version 10 (GraphPad Software Inc). Statistical significance was assessed using non-parametric one-way analysis of variance (ANOVA) with Dunnett's multiple comparison post hoc test (compared all groups against control group) or Bonferroni multiple comparisons post hoc test (compared all groups or selected groups) or as stated in figure legends |
| 1.b. For animal studies, include a statement about sample size estimate even if no statistical methods were used. | Animal sample sizes were determined from power calculations based on the variance observed in transplantation experiments as stated in prior publications (MacLaren et al., 2006; Pearson et al., 2012, 2016) |
| 2. Describe inclusion/exclusion criteria if samples or animals were excluded from the analysis. Were the criteria pre-established? | In the rare cases of exclusion of samples from statistical analysis an Outlier Exclusion Method was selected via GraphPad prism in conjuction with normality tests followed up by statistical analysis test and post-hoc. |
| 3. Were any steps taken to minimize the effects of subjective bias when allocating animals/samples to treatment (e.g. randomization procedure)? If yes, please describe. | Block randomization procedure was selected. In experiments with 3 or more different treatment groups including appropriate controls there was a block size of 4 or more animals (in vivo), or 4 or more culture wells (in vitro) per treatment group within each experimental trial, followed up by at least 4 independent experimental trials. Block randomization was used in conjunction with double blind testing to ensure minimization of bias. |
| For animal studies, include a statement about randomization even if no randomization was used. | Randomization took place via selection animals deriving of 3 or more independent litters, with 1:1 female to male ratio. |
| 4.a. Were any steps taken to minimize the effects of subjective bias during group allocation or/and when assessing results (e.g. blinding of the investigator)? If yes please describe. | Animals, culture wells etc were allocated at random to any given test condition. Subjective bias was minimized via Double Blind Testing. In each experiment that required treatment, animals or cultures were given a code name by a second user and original treatments were revealed only after analysis. For more detail please see answer in Q3. |
| 4.b. For animal studies, include a statement about blinding even if no blinding was done | For animal studies block randomization in conjuction to double blind testing took place by assigning code numbers to animals prior to assessment. Treatment materials such as drugs/visues etc and vehicle controls were randomised and assigned a code name by an independent observer. |
| 5. For every figure, are statistical tests justified as appropriate? | Statistical tests are stated in all relevant figures. Appropriate statistical tests were selected by first assessing Normality of the sample and selection of the appropriate statisical test. Generation of plots, curve fitting and statistical analyses were performed using GraphPad Prism 8 for Windows Version 10 (GraphPad Software Inc). |
| Do the data meet the assumptions of the tests (e.g., normal distribution)? Describe any methods used to assess it. | A test of Normality was performed on all data sets prior to selecting the appropriate parametric or non parametric statistical test using GraphPad Prism 8 for Windows Version 10 (GraphPad Software Inc). |

| | |
|---|---|
| Is there an estimate of variation within each group of data? | Statistical analyses were performed using non-parametric one-way analysis of variance (ANOVA) with Dunnett's multiple comparison post hoc test (compared all groups against control group) or Bonferroni multiple comparisons post hoc test (compared all groups or selected groups) or as stated in figure legends. All data is stated as mean +/- S.D. or S.E.M as appropriate |
| Is the variance similar between the groups that are being statistically compared? | Yes |

## C- Reagents

| | |
|---|---|
| 6. To show that antibodies were profiled for use in the system under study (assay and species), provide a citation, catalog number and/or clone number, supplementary information or reference to an antibody validation profile. e.g., Antibodypedia (see link list at top right), 1DegreeBio (see link list at top right). | Primary antibodies used; Anti-LAMP1 (rat monoclonal, [1D4B] (ab25245), Abcam), anti-Gat1 (rabbit polyclonal, K-20, SC-389 Santa Cruiz), anti-Rhodopsin (mouse, O4886, Sigma), anti-GFP, (goat polyclonal,ab6673, Abcam), anti- Cre recombinase (mouse, MAB3120, Millipore) Primary anti-mouse-CD73 APC-conjugated antibody (130-103-052, Miltenyl), RhoA, (67B9) Rabbit mAb #2117 Cell Signaling; Rac, ARC03-S, Cytoskeleton Inc; beta actin; A2228, Sigma |
| 7. Identify the source of cell lines and report if they were recently authenticated (e.g., by STR profiling) and tested for mycoplasma contamination. | No cell lines were used for this research. Primary photoreceptors were obtained from P0-P8 postnatal day murine pups. |

* for all hyperlinks, please see the table at the top right of the document

## D- Animal Models

| | |
|---|---|
| 8. Report species, strain, gender, age of animals and genetic modification status where applicable. Please detail housing and husbandry conditions and the source of animals. | All animal lines; C57Bl/6J (wt) (Harlan), Nrl.gfp (kind gift of A. Swaroop, University of Michigan, USA)1, Ai9(RCL-tdT) or TdTomato2 Gt(ROSA)26Sortm4(ACTB-tdTomato,-EGFP)Luo or "mTmG" or "myrRfp",3 Gnat1tm1Clma or Gnat1-/- (kind gift of J. Lem, Tufts University School of Medicine, USA)4 were kept as homozygotes with the exception of C57BL/6J-Tg(Nrl-cre)1Smgc/J or Nrl-Cre5, which were maintained as hemizygotes, due to lethality issues. Mice were maintained in the animal facility at University College London. All experiments have been conducted in accordance with the Policies on the Use of Animals and Humans in Neuroscience Research, revised and approved by the ARVO Statement for Use of Animals in the Ophthalmic Research, under the regulation of the UK Home Office Animals (Scientific Procedures) Act 1986. Briefly, rodents were maintained on a standard 12/12-hour light dark cycle, housed in same sex groups or sustained breeding pairs wherever possible and provided with fresh bedding and nesting material and food and water ad libitum. Age of host recepient mice for transplantation experiments was between 6 to 8 weeks old, wherease for the purposes of donor cells for transplantations or cell cultures pups of age between p0-p8 postnatal day. |
| 9. For experiments involving live vertebrates, include a statement of compliance with ethical regulations and identify the committee(s) approving the experiments. | All experiments have been conducted in accordance with the Policies on the Use of Animals and Humans in Neuroscience Research, revised and approved by the ARVO Statement for Use of Animals in the Ophthalmic Research, under the regulation of the UK Home Office Animals (Scientific Procedures) Act 1986. |
| 10. We recommend consulting the ARRIVE guidelines (see link list at top right) (PLoS Biol. 8(6), e1000412, 2010) to ensure that other relevant aspects of animal studies are adequately reported. See author guidelines, under 'Reporting Guidelines'. See also: NIH (see link list at top right) and MRC (see link list at top right) recommendations. Please confirm compliance. | Corresponding authors confirm compliance |

## E- Human Subjects

| | |
|---|---|
| 11. Identify the committee(s) approving the study protocol. | N/A |
| 12. Include a statement confirming that informed consent was obtained from all subjects and that the experiments conformed to the principles set out in the WMA Declaration of Helsinki and the Department of Health and Human Services Belmont Report. | N/A |
| 13. For publication of patient photos, include a statement confirming that consent to publish was obtained. | N/A |
| 14. Report any restrictions on the availability (and/or on the use) of human data or samples. | N/A |
| 15. Report the clinical trial registration number (at ClinicalTrials.gov or equivalent), where applicable. | N/A |
| 16. For phase II and III randomized controlled trials, please refer to the CONSORT flow diagram (see link list at top right) and submit the CONSORT checklist (see link list at top right) with your submission. See author guidelines, under 'Reporting Guidelines'. Please confirm you have submitted this list. | N/A |
| 17. For tumor marker prognostic studies, we recommend that you follow the REMARK reporting guidelines (see link list at top right). See author guidelines, under 'Reporting Guidelines'. Please confirm you have followed these guidelines. | N/A |

## F- Data Accessibility

| | |
|---|---|
| 18: Provide a "Data Availability" section at the end of the Materials & Methods, listing the accession codes for data generated in this study and deposited in a public database (e.g. RNA-Seq data: Gene Expression Omnibus GSE39462, Proteomics data: PRIDE PXD000208 etc.) Please refer to our author guidelines for 'Data Deposition'.<br><br>Data deposition in a public repository is mandatory for:<br>a. Protein, DNA and RNA sequences<br>b. Macromolecular structures<br>c. Crystallographic data for small molecules<br>d. Functional genomics data<br>e. Proteomics and molecular interactions | No data deposition required |
| 19. Deposition is strongly recommended for any datasets that are central and integral to the study; please consider the journal's data policy. If no structured public repository exists for a given data type, we encourage the provision of datasets in the manuscript as a Supplementary Document (see author guidelines under 'Expanded View' or in unstructured repositories such as Dryad (see link list at top right) or Figshare (see link list at top right). | Supplementary data and Expanded view will be submitted |
| 20. Access to human clinical and genomic datasets should be provided with as few restrictions as possible while respecting ethical obligations to the patients and relevant medical and legal issues. If practically possible and compatible with the individual consent agreement used in the study, such data should be deposited in one of the major public access-controlled repositories such as dbGAP (see link list at top right) or EGA (see link list at top right). | N/A |
| 21. Computational models that are central and integral to a study should be shared without restrictions and provided in a machine-readable form. The relevant accession numbers or links should be provided. When possible, standardized format (SBML, CellML) should be used instead of scripts (e.g. MATLAB). Authors are strongly encouraged to follow the MIRIAM guidelines (see link list at top right) and deposit their model in a public database such as Biomodels (see link list at top right) or JWS Online (see link list at top right). If computer source code is provided with the paper, it should be deposited in a public repository or included in supplementary information. | N/A |

## G- Dual use research of concern

| | |
|---|---|
| 22. Could your study fall under dual use research restrictions? Please check biosecurity documents (see link list at top right) and list of select agents and toxins (APHIS/CDC) (see link list at top right). According to our biosecurity guidelines, provide a statement only if it could. | N/A |
